# Quantitative DNA Repair Biomarkers and Immune Profiling for Temozolomide and Olaparib in Metastatic Colorectal Cancer

**DOI:** 10.1158/2767-9764.CRC-23-0045

**Published:** 2023-06-28

**Authors:** Michael Cecchini, Janie Y. Zhang, Wei Wei, Jeffrey Sklar, Jill Lacy, Minghao Zhong, Yong Kong, Hongyu Zhao, Jassim DiPalermo, Lesley Devine, Stacey M. Stein, Jeremy Kortmansky, Kimberly L. Johung, Ranjit S. Bindra, Patricia LoRusso, Kurt A. Schalper

**Affiliations:** 1 Department of Internal Medicine (Medical Oncology), Yale University School of Medicine, New Haven, Connecticut.; 2 Department of Medicine, Division of Hematology/Oncology, University of Pittsburgh, Pittsburgh, Pennsylvania.; 3 Department of Biostatistics, Yale School of Public Health, New Haven, Connecticut.; 4 Department of Pathology, Yale University School of Medicine, New Haven, Connecticut.; 5 Department of Laboratory Medicine, Yale University School of Medicine, New Haven, Connecticut.; 6 Department of Therapeutic Radiology, Yale University School of Medicine, New Haven, Connecticut.

## Abstract

**Purpose:**

O^6^-methylguanine DNA methyltransferase (*MGMT*)-silenced tumors reveal sensitivity to temozolomide (TMZ), which may be enhanced by PARP inhibitors. Approximately 40% of colorectal cancer has *MGMT* silencing and we aimed to measure antitumoral and immunomodulatory effects from TMZ and olaparib in colorectal cancer.

**Experimental Design:**

Patients with advanced colorectal cancer were screened for *MGMT* promoter hypermethylation using methylation-specific PCR of archival tumor. Eligible patients received TMZ 75 mg/m^2^ days 1–7 with olaparib 150 mg twice daily every 21 days. Pretreatment tumor biopsies were collected for whole-exome sequencing (WES), and multiplex quantitative immunofluorescence (QIF) of MGMT protein expression and immune markers.

**Results:**

*MGMT* promoter hypermethylation was detected in 18/51 (35%) patients, 9 received study treatment with no objective responses, 5/9 had stable disease (SD) and 4/9 had progressive disease as best response. Three patients had clinical benefit: carcinoembryonic antigen reduction, radiographic tumor regression, and prolonged SD. MGMT expression by multiplex QIF revealed prominent tumor MGMT protein from 6/9 patients without benefit, while MGMT protein was lower in 3/9 with benefit. Moreover, benefitting patients had higher baseline CD8^+^ tumor-infiltrating lymphocytes. WES revealed 8/9 patients with MAP kinase variants (7 *KRAS* and 1 *ERBB2*). Flow cytometry identified peripheral expansion of effector T cells.

**Conclusions:**

Our results indicate discordance between *MGMT* promoter hypermethylation and MGMT protein expression. Antitumor activity seen in patients with low MGMT protein expression, supports MGMT protein as a predictor of alkylator sensitivity. Increased CD8^+^ TILs and peripheral activated T cells, suggest a role for immunostimulatory combinations.

**Significance:**

TMZ and PARP inhibitors synergize *in vitro* and *in vivo* in tumors with MGMT silencing. Up to 40% of colorectal cancer is MGMT promoter hypermethylated, and we investigated whether TMZ and olaparib are effective in this population. We also measured MGMT by QIF and observed efficacy only in patients with low MGMT, suggesting quantitative MGMT biomarkers more accurately predict benefit to alkylator combinations.

## Introduction

Colorectal cancer remains highly prevalent with few available biomarker-guided therapies. However, promoter hypermethylation of O^6^-methylguanine DNA methyltransferase (*MGMT*) is identified in approximately 40% of metastatic colorectal cancer (1, 2). Hypermethylation of the *MGMT* promoter is expected to result in decreased MGMT mRNA/protein expression, which reduces the capacity of tumor cells to repair the lethal O^6^-methylguanine lesions produced by alkylating agents and renders these tumors more susceptible to these agents, including temozolomide (TMZ; refs. 3–5). Moreover, the base excision repair pathway is a parallel process of alkylator repair and preliminary studies suggest that TMZ in combination with PARP inhibitors (PARPi) may enhance tumor cell death in *MGMT*-silenced tumors (6–8). The increased sensitivity of tumors to TMZ with a PARPi is multifactorial and includes the presence of stalled replication forks typically overcome by homologous recombination (HR) DNA repair, and PARPi can delay the initiation of this HR-mediated recovery (7). Furthermore, “PARP trapping” onto DNA is important for PARPi + TMZ sensitivity, which points to olaparib as an ideal candidate to use in combination with TMZ (7–10).

These preclinical findings suggesting enhanced TMZ sensitivity with PARPi have led to several clinical trials in different tumor types. One example, a prior clinical trial of TMZ with veliparib in patients with advanced colorectal cancer reported a disease control rate (DCR) of 24% after two cycles (11). This study did not select patients with MGMT promoter hypermethylated tumors and used a PARPi with limited PARP trapping effect. The biological determinants for treatment sensitivity and resistance in this setting remain poorly explored. Here, we report the results of a single-arm, investigator-initiated, phase II clinical trial of TMZ in combination with olaparib in patients with *MGMT* promoter hypermethylated advanced colorectal cancer. We evaluated the clinical activity and explored biological determinants using genomic and protein-based biomarkers.

## Materials and Methods

### Study Design and Participants

This study was a single-arm, open-label, phase II clinical trial performed at the Yale Cancer Center. Eligible patients had histopathologic confirmation of stage IV microsatellite stable (MSS) colorectal cancer that progressed after 5-fluorouracil, oxaliplatin, irinotecan ,and appropriate biologic therapy. Promoter hypermethylation of *MGMT* was an integral biomarker for selection/enrollment and patients were prescreened using archival tumor tissue samples. The *MGMT* promoter hypermethylation was measured by methylation-specific PCR (MS-PCR) in the Clinical Laboratory Improvement Amendments (CLIA)-certified Yale Molecular Diagnostics Laboratory. As an integral biomarker for enrollment, MS-PCR was selected over other methods of *MGMT* testing such as pyrosequencing and methyl-BEAMing given the validation of MS-PCR for clinical trial samples in our institution. These alternative methods of *MGMT* testing are likely only semiquantitative given the rough estimates of tumor percentage. Furthermore, while they may offer advantages to predict response to TMZ monotherapy or TMZ/cytotoxic combinations (2, 12, 13) their predictive power is uncertain for TMZ + PARPi, which may work at different *MGMT* levels (14, 15). A full list of eligibility criteria is available as Supplementary Materials and Methods S1. All patients provided written informed consent as a condition of participation and the Yale University Institutional Review Board approved the study, which adheres to Good Clinical Practice Guidelines. The study was conducted in accordance with the Declaration of Helsinki and followed the Consolidated Standards of Reporting Trials. The ClinicalTrials.gov Identifier is NCT04166435.

### Procedures

Patients received a starting dose of TMZ 75 mg/m^2^ days 1–7 with continuous olaparib 150 mg twice daily during 21-day cycles. The standard olaparib dose of 300 mg twice daily was not used to due to the enhanced myelosuppression with PARPi-chemotherapy combinations. The TMZ was selected on the basis of prior experience with TMZ and olaparib by Farago and colleagues and we aimed to use continuous PARP inhibition for continuous PARP trapping and thus a slightly lower dose of olaparib 150 mg twice daily was selected (16). Participants received TMZ and olaparib until progression of disease, unacceptable toxicity, death or withdrawal. Tumor RECIST measurements were performed at baseline and every 6 weeks on study. The study mandated a pretreatment tumor biopsy along with an optional progression biopsy. Blood and buffy coat were collected at screening, during treatment, and on-progression.

For whole-exome sequencing (WES), DNA from the pretreatment tissue samples and patient-matched normal DNA extracted from buffy coats were sequenced. The WES was performed using Illumina NovaSeq 6000 at the Yale Center for Genome Analysis for tumor/normal pairs. A multiplex quantitative immunofluorescence (QIF) panel was standardized for simultaneous and spatially resolved measurement of DAPI (all nuclei), cytokeratin (CK; epithelial or tumor cells), MGMT protein, γH2AX, and CD8^+^ tumor-infiltrating lymphocytes (TIL) in the whole tissue sections from tumor biopsies. Antibodies for MGMT, γH2AX, and CD8 were validated for specificity and reproducibility and multiplex QIF was performed as reported previously (17, 18). A control tissue microarray sample containing positive and negative control samples was used for assay validation and stained alongside the trial samples for reproducibility assessment. Imaging was acquired on a Vectra Polaris instrument. For isolation of peripheral blood mononuclear cells (PBMC), whole blood was subjugated to density gradient separation using Ficoll-Paque PLUS. Cells were frozen in liquid nitrogen until all samples from the time course had been collected.

### Outcomes

The primary endpoint of the clinical trial was the objective response rate (ORR), defined as the proportion of patients with complete or partial response by RECIST. Secondary endpoints included progression-free survival (PFS), overall survival (OS), DCR, and safety and tolerability of the combination. Exploratory endpoints included MGMT protein expression by QIF, changes in peripheral immune cell populations, and changes in tumor mutational burden.

### Statistical Analysis

A null hypothesis of a 5% ORR was used with alternative hypothesis ORR of ≥25%. An early stopping rule with a Bayesian analysis plan for the ORR was planned for after the first 9 patients with ≥1 response required to continue enrollment to a total sample size of 30 patients. Four responses in a full cohort of 30 patients were required to meet the primary endpoint. Survival functions were compared using Kaplan–Meier graphical analysis and the log-rank test. Comparisons between continuous QIF scores across groups were conducted using the Mann–Whitney test. Patient characteristics were compared using the Student *t* test for continuous variables and *χ*^2^ test for categorical variables. The statistical analysis and graphical representation was performed in Graphpad Prism v7.01 for windows (GraphPad Software, Inc). All two-tailed *P* values ≤0.05 were considered as statistically significant.

### Data Availability Statement

The genomic data generated in this study are publicly available in Sequence Read Archive BioProject ID PRJNA956444. Non-genomic data will be made available based on reasonable request to the corresponding author.

## Results

**FIGURE 1:**
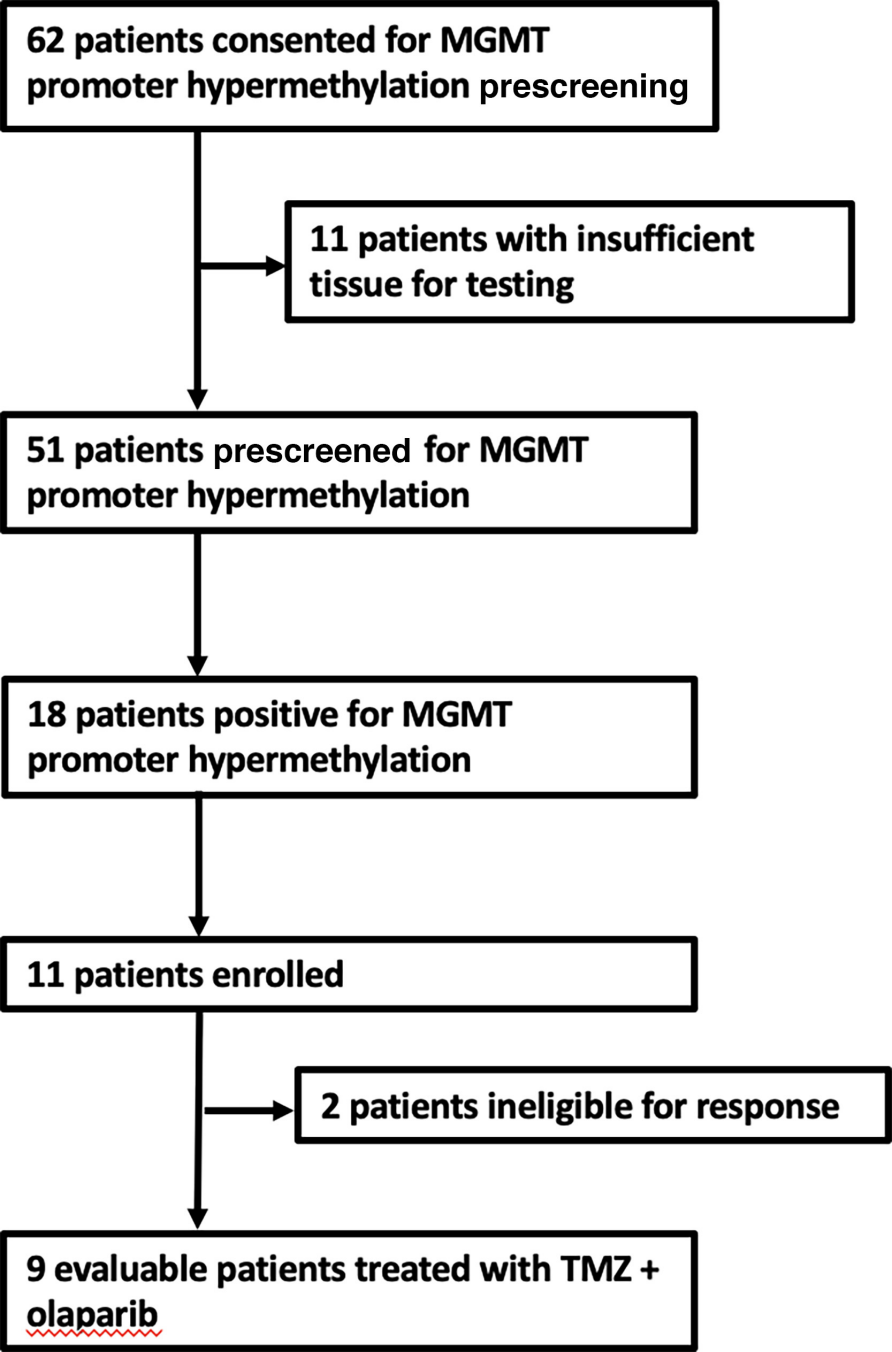
Trial profile. CONSORT diagram for enrolled participants.

**FIGURE 2:**
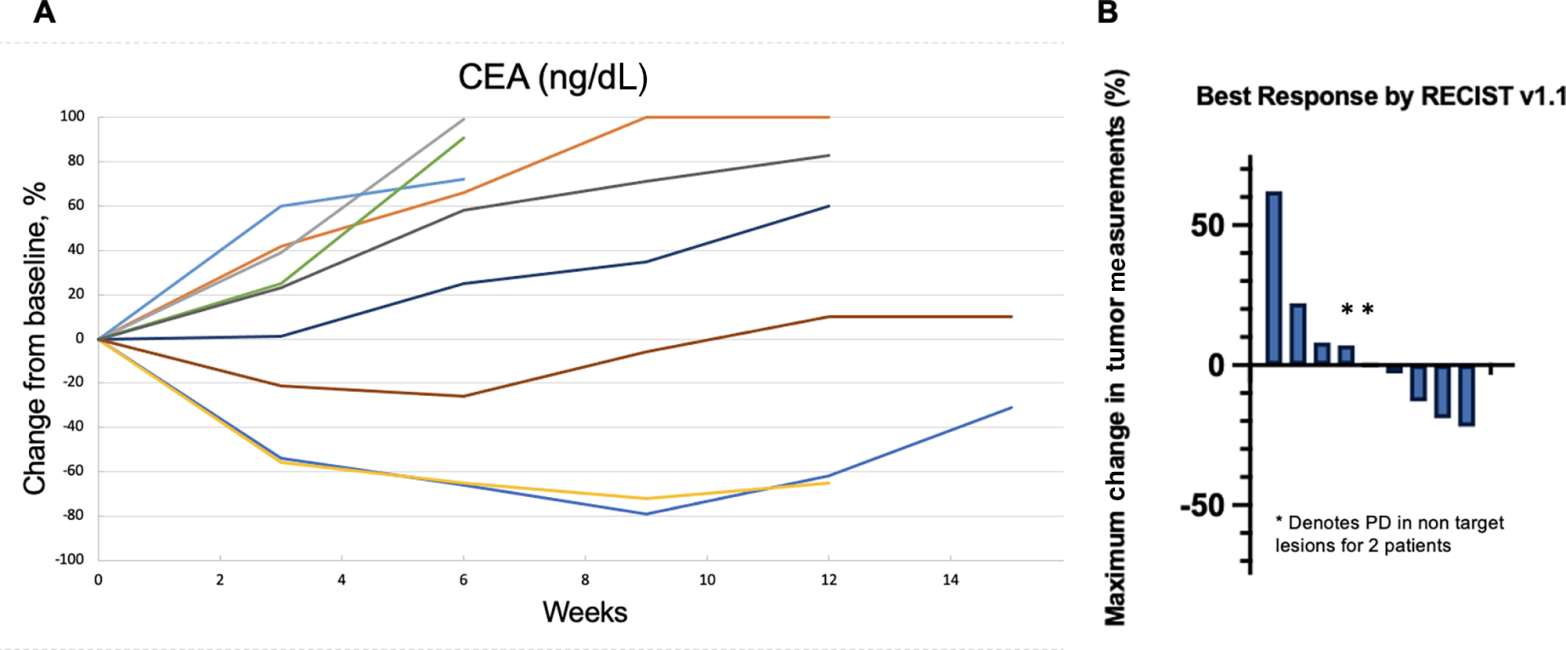
Biochemical and radiographic response. **A,** Spider plot of the percent change of CEA (ng/dL) from baseline for evaluable patients. Each color represents a patient. **B,** Waterfall plot for all evaluable patients, representing radiographic best response of tumor change from baseline according to RECIST v1.1. The * denotes patients that had progression of non-target lesions at the time of disease progression.

Between February 19, 2020 and June 22, 2021, 62 patients with metastatic MSS colorectal cancer were screened for *MGMT* promoter hypermethylation, of which 51 had adequate tumor tissue for clinical grade *MGMT* testing by bisulfite deamination and MS-PCR. We identified *MGMT* promoter hypermethylation in 18/51 (35%) of patient samples (Fig. 1). Nine patients received TMZ with olaparib due to the predefined stopping rule and their baseline characteristics are outlined in Table 1. No patients had a confirmed partial or complete response, 5/9 (56%) had a best response of stable disease (SD) and 4/9 (44%) had progressive disease, and the study closed early per the predefined stopping rule. For those patients with SD by RECIST, 3 patients had reductions in carcinoembryonic antigen (CEA) of ≥25% (Fig. 2A) and ≥10% tumor regression by RECIST (Fig. 2B). The median PFS was 3.0 months (95% CI: 2.1–not reached) and median OS was 9.4 months (95% CI: 6.7–not reached; Supplementary Fig. S1A and S1B). Treatment-related adverse events are summarized in Table 2. Five patients underwent dose modifications for treatment-related adverse events, 3 for grade 3 neutrophil count decrease, 1 for grade 3 platelet count decrease, and 1 for grade 3 mucositis. For analysis of correlative studies, the cases were divided in two clinical groups including patients who had clinical benefit as defined by CEA reduction, minor radiographic tumor regression by RECIST v1.1 (>10%) and prolonged SD by RECIST v1.1 (≥100 days), and those who did not experience clinical benefit (e.g., progressive disease). A study representativeness table is provided by Supplementary Table S1.

**TABLE 1:** Baseline characteristics

Characteristic	TMZ + Olaparib (*N* = 9)
Age	
Median	59
Range	45–78
Sex – no. (%)	
Male	4 (44)
Female	5 (56)
Race – no. (%)	
White	8 (89)
Black	1 (11)
ECOG performance status – no. (%)	
0	3 (33)
1	6 (67)
Side of primary tumor^a^ – no. (%)	
Left	4 (44)
Right	5 (56)
Tumor grade – no. (%)	
Poorly differentiated	3 (33)
Moderately differentiated	6 (67)
Molecular results – no. (%)	
*KRAS* mutated	7 (78)
*KRAS/RAF* wildtype	2 (22)
Microsatellite stable	9 (100)
Number of prior therapies – no. (%)	
2	2 (22)
≥3	7 (78)

^a^Right side defined as tumors primary to splenic flexure.

**TABLE 2:** Frequency of treatment-related adverse events and laboratory abnormalities

Event	Any grade	Grade ≥3
Any event – no. (%)	9 (100)	7 (78)
Any serious event – no. (%)^a^	0	0
Most common events – no. (%)		
Nausea	3 (34)	0
Fatigue	2 (22)	0
Anorexia	2 (22)	0
Constipation	1 (11)	0
Mucositis	1 (11)	1 (11)
Concentration impairment		
Laboratory abnormalities – no. (%)		
White blood cell count decreased	6 (67)	3 (34)
Neutrophil count decrease	5 (56)	4 (44)
Platelet count decreased	5 (56)	2 (22)
Anemia	4 (44)	0
Lymphocyte count decrease	3 (34)	2 (22)
Aspartate aminotransferase increased	2 (22)	0
Alkaline phosphatase increased	1 (11)	0

^a^One serious adverse event was reported for one patient nausea/vomiting that was related to underlying disease.

Whole-exome DNA sequencing was performed on tumor and matched non-tumor samples from all patients who received study treatments. The mean coverage was 486 reads and the mean number of nonsynonymous mutations across cases was 527 (range, 279–527). Molecular analysis revealed 8/9 (89%) had tumor-specific deleterious variants in genes of the MAP kinase pathway (7 *KRAS* and 1 *ERBB2*), and 8/9 (89%) had *TP53* variants (Supplementary Fig. S2). The mean tumor mutational burden was 15 mutations/megabase (range, 6–41) as outlined in Supplementary Fig. S2A. One patient without any clinical benefit had a BRCA2 mutation, and no HR-related mutations were identified in any of the patients who experienced clinical benefit. Given the limited sample size no association was noted between patient outcomes and genomic alterations.

**FIGURE 3:**
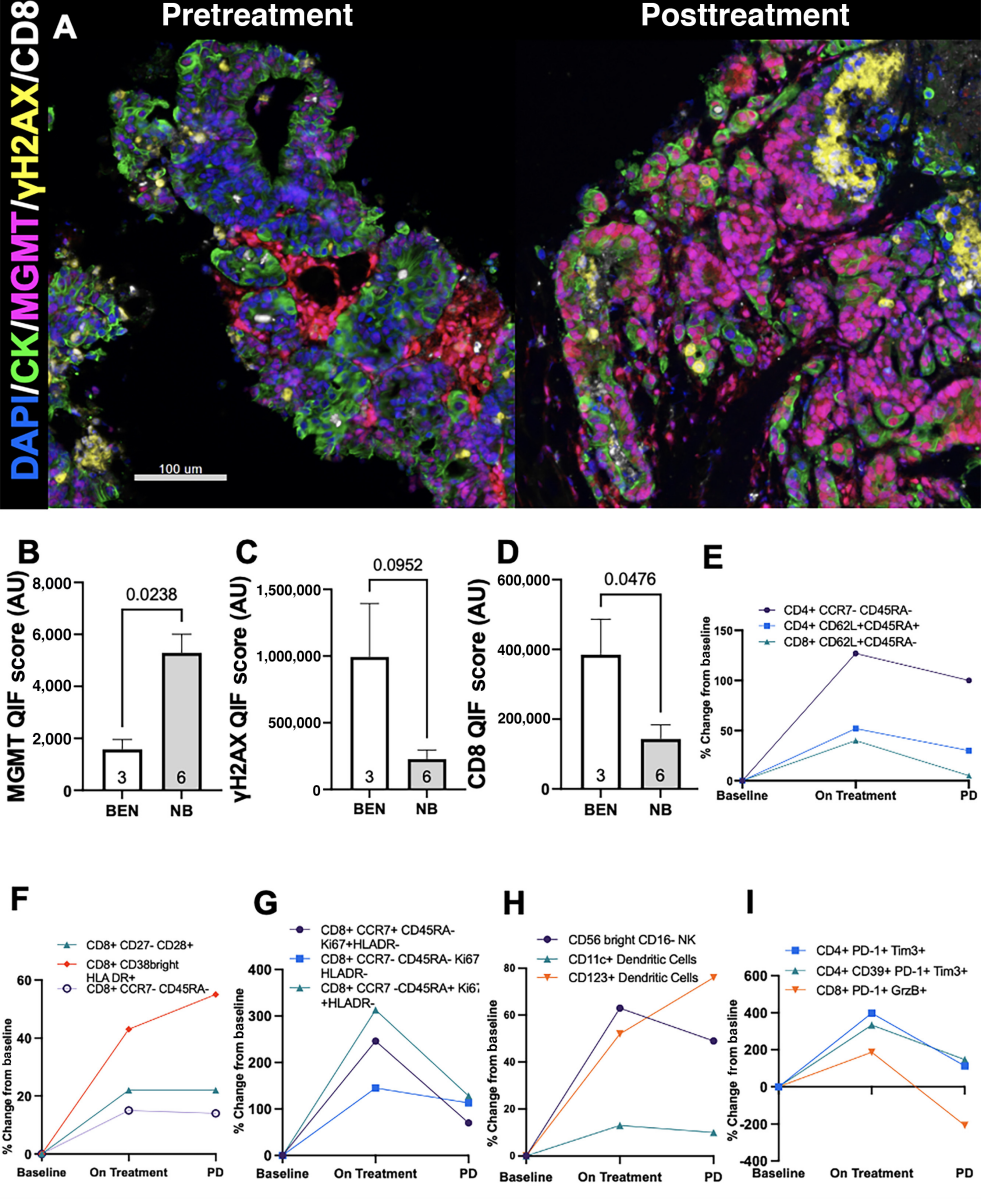
Low tumor-specific MGMT protein expression and high CD8^+^ T-cell infiltration are seen in the pretreatment biopsies of patients who derived clinical benefit from the trial and trial therapy alters the PBMC population. **A,** Representative multicolor images of a pretreatment biopsy sample with tumor cell–selective MGMT downregulation and paired posttreatment biopsy with increased tumor-specific MGMT expression. Epithelial and tumor cells are highlighted by CK (green channel). MGMT-positive cells are shown in the red channel, γH2AX-positive cells in the yellow channel, and CD8-positive cells in the white channel. Bar = 100 μm. Mean levels of MGMT (**B**), γH2AX (**C**), and CD8 (**D**) in the pretreatment biopsies of patients who derived clinical benefit on trial (white bars) and who did not show any clinical benefit (gray bars). Differences between groups were compared using the nonparametric Mann–Whitney test. The *P* values obtained for marker comparisons are indicated within each chart. **E**–**I,** The change from baseline mean for each cell population was plotted for two timepoints (i) on-treatment (cycle 2) and (ii) progression by RECIST. The maximal positive/negative mean % change values are plotted for five different panels; (E) Basic Immunophenotype panel; (F) Memory-Naïve and Activated T Cell panel; (G) T Cell Proliferation panel; (H) Monocytes/DC/NK Cell panel; and (I) T Cell Exhaustion panel.

To obtain a quantitative and spatially resolved assessment of MGMT protein expression, DNA damage response (DDR), and TILs in pretreatment and posttreatment tumors, we studied the biopsy samples with a 5-colored multiplexed QIF panel containing the markers DAPI for all cells, cytokeratin for tumor cells, MGMT, γH2AX as a marker of DDR, and CD8 for cytotoxic T cells (representative example: Fig. 3A). The marker scores were selectively measured in tumor and non-tumor stromal cells based on their colocalization with the epithelial/tumor cell marker cytokeratin as reported previously (18). Despite showing *MGMT* promoter hypermethylation by MS-PCR, MGMT protein was detected in cytokeratin-positive tumor cells from six of the nine baseline biopsy samples. Repeat bisulfite deamination and MS-PCR of the *MGMT* promoter was conducted as confirmation on all pretreatment biopsies and all 9 patients remained *MGMT* promoter-hypermethylated at enrollment.

Despite the limited number of cases, the tumor cell–specific MGMT protein expression in the pretreatment biopsies of the 3 patients who derived clinical benefit from study treatment was significantly lower compared with the pretreatment biopsies of the 6 patients who did not experience disease stabilization on study (Fig. 3B). The cases with clinical activity showed also numerically higher levels of γH2AX in tumor cells and significantly higher CD8^+^ TILs (Fig. 3C and D). Two patients with clinical benefit underwent biopsy at the time of disease progression with 1 patient biopsied at the same site for both pretreatment and progression and another patient with a pretreatment lymph node biopsy and progression biopsy of peritoneal metastasis. Both patients remained mismatch repair proficient on progression biopsy. The progression biopsies showed marked increased in tumoral MGMT protein levels relative to the pretreatment biopsy and a trend toward decreased γH2AX and CD8^+^ T-cell tumor infiltration (Supplementary Fig. S3). Together, these results suggest that MGMT protein can be expressed in a substantial fraction of *MGMT* promoter-methylated colorectal cancer, and low MGMT expression in tumor cells and increased local effector TILs were associated with benefit from TMZ and olaparib. Conversely, elevated tumoral-cell MGMT protein expression was associated with lack of any clinical benefit from the treatment regimen, supporting the value of this metric as a biomarker.

Peripheral blood was collected to assess the pharmacodynamic effects of the study treatment on PBMC. For each PBMC population (Fig. 3E–I) the baseline mean for all patients was calculated and the percent change from baseline mean was measured on cycle 2 day 1 (“On Treatment”) and at the time of progression for all patients. Most prominently, an expansion and proliferation of recently activated CD8^+^ T cells was seen with treatment, as well as increases in both naïve and memory T cells (Fig. 3E–G). There was also an expansion of tumor-associated natural killer (NK) cells (CD56bright CD16^−^), and an increase in multiple T-cell exhaustion markers (Fig. 3H and I). A classification of the immune cell subtype from the flow cytometry results is available in Supplementary Table S2.

## Discussion

Our study represents the first clinical evaluation of TMZ plus a PARPi in *MGMT* promoter hypermethylated colorectal cancer. Although, no responses by RECIST v1.1 were observed, correlative studies suggest potential for further evaluation of this therapy by refining the biomarker selection strategy. Moreover, this is the first prospective assessment of the MGMT protein levels, γH2AX and TILs using spatially resolved quantitative analysis in this setting. Here we found prominent discordance between the *MGMT* promoter hypermethylation status and MGMT protein expression in colorectal cancers and the associated clinical outcomes. Furthermore, in patients with clinical benefit, increased MGMT protein expression at progression was noted, representing a potential resistance mechanism to study treatment. Our data suggest that localized MGMT protein measurements could be a superior biomarker than *MGMT* methylation by MS-PCR in this population and tumors with low MGMT protein and high CD8^+^ TILs derived increased clinical benefit from TMZ plus olaparib combination therapy. Flow cytometry of PBMCs also revealed an expansion in activated CD8^+^ T cells after treatment with TMZ plus olaparib (Supplementary Fig. S3). Our findings of an increase in CD8^+^ TILs and PBMCs may support the potential investigation of alkylator-immunotherapy combinations in MGMT silenced tumors.

The antitumoral activity for approved agents in the third line setting for colorectal cancer is extremely limited with response rates of approximately 1% with regorafenib and TAS-102 (19, 20). Furthermore, our patient population was heavily pretreated with 7/9 (78%) of patients receiving at least three prior therapies, likely making it more difficult to achieve a radiographic response. Thus, while we did not observe an objective response, the reductions in target lesions described in Fig. 2 are noteworthy and consistent with treatment activity in a subset of patients. It is also possible that the lower dose of olaparib may have limited the efficacy, but 300 mg twice daily olaparib is intolerable in a chemotherapy combination.

Prior to our clinical trial evaluating TMZ and olaparib in colorectal cancer, a previous clinical evaluated TMZ with veliparib, a PARPi (11). However, veliparib does not “trap” PARP like other PARPis, such as olaparib, and is presumed to be inferior to PARPis with the ability to PARP-“trap” when used in combination with TMZ (7–10). Moreover, this study did not restrict enrollment to *MGMT* promoter hypermethylated colorectal cancer as our study did, nor did it analyze MGMT expression and TILs. Recently, data from Morano and colleagues in the MAYA trial described the combination of TMZ and low-dose ipilimumab/nivolumab in patients with MSS colorectal cancer, indicating a very promising ORR and survival with the combination (21). These observations were further supported by the ARETHUSA trial, which treated patients with colorectal cancer with “TMZ priming” followed by pembrolizumab and revealed acquired *MSH6* variants, a genomic signature for TMZ, and increased tumor mutational burden after the “TMZ priming” (22). While preliminary, the MAYA and ARETHUSA trials suggests immunogenicity with TMZ treatment, and support further novel strategies to use alkylators together with immunostimulatory therapies. However, in glioma, TMZ-induced hypermutation has not translated into enhanced sensitivity to immune checkpoint inhibitors, which highlights the need for additional biomarkers (23). The results from our clinical trial also support the concept that tumors with lower levels of MGMT are more immunogenic given our observations of higher CD8^+^ T-cell levels in MGMT low tumors and that TMZ may enhance the antitumor immune response by a pharmacodynamic effect of increased immunostimulatory PBMCs. These observations are novel and have not been described in previous TMZ single monotherapy or combination strategies, and the role that the addition of olaparib may play in the pharmacodynamic PBMCs effects will require further study. Additional exploratory descriptive analysis of the PBMC changes for patients with clinical benefit versus no benefit is outlined in Supplementary Fig. S4, which are limited by the small sample size, but do suggest an increase in multiple PBMC populations for the clinical benefit group.

Our genomic analysis revealed *APC* to be among the most commonly mutated genes, which is consistent with the typical molecular profile of colorectal cancer. In contrast, previous observations for *MGMT* promoter hypermethylation in colorectal cancer were that these tumors are more likely to arise from serrated adenomas and serrated adenocarcinomas, which are less frequently *APC*-mutated (24, 25). Thus, *MGMT* promoter hypermethylated colorectal cancer may be heterogenous in development compared with the conventional development of adenomatous polyps. The elevated tumor mutational burden of 15 mutations/megabase, may suggest these tumors have increased levels of tumor neoantigens and therefore more immunogenic, which could be due to impaired DNA repair in the setting of *MGMT* promoter hypermethylation. The high prevalence of MAP kinase mutations (*KRAS* and *ERBB2*) is important should future biologic therapy be added to TMZ combinations, and support the use of bevacizumab over cetuximab/panitumumab. Moreover, tumor hypoxia induced by bevacizumab may sensitize tumors to PARPis by creating homologous recombination deficiency (26, 27).

For metastatic colorectal cancer, the successful use of alkylator combinations in the future will require a careful selection of biomarkers, which should include a quantitative measure of MGMT protein expression, as supported by our findings. Comparable findings are described by other groups showing that *MGMT* promoter hypermethylation is necessary but not sufficient for TMZ sensitivity for colorectal cancer (21). We confirm this observation by showing a lack of correlation between these two markers in the current study and in our related presented work (28). Thus, a future direction of TMZ and PARPi combination could be in patients with specifically low tumoral MGMT protein levels.

The major limitation of our study is the small sample size of patients that received TMZ and olaparib. While we did identify *MGMT* promoter hypermethylation in 18/51 (35%) prescreened patients, only 9 received the study treatment due to the predefined early stopping rule. Furthermore, we assessed *MGMT* testing by MS-PCR which is our CLIA lab's validated *MGMT* test, but it is possible that alternative *MGMT* promoter hypermethylation testing such as methyl-BEAMing or pyrosequencing would have better enriched our cohort for response (2, 12, 13). Our correlative analysis is also limited by a lack of on-treatment or progression biopsies to study changes in the tumor microenvironment after treatment with TMZ and olaparib.

In conclusion, TMZ and olaparib was tolerable and did reveal antitumor activity in a subset of patients with *MGMT* promoter hypermethylated tumors that also had low MGMT protein expression and increased CD8^+^ effector TILs. However, given the lack of response in the overall treatment group, this did not justify continuing enrollment beyond 9 patients. Nevertheless, it was noteworthy that *MGMT* promoter hypermethylation was seen in 35% of prescreened patients, and treated patients had higher than expected MAPK signaling alterations. Furthermore, the treatment of TMZ plus olaparib has a noticeable impact on activated CD8^+^ T cells in patients with advanced colorectal cancer, and therefore novel alkylator-immunotherapy combinations may be warranted.

## Supplementary Material

Supplementary Figure 1 Supplementary Figure 1: Survival Analysis.

Supplementary Figure 2 Supplementary Figure 2: Molecular Analysis

Supplementary Figure 3 Supplementary Figure 3: Changes in MGMT expression, yH2AX and CD8+ T cells in pre-treatment and post-treatment biopsies of patients who derived clinical benefit from the trial.

Supplementary Figure 4 Supplementary Figure 4: The change in PBMCs for patients with clinical benefit (blue) and no benefit (red).

Supplementary Table 1 Supplementary Table 1: Study representativeness table.

Supplementary Table 2 Supplementary Table 2: A classification of the immune cell subtypes.

Supplementary Methods 1 Supplementary Methods 1: Clinical trial protocol.
