# Supplementary figures and images for: Quantitative DNA Repair Biomarkers and Immune Profiling for Temozolomide and Olaparib in Metastatic Colorectal Cancer
Source: Cancer Res Commun. 2023 Jun 28;3(6):1132–9. doi: 10.1158/2767-9764.CRC-23-0045 (PMC10305782; doi:10.1158/2767-9764.CRC-23-0045)

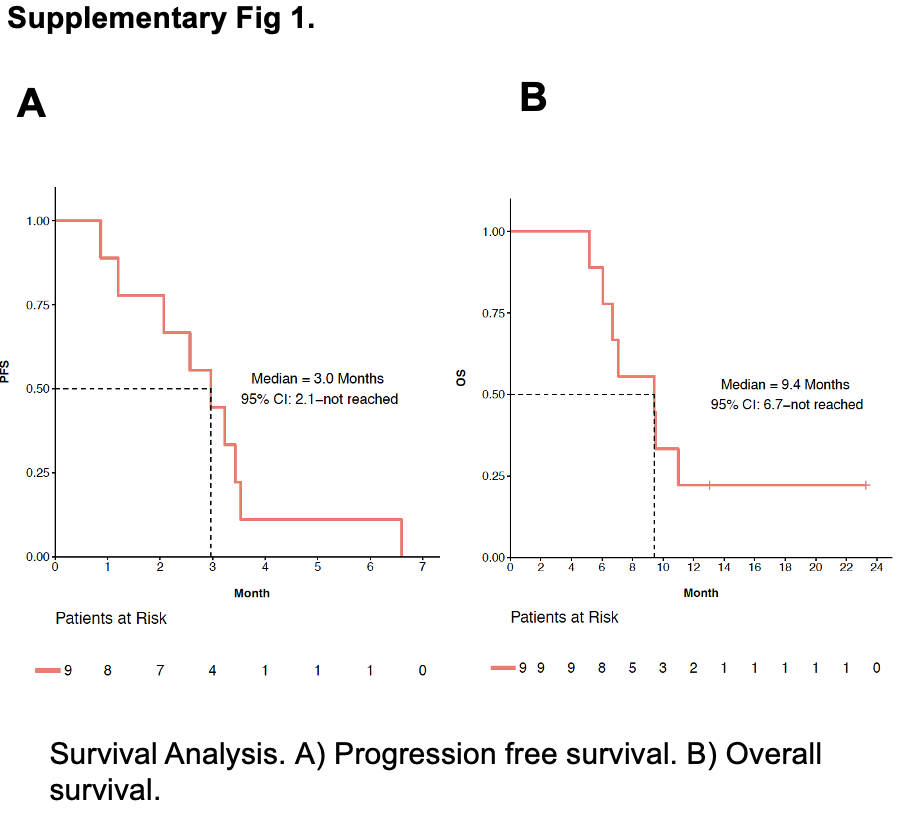

Supplement: Supplementary Figure 1 — Survival Analysis. [file crc-23-0045-s01.png]

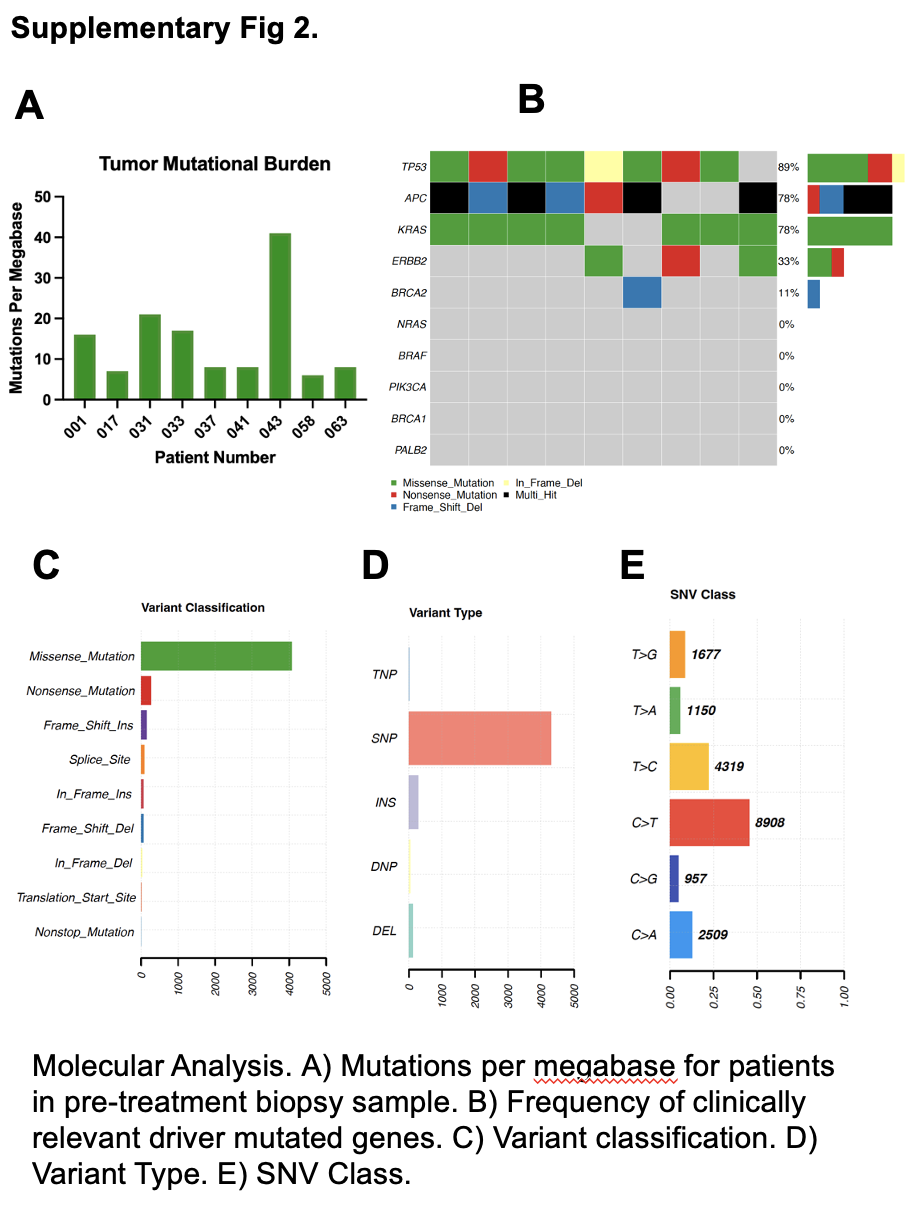

Supplement: Supplementary Figure 2 — Molecular Analysis [file crc-23-0045-s02.png]

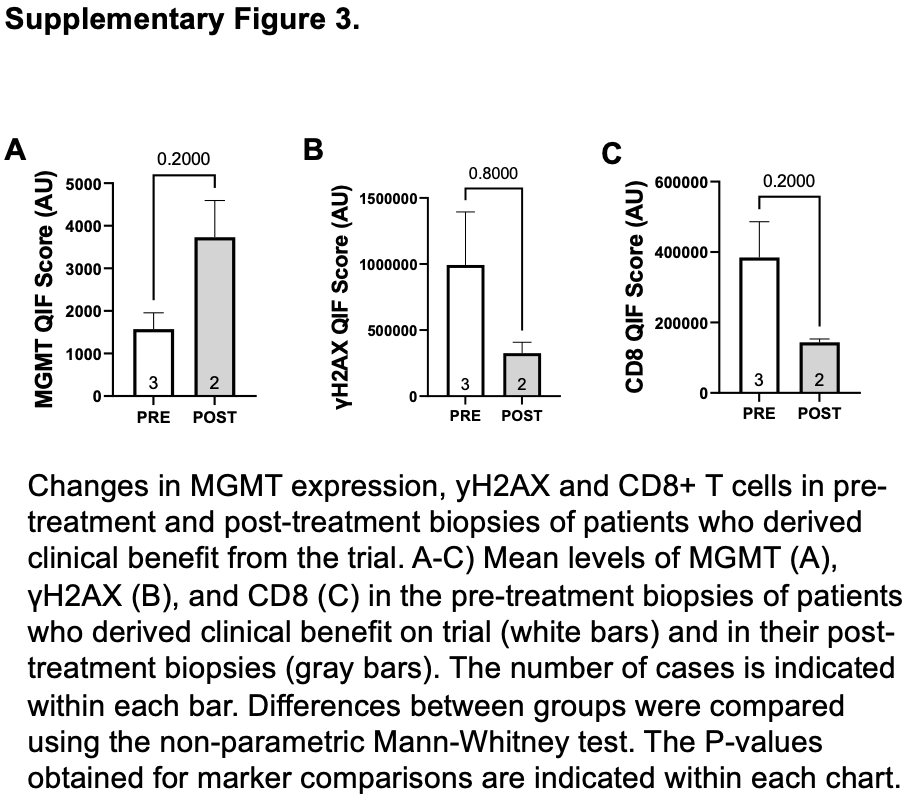

Supplement: Supplementary Figure 3 — Changes in MGMT expression, yH2AX and CD8+ T cells in pre-treatment and post-treatment biopsies of patients who derived clinical benefit from the trial. [file crc-23-0045-s03.png]

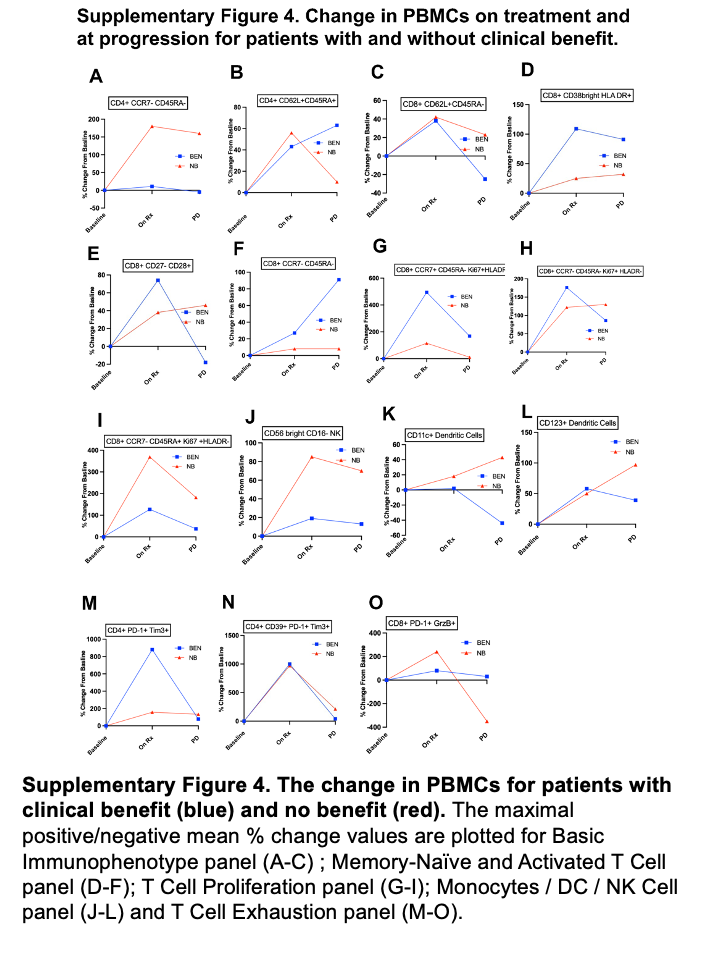

Supplement: Supplementary Figure 4 — The change in PBMCs for patients with clinical benefit (blue) and no benefit (red). [file crc-23-0045-s04.png]

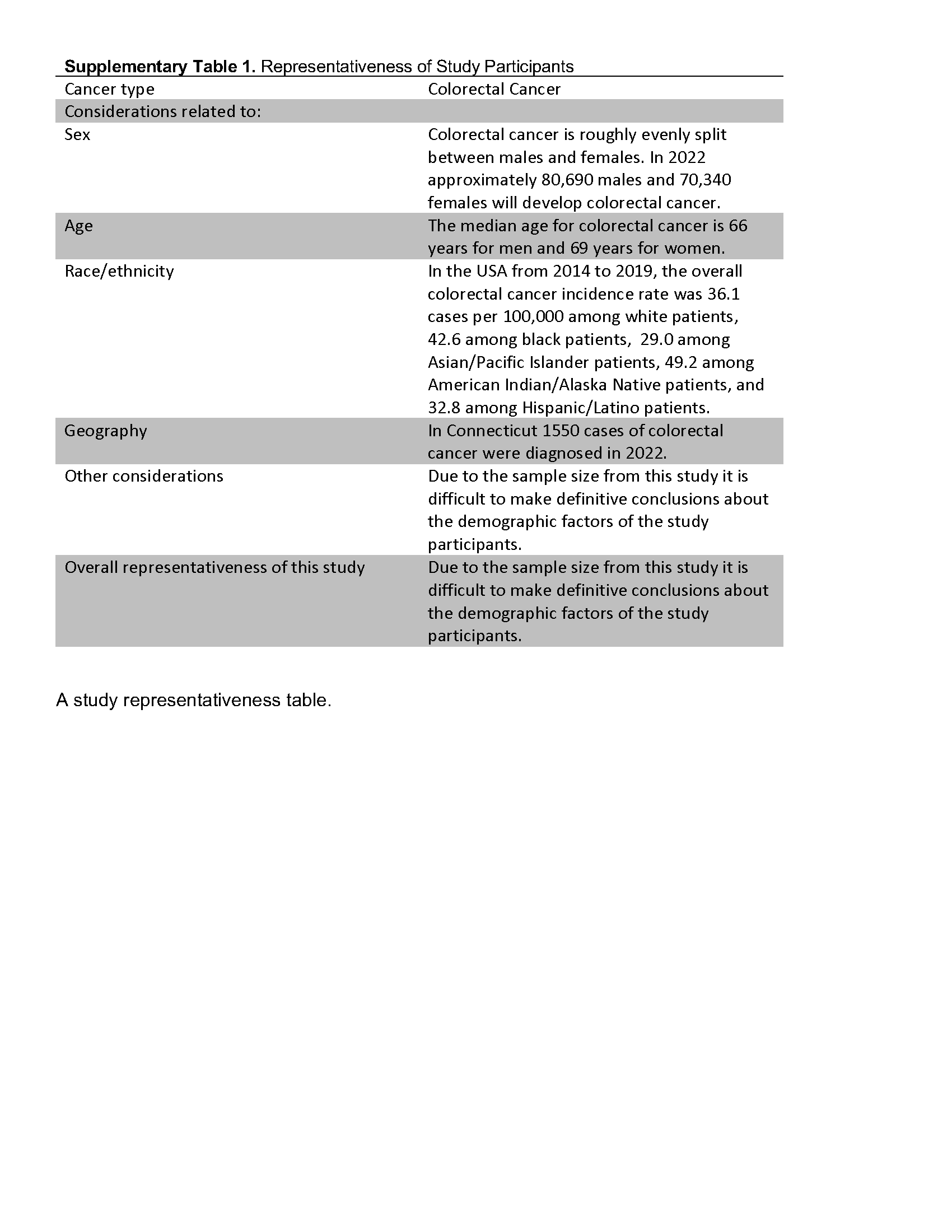

Supplement: Supplementary Table 1 — Study representativeness table. [file crc-23-0045-s05.png]

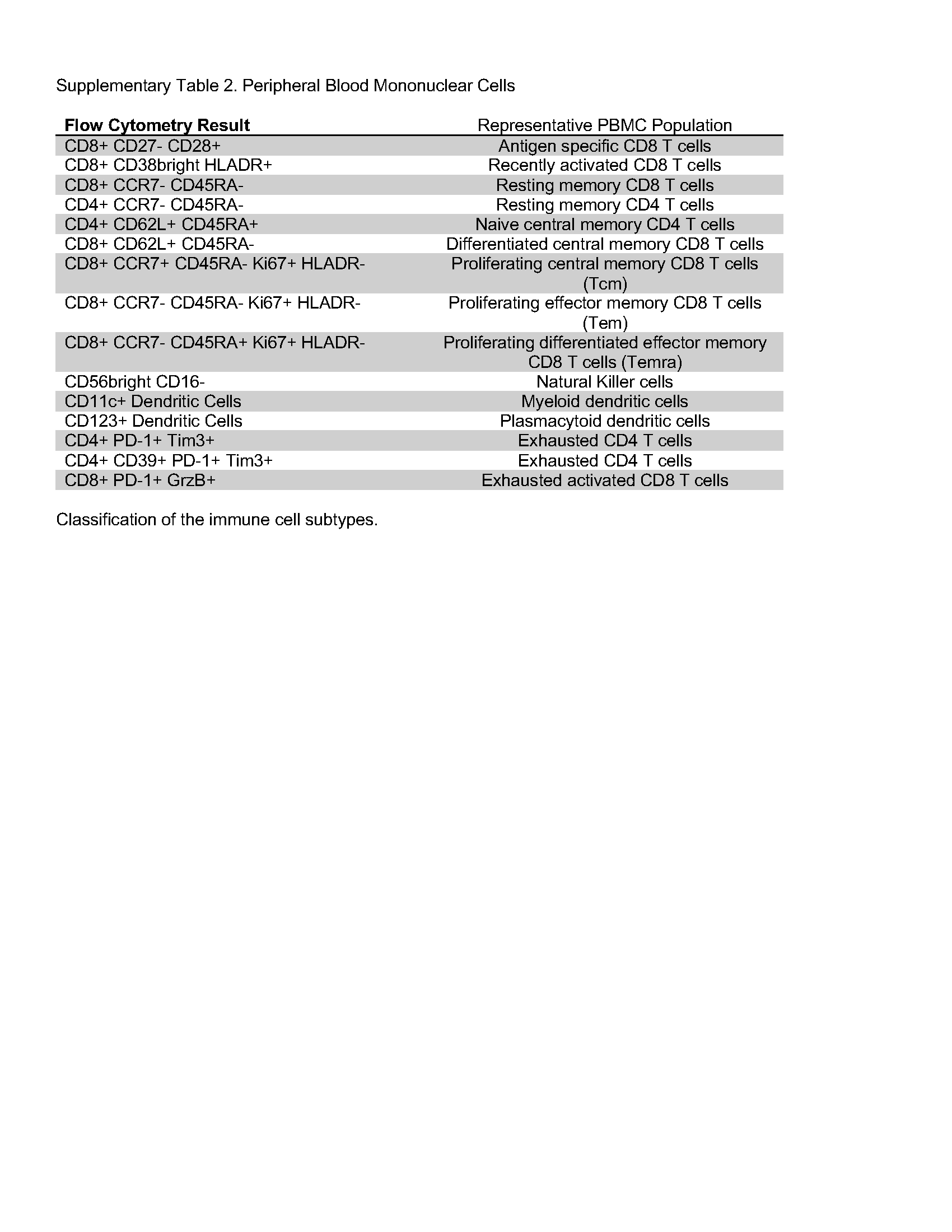

Supplement: Supplementary Table 2 — A classification of the immune cell subtypes. [file crc-23-0045-s06.png]
