# Supplementary material for: Quantitative DNA Repair Biomarkers and Immune Profiling for Temozolomide and Olaparib in Metastatic Colorectal Cancer
Source: Cancer Res Commun. 2023 Jun 28;3(6):1132–9. doi: 10.1158/2767-9764.CRC-23-0045 (PMC10305782; doi:10.1158/2767-9764.CRC-23-0045)
Supplement: Supplementary Methods 1 — Clinical trial protocol. [file crc-23-0045-s07.pdf]

Supplementary Methods 1

APPROVED BY THE YALE UNIVERSITY IRB 2/12/2020

Clinical Study Protocol	
Drug Substance	Olaparib
Study Code	<<>>
Version	3.1
Date	11/11/19

Temozolomide and Olaparib for O^6-Methyguanine DNA Methyltransferase Promoter Hypermethylated Colorectal Cancer

APPROVED BY THE YALE UNIVERSITY IRB 2/12/2020

APPROVED BY THE YALE UNIVERSITY IRB 2/12/2020

VERSION HISTORY

Version VERSION 3.1, 11/11/19
Version 1.0, 6/6/19<br>Version 2.0, 8/1/19<br>Version 3.0, 8/26/2019

APPROVED BY THE YALE UNIVERSITY IRB 2/12/2020

TABLE OF CONTENTS

TITLE PAGE 1
VERSION HISTORY 2
TABLE OF CONTENTS 3
1. PROTOCOL SUMMARY 8
1.1 Schedule of Activities (SoA) 8
1.2 Synopsis 14
1.3 Schema 18
2. INTRODUCTION 19
2.1 Study rationale 20
2.2 Background 20
2.3 Benefit/risk assessment 23
2.3.1 Potential benefits and risks for olaparib 23
2.3.2 Potential benefits and risks for temozolomide 24
3. OBJECTIVES AND ENDPOINTS 25
4. STUDY DESIGN 26
4.1 Overall design 26
4.2 Safety Evaluation Period 27
4.3 Dose Limiting Toxicity Definition 27
4.4 Scientific rationale for study design 28
4.5 Justification for dose 28
4.6 End of study definition 28
5. STUDY POPULATION 29
5.1 Inclusion criteria 29
5.2 Exclusion criteria 31
5.3 Lifestyle restrictions 33
5.3.1 Meals and dietary restrictions 33
5.3.2 Activity 33
5.4 Screen failures 34
6. STUDY TREATMENTS 34
6.1 Treatments administered 34
6.1.1 Investigational products 34

APPROVED BY THE YALE UNIVERSITY IRB 2/12/2020

APPROVED BY THE YALE UNIVERSITY IRB 2/12/2020

6.2 Preparation/handling/storage/accountability 35
6.3 Measures to minimise bias: randomisation and blinding 36
6.4 Treatment compliance 36
6.5 Concomitant therapy 36
6.5.1 Background medication 40
6.5.2 Other concomitant treatment 40
6.6 Dose Modifications 40
7. DISCONTINUATION OF TREATMENT AND PATIENT WITHDRAWAL 42
7.1 Discontinuation of study treatment 42
7.1.1 Procedures for discontinuation of study treatment 42
7.2 Lost to follow-up 43
7.3 Withdrawal from the study 43
8. STUDY ASSESSMENTS AND PROCEDURES 44
8.1 Efficacy assessments 44
8.2 Safety assessments 45
8.2.1 Clinical safety laboratory assessments 45
8.2.1.1 Coagulation 46
8.2.1.2 Bone marrow or blood cytogenetic samples 47
8.2.2 Physical examinations 47
8.2.3 Vital signs 47
8.2.4 Electrocardiograms 47
8.2.5 Other safety assessments 48
8.2.5.1 Serum or urine pregnancy test 48
8.3 Collection of adverse events 48
8.3.1 Method of detecting AEs and SAEs 49
8.3.2 Time period and frequency for collecting AE and SAE information 49
8.3.2.1 Adverse events after the 30 day follow up period 49
8.3.3 Follow-up of AEs and SAEs 50
8.3.4 Adverse event data collection 50
8.3.5 Causality collection 51
8.3.6 Adverse events based on signs and symptoms 51
8.3.7 Adverse events based on examinations and tests 51
8.3.8 Hy’s law 52
8.3.9 Disease progression 52
8.3.10 New Cancers 52
8.3.11 Lack of efficacy 52
8.3.12 Deaths 53
8.3.13 Olaparib adverse events of special interest 53
8.4 Safety reporting and medical management 53

APPROVED BY THE YALE UNIVERSITY IRB 2/12/2020

APPROVED BY THE YALE UNIVERSITY IRB 2/12/2020

8.4.1 Reporting of serious adverse events....53
8.4.2 Pregnancy....54
8.4.2.1 Maternal exposure....54
8.4.2.2 Paternal exposure....55
8.4.3 Overdose....55
8.4.4 Medication error....55
8.4.5 Management of adverse events related to Olaparib and TMZ....56
8.4.5.1 Management of haematological toxicity....56
Management of anaemia....56
Management of neutropenia, leukopenia and thrombocytopenia....57
Management of prolonged haematological toxicities while on study treatment....58
8.4.5.2 Management of non-haematological toxicity....58
Management of new or worsening pulmonary symptom....59
Management of nausea and vomiting....59
Interruptions for intercurrent non-toxicity related events....59
8.4.5.3 Renal impairment....60
8.5 Pharmacokinetics....61
8.6 Pharmacodynamics....61
8.7 Genetics....61
8.7.1 Collection of optional genetic samples....61
8.7.2 Storage and destruction of genetic samples....61
8.8 Biomarkers....61
8.8.1 Storage, re-use and destruction of biomarker samples....63
9. STATISTICAL CONSIDERATIONS....63
9.1 Statistical hypotheses....63
9.2 Sample size determination....63
9.3 Populations for analyses....64
9.4 Statistical analyses....65
9.4.1 Efficacy analyses....65
9.4.2 Safety analyses....66
9.4.3 Other analyses....66
9.4.4 Methods for multiplicity control....66
9.5 Interim analyses....66
9.5.1 Data monitoring committee (DMC)....66
9.5.2 Safety Monitoring....67
10. REFERENCES....69
11. SUPPORTING DOCUMENTATION AND OPERATIONAL CONSIDERATIONS....80

APPROVED BY THE YALE UNIVERSITY IRB 2/12/2020

APPROVED BY THE YALE UNIVERSITY IRB 2/12/2020

LIST OF TABLES

Table 1 Schedule of Activities....9
Table 2 Objectives and endpoints....16
Table 3 Study Treatments....34
Table 4 Prohibited medications....37
Table 5 Restricted concomitant medications....37
Table 6 Dose reductions for olaparib to manage adverse events....40
Table 7 Dose reductions for TMZ to manage adverse events....41
Table 8 Dose reduction for olaparib if patient develops moderate renal impairment....41
Table 9 Dose reductions for olaparib if patient has to start taking a strong or moderate CYP3A inhibitor....41
Table 10 Laboratory safety variables....46
Table 11 Management of anaemia....56
Table 12 Management of neutropenia, leukopenia and thrombocytopenia....57
Table 13 Dose reductions for study treatment....60
Table 14 Dose reductions for study treatment....60

LIST OF FIGURES

Figure 1 Study design....18
Figure 2 Overview of three key alkylation damage repair pathways....21

LIST OF APPENDICES

LIST OF APPENDICES

Appendix A Actions required in cases of increases in liver biochemistry and evaluation of Hy's Law....81
Appendix B Adverse event definitions and additional safety information....86
Appendix C Acceptable Birth Control Methods....91
Appendix D ECOG Performance Status....93

APPROVED BY THE YALE UNIVERSITY IRB 2/12/2020

APPROVED BY THE YALE UNIVERSITY IRB 2/12/2020

1. PROTOCOL SUMMARY

1.1 Schedule of Activities (SoA)

The procedures for the screening and treatment periods in this study are presented in Table 1.

For olaparib treatment

- Patients may delay dosing under certain circumstances
  - Dosing may be delayed per the Dosing Modification and Toxicity Management Guidelines (see section 8.4.5) due to adverse events (AE)
  - If dosing must be delayed for reasons other than treatment-related toxicity, dosing will resume as soon as feasible.
  - In the event that olaparib is discontinued or delayed as part of the toxicity management guidance, TMZ may still be administered as scheduled.

For Temozolomide (TMZ) treatment

- Dosing may be delayed per the Dosing Modification and Toxicity Management Guidelines (see section 8.4.5) due to AEs.
  - If dosing must be delayed for reasons other than treatment-related toxicity, dosing will resume as soon as feasible.
  - In the event that TMZ is discontinued or delayed as part of the toxicity management guidance, olaparib may still be administered as scheduled.

APPROVED BY THE YALE UNIVERSITY IRB 2/12/2020

Table 1 Schedule of Activities

Tests	Screening	Treatment Period (Each cycle repeats every 21 days)											End of Treatment[a]	Safety Follow -up[m]	Long Term Follow-up[b]
		Cycle 1			Cycle 2		Cycle 3		Cycle 4		Cycle 5 and beyond				
	Day -28 to -1	D1	D8	D15	D1	D 15	D1	D15	D1	D15	D1	D15			
**Administrative Procedures**															
Informed Consent	X														
Inclusion / Exclusion Criteria	X														
Demographics and Medical History	X														
Prior and Concomitant Medication Review	X	X		X	X	X	X	X	X	X	X	X	X	X	
**Clinical Procedures / Assessments**															

APPROVED BY THE YALE UNIVERSITY IRB 2/12/2020

Review Adverse Events	X	X	X	X	X	X	X	X	X	X	X	X	X	X	X
Full Physical Examination	X	X											X		
Directed Physical Examination			X	X	X	X	X	X	X	X	X	X		X	
Vital Signs and Weight[c]	X	X	X	X	X	X	X	X	X	X	X	X	X	X	
ECOG PS[d]	X	X	X	X	X	X	X	X	X	X	X	X	X	X	
Post Study Anti-cancer Therapy Status													X	X	X
Adverse Event Recording		X											X	X	
Long Term Follow-Up															X
SAE Recording		X											X	X	X
**Treatment / Supportive Care Administration**															
Olaparib		Continuous administration[e]													
Temozolomide		X[e]			X[e]		X[e]		X[e]		X[e]				

APPROVED BY THE YALE UNIVERSITY IRB 2/12/2020

**Laboratory Procedures / Assessments**															
Pregnancy Test	X[f]	X[f]			X[f]		X[f]		X[f]		X[f]				
Clinical chemistry[o]	X	X[g]	X[g]	X[g]	X[g]		X[g]		X[g]		X[g]		X		
CBC with differential	X	X[g]	X[g]	X[g]	X[g]		X[g]		X[g]		X[g]		X		
PT/INR and aPTT[p]	X														
Urinalysis	X	X													
TSH	X[h]								X[m]						
EKG	X[n]														
**Efficacy Measurement / Imaging**															
CT Chest / Abdomen / Pelvis	X[i]	Response assessment imaging will be performed every 6 weeks (+/3 days)[j]											X[k]		X[k]
**Tumor Biopsies/Correlative Studies Blood**															
Biopsy[l]	X												X		
Research Blood collection	X	X	X	X	X		X		X				X	X	

APPROVED BY THE YALE UNIVERSITY IRB 2/12/2020

Stool Sample	X[q]						X[q]								

a. An end of treatment visit will be done within 7 days after progression of disease or discontinuation of study treatment for other reasons, and an additional follow up visit at 30 ± 7 days post discontinuation. In the case that the patient initiates a subsequent anti-cancer therapy, the 30 day follow up should occur prior to the first dose of the new therapy and the patient will move to long term follow up.

b. After the end of treatment visits the subjects will enter long-term follow up during which information on survival and subsequent anticancer therapy will be obtained by phone every 12 weeks until the subject dies, is lost to follow up, or withdraws consent. Patients who come off study for reasons other than disease progression should be assessed in the clinic and radiographically every 6 weeks (± 7 days) for the first year and 12 weeks (± 7 days) after the first year.

c. Vital signs should include temperature, pulse, respiratory rate, weight and blood pressure. Height will be measured on day 1 only. For cycles 2 and beyond, vital signs may be performed up to 2 days before Days 1 and/or 15.

d. For treatment cycles 2 and beyond, physical examination, ECOG performance status, weight/BSA may be performed up to 2 days before Days 1 and/or 15.

e. Olaparib will be taken continuously at a starting dose of 150 mg twice daily on days 1-21; Temozolomide will be taken days 1-7 every 21 days at a starting dose of 75 mg/m^2.

f. A pregnancy test at screening and on Day 1 is only required for women of "child bearing potential" as defined in the inclusion criteria. If required, the pregnancy test prior to cycle 1 may be done up to 72 hours prior to the treatment of study medications. A pregnancy test is required on day 1 for each cycle (+/- 72 hours) for women of "child bearing potential."

g. For cycle 1 pre-treatment labs should be drawn within 24 hours prior to study treatment. After cycle 1, pre-treatment labs can be drawn up to 72 hours pre-dose.

h. TSH (reflex T3, FT4) will be drawn at screening and prior to treatment with cycle 4, and every 4 cycles thereafter (i.e. cycle 8, cycle 12, cycle 16, etc…)

i. A baseline CT scan will be done – 28 to -1 days prior to study treatment.

j. Response assessment CT scans will be performed at the end of cycle 2 and at the end of every subsequent second treatment cycle at day 18 +/- 3 days (i.e. Days 15 to 21 of cycles 2, 4, 6, 8 and beyond). After 12 cycles, if disease is stable or improving, imaging will be performed in the same manner after every third cycle.

k. Patients who come off study for reasons other than disease progression will continue to undergo a CT Chest / Abdomen / Pelvis every 6 weeks (± 7 days) for the first year and every 12 weeks (± 7 days) after 1 year.

APPROVED BY THE YALE UNIVERSITY IRB 2/12/2020

l. A mandatory pre-treatment biopsy will be required for all patients if deemed to be low risk and safe in the opinion of the treating investigator. An optional treatment biopsy after disease progression will also be obtained.

m. A safety follow up visit will be scheduled 30 days ± 7 days post discontinuation. If the patient is to start a new therapeutic agent prior to this 30-day period, the safety follow up visit must take place before the subsequent agent is given. Follow up visits will then be every 8 weeks post discontinuation.

n. An EKG will be done at screening and as clinically indicated while on study. If abnormalities on EKG are noted an echocardiogram maybe performed as clinically indicated.

o. Refer to section 8.2.1 for specific laboratory variables to be drawn.

p. Patients who are taking warfarin may participate in this trial; however, it is recommended that INR be monitored carefully at least once per week for the first month, then monthly if the INR is stable.

q. Refer to the lab manual for stool collection instructions.

APPROVED BY THE YALE UNIVERSITY IRB 2/12/2020

APPROVED BY THE YALE UNIVERSITY IRB 2/12/2020

1.2 Synopsis

Principal Investigator

Michael Cecchini
Yale Cancer Center
Yale School of Medicine
333 Cedar Street
New Haven CT 06510
USA

Statistician

Daniel Zelterman
Yale School of Public Health
60 College St
New Haven CT 06520
PO Box 208034
Office: (203) 785-5574
Fax (203) 785-6912
Daniel.zelterman@yale.edu

Protocol Title: Temozolomide and Olaparib for O**^{6}**-Methyguanine DNA Methyltransferase Promoter Hypermethylated Colorectal Cancer

Short Title: TMZ + Olaparib for MGMT Hypermethylated Colorectal Cancer

Rationale:

Colon cancer is the third most common cancer in the United States, and it accounts for 9% of all cancer related deaths (Cronin et al. 2018). Systemic medical therapy is essential in management of these patients. Over the last two decades a number of available systemic treatments have improved both quality of life and overall survival, which now exceeds 2 years for stage IV disease (Loupakis et al. 2014a). However, the optimal sequence for the use of the available drugs is not clear and exposure of all of the active agents seem to be more important than the order of administration. Hence, the more lines of therapy we can offer to patients, the more benefit patients can derive in terms of their survival and quality of life. Effective biomarkers are lacking for CRC.

However, O^{6}-methylguanine DNA methyltransferase (MGMT) promoter hypermethylation is described in up to 40% of colorectal adenocarcinoma in prospective studies as well as subsets of gastric, pancreatic, and neuroendocrine tumors where it has been associated with increased sensitivity to TMZ and dacarbazine (Thomas 2017, Bae 2002, Konduri 2009, Esteller 1999, Kulke 2009, Walter 2015). Promoter hypermethylation of MGMT is most widely described in glioma and when companied by CIMP, almost universally results in MGMT promoter methylation in these tumors (van den Bent 2013, Clark 2016, Noushmehr 2010, Turcan 2012). Promoter hypermethylation ultimately causes decreased MGMT expression and renders these

APPROVED BY THE YALE UNIVERSITY IRB 2/12/2020

APPROVED BY THE YALE UNIVERSITY IRB 2/12/2020

tumors more susceptible to TMZ (Chinot 2007, Brandes 2006, Hegi 2005). The monofunctional alkylator, TMZ causes three common types of alkylation DNA damage, which are depicted in Fig 2. The MGMT protein is the major pathway for the removal of 06-methylguanine lesions, which are extremely toxic in replicating cells and is frequently silenced by promoter hypermethylation in many cancers, either focally or as a result of CIMP. Additionally, a major mechanism for repair of N7-methylguanine and N3-methyladenine lesions is by base excision repair, which is blocked by treatment with PARP inhibitors.

Inhibitors of base excision repair and MGMT have been shown to sensitize tumors to TMZ (Delaney 2000, Trivedi 2005, Barvaux 2004, Hermisson 2006, Yan et al. 2007, Luo 2004, Liu et al. 2003). Tumor sensitivity to TMZ is increased in combination with PARP inhibitors in cell lines and xenografts both *in vitro* and *in vivo* (Smith et al. 2015, Tentori et al. 2003, Palma et al. 2009, Gill et al. 2015b, Delaney 2000, Jagtap and Szabó 2005, Tentori et al. 2001a, Tentori et al. 2001b, Calabrese et al. 2004, Curtin et al. 2004). Furthermore, the PARP inhibitor, veliparib, reverses TMZ resistance in multiple xenografts of various tumor types (Palma et al. 2009). As stated above the base excision repair pathway is an important pathway for demethylation, however the increased TMZ sensitivity with PARP inhibitors is likely multifactorial (Jagtap and Szabó 2005, Boulton et al. 1995). For example, stalled replication forks are typically overcome by HR DNA repair, but PARP inhibitors can delay the initiation of this HR-mediated recovery (Cimprich and Cortez 2008, Yang et al. 2004, Lavin 2008). In addition, PARP trapping appears to be critical for improving TMZ sensitivity, which makes olaparib an ideal PARP inhibitor to use in combination with TMZ (Murai et al. 2014b, Pommier, O'Connor and de Bono 2016, Murai et al. 2012a). The combination of TMZ with PARP inhibitors has gained traction with multiple ongoing clinical trials evaluating various combination in multiple diseases (NCT00687765, NCT0215982, NCT01390571, NCT02116777, NCT01858168 and others) (Ohmoto and Yachida 2017) and specifically the TMZ olaparib combination has been deemed safe and worthy of further evaluation in both GBM and small cell lung cancer in various dosing schemes (Farago et al. 2018, Fulton et al. 2017, Halford et al. 2017).

Temozolomide has activity in the relapsed / refractory setting for CRC, likely related to the high prevalence of MGMT promoter hypermethylation in this disease, which is supported by prospective clinical trials. Based on TMZ resistance mechanisms described, preliminary data suggests the addition of PARP inhibitors can lead to a sustained anti-tumor response and appropriate safety data to support this combination is available. Therefore we propose a biomarker driven, open-label clinical trial with TMZ in combination with the poly ADP ribose polymerase (PARP) inhibitor olaparib for MGMT promoter hypermethylated CRC.

Table 2. Objectives and endpoints:

**Primary objectives:**	**Endpoints/variables:**
To determine the efficacy of TMZ in combination with olaparib in subjects with MGMT promoter hypermethylated advanced colorectal cancer by the overall response rate	ORR by RECIST v1.1 criteria

APPROVED BY THE YALE UNIVERSITY IRB 2/12/2020

(ORR)	
**Secondary objectives:**	**Endpoints/variables:**
To determine the safety of TMZ in combination with olaparib	Rate of ≥ grade 3 adverse events by CTCAE v5.0
To estimate the progression free survival (PFS)	PFS
To estimate overall survival (OS)	OS
**Exploratory objectives:**	**Endpoints/variables:**
Characterize NGS profiling, patterns of DNA methylation, gene expression, and develop MGMT expression assays	Association of NGS profiling, patterns of DNA methylation, gene expression, and develop MGMT expression assays with efficacy and clinical parameters.
Correlate molecular features with γH2AX with response	Association of γH2AX with response rate and survival endpoints
Establish organoids for MGMT promoter hypermethylated colorectal cancer	Form a library of colorectal cancer organoids

Overall design:

This is a Phase II, non-randomized, open-label, single-institution study to evaluate temozolomide in combination with olaparib in patients with MGMT promoter hypermethylated advanced colorectal cancer. Potentially eligible patients will be tested for MGMT promoter hypermethylation by methylation specific PCR after signing a pre-screening consent. The study will begin with a safety evaluation period to assess for dose limiting toxicity (DLT) in the first 6 treated patients, and if toxicity is observed a dose de-escalation schema will be considered.

Study period:

Estimated Date of First Patient Enrolled: January 2020

Estimated Date of Last Patient Completed: January 2022

Number of patients:

The study will enroll 30 patients to receive temozolomide + olaparib combination therapy every 3 weeks (q3w). Patients will provide a tumor tissue sample at screening to determine MGMT promoter hypermethylation by methylation-specific polymerase chain reaction (PCR) analysis.

Treatments and treatment duration:

Systemic therapy:

APPROVED BY THE YALE UNIVERSITY IRB 2/12/2020

Temozolomide + Olaparib: Temozolomide (75 mg/m^2 orally on days 1-7 every 3 weeks) + Olaparib (150 mg orally twice daily days 1-21) in a 21-day cycle.

Duration of treatment:

Treatment will continue until disease progression, unacceptable adverse event(s), withdrawal of consent or death.

Progression during treatment:

During the treatment period, patients who have Response Evaluation Criteria in Solid Tumors, version 1.1 (RECIST 1.1) defined radiological progression will proceed to follow-up. Patients with equivocal progression who are clinically stable may continue on treatment phase of the study at the discretion of the patient and Investigator until progression is confirmed, no earlier than 4 weeks after and preferably within 4 to 6 weeks after the prior assessment of progression of disease.

Follow-up of patients post discontinuation of study drug:

Patients who have discontinued study treatment due to toxicity or symptomatic deterioration, clinical progression/recurrence, or who have commenced subsequent anticancer therapy will be followed until RECIST 1.1-defined progression of disease and then at least for an additional 8 weeks with at least 1 additional follow up scan or death (whichever comes first). A repeat optional tumor biopsy will be done at the time of disease progression unless it is deemed clinically unsafe to perform.

Survival:

All patients in the study should be followed up for survival.

Independent data monitoring committee:

The principal investigator and Yale Center for Clinical Investigation (YCCI) are responsible for monitoring the performance of the study. The Yale Cancer Center Data and Safety Monitoring Committee (DSMC) will provide primary oversight of data and safety monitoring. The Yale DSMC will review and monitor compliance, toxicity and deviations from this study. The committee is composed of clinical specialists with experience in oncology and who have no direct relationship with the study. Information that raises any questions about participant safety will be addressed with the Principal Investigator. The DSMC will review this protocol at a minimum of once every six months.

Statistical methods

We anticipate about 20% of those screened will exhibit an appropriate biomarker as well as meet eligibility criteria. To obtain 30 eligible patients, the mean number of patients to be screened is 150. Using the negative binomial distribution as reference, there is a 6% probability more than 190 subjects will need to be screened to attain the 30 eligible patients.

APPROVED BY THE YALE UNIVERSITY IRB 2/12/2020

The primary endpoint is the objective response rate to the combined therapy and will be assessed in a Simon's two-stage optimal design. Published, historical records anticipate a response rate of 5% or lower and we will assume a null hypothesis of 5%. We will reject this null hypothesis in favor of a higher rate if we observe 4 or more responses in the evaluable patient sample of 30. The trial will terminate early if we do not observe 1 or more responses in the first 9 evaluable patients. The probability of 4 or more responses in a sample of 30 is .049, which is the significance level. If the true, underlying response rate is 25% or greater, then this trial has a power of 90%. The expected sample size is 17 under the null hypothesis. There is a 63% chance of stopping early under the null hypothesis.

Secondary endpoints include overall survival, progression-free survival and toxicity. Survival endpoints will be plotted using the Kaplan-Meier, product-limit method and compared across clinical measurements using Cox proportional hazards regression. Time to progression begins at the time of initial treatment and ends at the earliest of withdrawal, diagnosed progression, or death. Toxicity rates will be compared to established rates for these treatments and summarized using means and 95% confidence intervals. Genetic markers will be plotted using heat-maps and statistically compared across responders and nonresponders using the Benjamini-Hochberg correction for multiplicity of p-values.

1.3 Schema

The general study design is summarised in Figure 1.

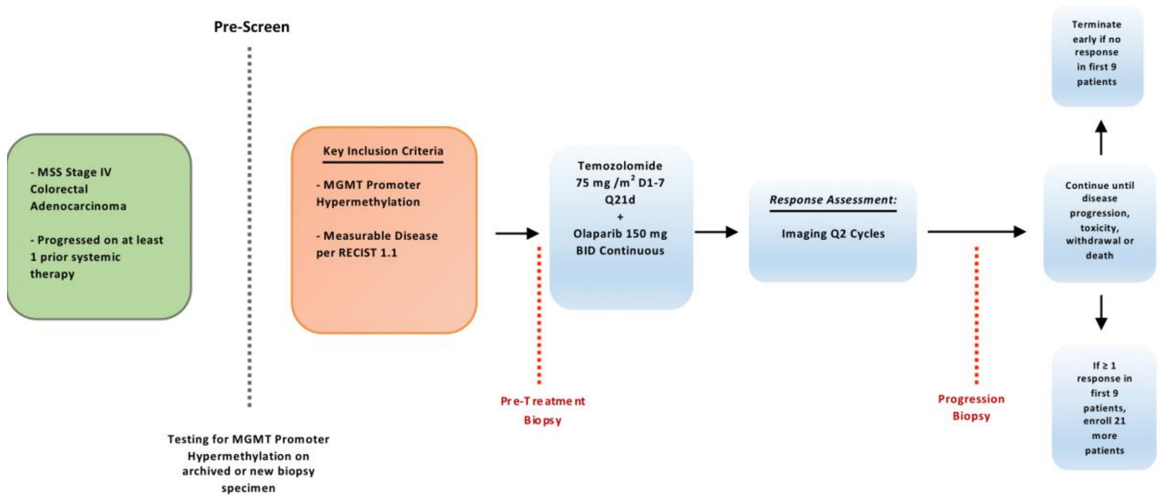

Figure 1 Study design

2. INTRODUCTION

Colon cancer is the third most common cancer in the United States, and it accounts for 9% of all cancer related deaths (Cronin, Lake et al. 2018). More than 100,000 new colon cancer cases are expected to be diagnosed in 2019, with about 20% of these cases having metastatic disease at presentation (Muratore, Zorzi et al. 2007). Unfortunately, only a small number of patients with stage IV disease can be cured with multimodality therapy. Hence, systemic medical therapy is essential in management of these patients. Over the last two decades a number of available systemic treatments for colon cancer have increased significantly. They have improved both quality of life and overall survival, which now exceeds 2 years for stage IV disease (Loupakis, Cremolini et al. 2014).These treatments include conventional chemotherapy agents (fluoropyrimidines, oxaliplatin, and irinotecan), angiogenesis inhibitors (bevacizumab and aflibercept), and anti-EGFR antibodies (cetuximab and panitumumab). Treatments such as regorafenib and lonsurf have had more modest survival benefits when compared to placebo in the third line setting. However, the optimal sequence for the use of the available drugs is not clear. It is the exposure of all of the active agents that seem to be more important than the order of administration. Hence, the more lines of therapy we can offer to patients, the more benefit patients can derive in terms of their survival and quality of life. Development of alternative/novel treatment options is essential for our ability to treat this disease more effectively.

Olaparib (AZD2281, KU-0059436) is a potent Polyadenosine 5'diphosphoribose [poly (ADP ribose)] polymerisation (PARP) inhibitor (PARP-1, -2 and -3) that is being developed as an oral therapy, both as a monotherapy (including maintenance) and for combination with chemotherapy and other anti-cancer agents.

PARP inhibition is a novel approach to targeting tumours with deficiencies in DNA repair mechanisms. PARP enzymes are essential for repairing DNA single strand breaks (SSBs). Inhibiting PARPs leads to the persistence of SSBs, which are then converted to the more serious DNA double strand breaks (DSBs) during the process of DNA replication. During the process of cell division, DSBs can be efficiently repaired in normal cells by homologous recombination repair (HR). Tumours with HR deficiencies (HRD), such as ovarian cancers in patients with BRCA1/2 mutations, cannot accurately repair the DNA damage, which may become lethal to cells as it accumulates. In such tumour types, olaparib may offer a potentially efficacious and less toxic cancer treatment compared with currently available chemotherapy regimens.

BRCA1 and BRCA2 defective tumours are intrinsically sensitive to PARP inhibitors, both in tumour models in vivo (Rottenberg et al 2008, Hay et al 2009) and in the clinic (Fong et al 2009). The mechanism of action for olaparib results from the trapping of inactive PARP onto the single-strand breaks preventing their repair (Helleday 2011; Murai et al 2012). Persistence of SSBs during DNA replication results in their conversion into the more serious DNA DSBs that would normally be repaired by HR repair. Olaparib has been shown to inhibit selected tumour cell lines in vitro and in xenograft and primary explant models as well as in genetic BRCA knock-out models, either as a stand-alone treatment or in combination with established chemotherapies.

APPROVED BY THE YALE UNIVERSITY IRB 2/12/2020

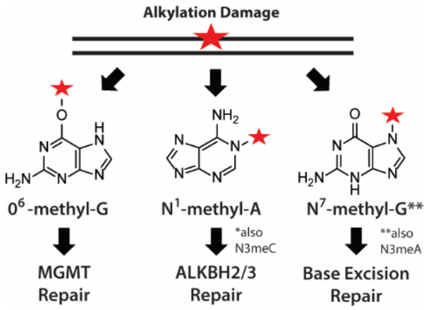

Fig. 2. Overview of three key alkylation damage repair pathways.

Temozolomide (TMZ) is a monofunctional alkylator of DNA approved for the treatment of glioblastoma multiforme (GBM) and neuroendocrine tumors. Aklylation (methylation) of DNA occurs predominantly at the O^6 and N^1 position of guanine. The MGMT protein is critical for the removal of 06-methylguanine lesions, which are extremely toxic in replicating cells. The gene is frequently silenced by promoter hypermethylation in many cancers, either focally or as a result of the CPG island methylator phenotype (CIMP), leading to TMZ sensitivity. Three common types of alkylation DNA damage and their associated DNA repair pathways are shown in Fig. 2, which are briefly reviewed here. The MGMT protein is critical for the removal of 06-methylguanine lesions, which are extremely toxic in replicating cells. This gene is frequently silenced by promoter hypermethylation in many cancers, either focally or as a result of the CPG island methylator phenotype (CIMP), leading to TMZ sensitivity, which is further discussed below. The N7-methylguanine and N3-methyladenine lesions are repaired by base excision repair (BER), which is blocked by treatment with PARP inhibitors.

2.1 Study rationale

2.2 Background

A detailed description of the chemistry, pharmacology, efficacy, and safety of olaparib is provided in the investigator's brochure, and for temozolomide, the package insert.

<u>O^6-Methylguanine DNA Methyltransferase and Cpg Island Methylator Phenotype in Gastrointestinal Malignancies:</u> Dysregulation of DNA repair plays a major role in the initiation and progression for a variety of gastrointestinal (GI) malignancies. This can range from BRCA mutations seen in pancreatic cancer, oncometabolite formation seen in isocitrate dehydrogenase (IDH) mutant cholangiocarcinoma or O^6-methylguanine DNA methyltransferase (MGMT) promoter hypermethylation in colorectal, neuroendocrine, and gastroesophageal cancers. Temozolomide (TMZ) has shown activity in many of these malignancies in the relapsed / refractory setting; however the duration of benefit has been modest. Based on TMZ resistance mechanisms described below, preliminary data suggests the addition of PARP inhibitors can lead to a sustained anti-tumor response. We thus propose a biomarker driven, open-label clinical trial with TMZ in combination with the poly ADP ribose polymerase (PARP) inhibitor olaparib.

Promoter hypermethylation of MGMT is most widely described in glioma where it can be seen together with IDH mutations in grades 2 and 3 glioma, or spontaneously in grade 4. IDH mutant gliomas are accompanied by CIMP, which almost universally results in MGMT promoter methylation in these tumors (Noushmehr 2010, Turcan 2012, van den Bent 2013, Clark 2016). Promoter hypermethylation ultimately causes decreased MGMT expression and renders these tumors more susceptible to TMZ (Hegi 2005, Brandes 2006, Chinot 2007). Both reductions in MGMT expression and MGMT promoter hypermethylation are described in a variety of gastrointestinal malignancies. This includes up to 40% of colorectal adenocarcinoma as well as subsets of gastric, pancreatic, and neuroendocrine tumors where it has been associated with increased sensitivity to TMZ and dacarbazine (Esteller 1999, Bae 2002, Konduri 2009, Kulke

APPROVED BY THE YALE UNIVERSITY IRB 2/12/2020

2009, Walter 2015, Thomas 2017). Additionally, mutations in IDH create the oncometabolite 2-hydroxyglutarate (2HG), which creates CIMP, and ultimately leads to MGMT promoter hypermethylation. In cholangiocarcinoma IDH mutations occur in up to 15-20% of cases (Borger, Tanabe et al. 2012, Javle, Bekaii Saab et al. 2016).

The monofunctional alkylator, TMZ causes three common types of alkylation DNA damage (Fig 1.). The MGMT protein is the major pathway for the removal of 06-methylguanine lesions, which are extremely toxic in replicating cells and is frequently silenced by promoter hypermethylation in many cancers, either focally or as a result of CIMP, which may be IDH1/2 induced, leading to TMZ sensitivity, which is further discussed below. The ALKBH-2 and -3 proteins repair N1-methyladenine and N3-methylcytosine lesions, and they are both inhibited by the oncometabolite 2HG, leading to TMZ sensitivity in this subset (Wang, Wu et al. 2015, Chen, Bian et al. 2017). Finally, N7-methylguanine and N3-methyladenine lesions are repaired by base excision repair, which is blocked by treatment with PARP inhibitors. Collectively, these findings suggest that gastrointestinal tumors with MGMT promoter silencing and/or IDH1/2 mutations will be sensitive to TMZ-based therapeutics.

In colorectal cancer CIMP is well described, which can be caused by a variety of epigenetic changes, including the oncometeabolite 2HG (Figueroa 2010, Noushmehr 2010, Curtin, Slattery et al. 2011, Turcan 2012, Hughes, Melotte et al. 2013, van den Bent 2013). In glioma, CIMP status is associated with a better prognosis (Ceccarelli, Barthel et al. 2016), while oncometabolite-driven CIMP in SDH mutant tumors seems to be associated with a worse prognosis (Letouze, Martinelli et al. 2013). The promoter of MGMT is frequently silenced in CIMP-positive tumors (van den Bent 2013). As discussed below, MGMT promoter hypermethylation predicts for sensitivity to alkylators, such as TMZ, and thus it is an important prognostic factor (Hegi 2005, Brandes 2006, Chinot 2007). The CIMP also drives ARID1A promoter silencing, which encodes a gene important for the replication fork stability. Loss of ARID1A was recently shown to confer sensitivity to both ATR and PARP inhibitors (Shen, Peng et al. 2015, Williamson, Miller et al. 2016), and these mutations are well described in a multitude of cancers including pancreatic cancer (Ryan 2014).

Temozolomide and PARP inhibitors: The combination of TMZ with PARP inhibitors has also gained traction with multiple ongoing clinical trials evaluating various combination in multiple diseases (NCT00687765, NCT0215982, NCT01390571, NCT02116777, NCT01858168 and others) (Ohmoto and Yachida 2017). Inhibitors of base excision repair and MGMT have been shown to sensitize tumors to TMZ (Delaney 2000, Liu, Yan et al. 2003, Barvaux 2004, Luo 2004, Trivedi 2005, Hermisson 2006, Yan, Bulgar et al. 2007). Tumor sensitivity to TMZ is increased in combination with PARP inhibitors in cell lines and xenografts both *in vitro* and *in vivo* (Delaney 2000, Tentori, Portarena et al. 2001, Tentori, Portarena et al. 2001, Tentori, Leonetti et al. 2003, Calabrese, Almassy et al. 2004, Curtin, Wang et al. 2004, Jagtap and Szabó 2005, Palma, Wang et al. 2009, Gill, Travers et al. 2015, Smith, Reynolds et al. 2015). Furthermore, the PARP inhibitor, veliparib, reverses TMZ resistance in multiple xenografts of various tumor types (Palma, Wang et al. 2009). As stated above the base excision repair pathway is an important pathway for demethylation; however, the increased TMZ sensitivity with PARP inhibitors is likely multifactorial (Boulton, Pemberton et al. 1995, Jagtap and Szabó 2005). For example, stalled replication forks are typically overcome by HR DNA repair, but PARP

APPROVED BY THE YALE UNIVERSITY IRB 2/12/2020

inhibitors can delay the initiation of this HR-mediated recovery (Yang, Cortes et al. 2004, Cimprich and Cortez 2008, Lavin 2008). In addition, PARP trapping appears to be critical for improving TMZ sensitivity, which makes olaparib an ideal PARP inhibitor to use in combination with TMZ (Murai, Huang et al. 2012, Murai, Zhang et al. 2014, Pommier, O'Connor et al. 2016).

Multiple dose levels and schedules for olaparib and TMZ have been evaluated in GBM and small cell lung cancer. In small cell carcinoma intermittent dosing (days 1-7) of olaparib 200 mg twice daily and TMZ 75 mg/m^2 every 21 days was reported to be safe with observed clinical activity (Farago, Drapkin et al. 2018). Here we propose continuous dosing of olaparib 150 mg twice daily with TMZ 75 mg/m^2 days, which is further outlined in section 4.3 "Justification for dose."

Temozolomide in Advanced Colorectal Cancer (CRC): Recent phase II studies evaluating MGMT methylated CRC revealed an average response rate of 10% in chemo-refractory disease treated with dacarbazine or TMZ monotherapy (Amatu, Sartore-Bianchi et al. 2013, Hochhauser, Glynne-Jones et al. 2013, Pietrantonio, Perrone et al. 2014, Amatu, Barault et al. 2016, Pietrantonio, de Braud et al. 2016, Calegari, Inno et al. 2017). As noted above, methylation of MGMT and/or decreased MGMT expression is seen in up to 40% of CRC (Esteller 1999, Esteller and Herman 2004, Suehiro, Wong et al. 2008, Shacham-Shmueli, Beny et al. 2011, Thomas 2017, Morano, Corallo et al. 2018). Notably, MGMT promoter hypermethylation is even more common in CRC with KRAS G>A mutations, which may be a result of promoter methylation causing somatic KRAS mutations (Esteller, Toyota et al. 2000, Shacham-Shmueli, Beny et al. 2011). Moreover, a small phase II study evaluated veliparib and TMZ in advanced CRC met its primary endpoint with a 24% disease control rate after 2 cycles (Pishvaian, Slack et al.). Interestingly, PTEN expression and MGMT expression were not predictors of benefit although the sample size was limited. These findings are despite veliparib being poor at PARP trapping, and more significant TMZ sensitization may be seen with other PARP inhibitors such as olaparib (Murai, Huang et al. 2012, Murai, Zhang et al. 2014, Pommier, O'Connor et al. 2016). Temozolomide combinations in CRC continue to be explored, and our collaborators at the Italian National Cancer Institute recently demonstrated in two subsequent single-institutional single arm trials good tolerability and encouraging clinical activity of TMZ in chemorefractory CRC patients with MGMT silencing, even in the RAS-BRAF-mutated population. They also evaluated the prognostic and predictive value of MGMT immunohistochemistry and of MGMT methylation testing both in tumor and cell-free circulating DNA with an ultra-sensitive two-step digital PCR technique (methyl-BEAMing) (Barault 2015, Sartore-Bianchi, Pietrantonio et al. 2017). Their recent investigator initiated clinical trial published May 2018 in *Annals of Oncology* revealed a 24% ORR with the combination of TMZ and irinotecan for refractory MGMT methylated CRC (Morano, Corallo et al. 2018). Notably, all the responders were MGMT negative by IHC and these patients were noted to have a significantly longer PFS.

As noted above, an intact MMR pathway is needed for TMZ sensitivity, and sporadic loss of this pathway has been described as a mechanism of resistance to TMZ (Nagel, Kitange et al. 2017, Thomas, Tanaka et al. 2017). While MMR deficiency may result in resistance to TMZ based therapies, it's been shown to stimulate neoantigen production and confer dramatic sensitivity to anti-PD-1 therapies (Le, Uram et al. 2015, Le, Durham et al. 2017). Thus responding patients may acquire MMR deficiency at progression and an on-progression biopsy for TMZ based therapies can be used to assess for the benefit of immune checkpoint therapy.

APPROVED BY THE YALE UNIVERSITY IRB 2/12/2020

Based on the above rationale we propose to evaluate the safety and efficacy of the TMZ + olaparib combination in colorectal adenocarcinoma with MGMT promoter hypermethylation.

2.3 Benefit/risk assessment

More detailed information about the known and expected benefits and risks and reasonably expected AEs of olaparib may be found in the IB, and for temozolomide in the package insert.

2.3.1 Potential benefits and risks for olaparib

Approximately 15% of ovarian cancer patients and 5% of breast, pancreatic and prostate cancer patients have inherited mutations of BRCA1 or BRCA2. In addition to genetic loss of *BRCA* function, it has been suggested that a further ~20% of tumours display so-called "BRCAness" (Narod and Foulkes 2004; Chan et al 2002). Furthermore, reduced function of other key proteins in the homologous recombination pathway similarly results in increased sensitivity to PARP inhibition and enhancement of chemotherapy and radiotherapy treatments. For these reasons, PARP inhibition represents a novel approach to anti-tumour therapy and may address an unmet need in patients with *BRCA* associated cancer. In addition, the use of PARP inhibitors in combination has confirmed that an enhancement of the anti-tumour activity of radiation and DNA damaging cytotoxic agents occurs (Virag and Szabo 2002, Nguewa et al 2005). Identification of safe and effective doses of olaparib in combination regimens offers potential in many tumour types.

As of 15 December 2018, approximately 10682 patients are estimated to have received olaparib in the clinical programme including AstraZeneca-sponsored studies (6108 patients), a MAP (848 patients), ISSs and collaborative group studies (3726 patients). An estimated 6956 patients with ovarian, breast, pancreatic, gastric and a variety of other solid tumours are estimated to have received treatment with olaparib in AstraZeneca-sponsored, interventional studies (6108 patients) and the MAP (848 patients). Since 2012/2013, most new clinical studies have utilised the tablet formulation, which was designed to deliver the therapeutic dose of olaparib in fewer dose units than the capsule. Of the 6108 patients in AstraZeneca-sponsored, interventional studies, 1512 received the capsule formulation, 4516 received the tablet formulation, and 25 received both capsule and tablet. In the AstraZeneca-sponsored, interventional studies, olaparib was given either as monotherapy (4350 patients) or in combination with chemotherapy or other anti-cancer agents, including studies where patients received monotherapy and combination therapy sequentially (n=1758). Approximately 2071 patients have received comparator or placebo across the olaparib development programme in AstraZeneca-sponsored studies.

Data from the available pre-clinical studies and subsequent clinical development programme demonstrate that olaparib appears to be active and generally well tolerated in patients with solid tumors including those with *BRCA* mutated cancers. In ovarian cancer, responses have been seen in all patient groups, including platinum resistant and refractory cancer.
From the available data to date in patients with advanced cancer, there is no evidence of any unexpected toxicity following long-term olaparib (capsule) monotherapy exposure.
Adverse laboratory findings and/or clinical diagnoses considered to be causally associated with administration of olaparib monotherapy include haematological effects (anaemia, neutropenia, lymphopenia, thrombocytopenia, MCV elevation and increase in blood creatinine), nausea and vomiting, decreased appetite, diarrhea, dyspepsia, stomatitis, upper abdominal pain, dysgeusia,

fatigue (including asthenia), headache, dizziness and cough. Most of these events were generally mild or moderate in intensity.

In a relatively small number of patients, pneumonitis, MDS/AML and new primary malignancies have been observed. Evidence from across the development programme for olaparib does not support a conclusion that there is a causal relationship between olaparib and these events. These are important potential risks for olaparib and are being kept under close surveillance.

Data from studies of olaparib in combination with various chemotherapy agents indicate an increase in bone marrow toxicity (anaemia, neutropenia, thrombocytopenia) greater than expected if the agents had been administered alone. The effects are generally transient but treatment delays are common and alternative administration schedules/toxicity management processes are currently being evaluated within some of these studies. When this type of toxicity has occurred it has been managed by routine clinical practice including dose delays, dose reductions, intermittent dosing and/or the use of supportive care measures, including G-CSF. Currently, all ongoing olaparib combination studies are being closely monitored for myelotoxicity. Results of a Phase II study in combination with carboplatin/paclitaxel (Study D0810C00041) showed that addition of olaparib to carboplatin AUC 4 + paclitaxel in the chemotherapy phase had a generally similar tolerability profile to carboplatin AUC 6 + paclitaxel. The regimen of olaparib capsules 200 mg bd for 10 days out of 21, with carboplatin AUC 4 + paclitaxel had an acceptable and manageable tolerability profile in patients with recurrent serous ovarian cancer. Olaparib tolerability during the monotherapy maintenance phase was consistent with the previously known safety and tolerability profile. In Study D0810C00039 and Study D081BC00004, olaparib 100 mg tablet bd in combination with weekly paclitaxel 80 mg/m2 was well tolerated, with no new unexpected safety findings. In both studies, the incidence of neutropenia was higher for the olaparib combination arm and this contributed to higher rates of dose modifications for patients on olaparib compared to placebo. In Study D081BC00004, there was also a higher incidence of anaemia on the olaparib + paclitaxel combination arm than on the placebo + paclitaxel arm and more dose interruptions, reductions and discontinuations.

2.3.2 Potential benefits and risks for temozolomide

Risks with temozolomide include, but are not limited to, thrombocytopenia, nausea, vomiting, anorexia, constipation alopecia, headache, and constipation.

For more information on all identified and potential risks with temozolomide, please always refer to the package insert.

In adult monotherapy clinical studies, AEs (all grades) reported very commonly (≥10% of patients) are anorexia, fatigue, headache, convulsions, constipation, diarrhea, nausea, vomiting, alopecia, and rash. In a single arm anaplastic astrocytoma trial, hemiparesis, dizziness, abnormal coordination, amnesia, insomnia, and viral infections were also reported very commonly, however, in the absence of a control group it is not clear in many cases whether these reactions should be attributed to temozolomide or the patients' underlying condition.

Temozolomide monotherapy is typically used at a dose of 150-200 mg/m2 on days 1-5 of every 21 or 28 day cycles. It is also used in combination with radiation, and is currently being

investigated in combination with novel agents in multiple prospective clinical trials, including with PARP inhibitors. Temozolomide has been combined with olaparib in multiple clinical trials and has been deemed safe and worthy of further evaluation in both GBM and small cell lung cancer (Fulton, Short et al. 2017, Halford, Cruickshank et al. 2017, Farago, Drapkin et al. 2018). Objective radiographic responses have been observed and multiple dosing schemes are currently being evaluated. Intermittent dosing of olaparib 200 mg twice daily days 1-5 or 1-7 with temozolomide 75 mg/m2 days 1-5 or 1-7 has already been evaluated and deemed safe (Fulton, Short et al. 2017, Farago, Drapkin et al. 2018). Similarly, the OPARATIC trial concluded continuous TMZ 75 mg/m2 and olaparib 150 mg days 1-3 weekly was a safe dose for expansion(Halford, Cruickshank et al. 2017). Given the need for continuous PARP inhibition, PARP inhibition with olaparib 150 mg twice daily in combination with intermittent temozolomide 50-75 mg/m2 is being studied for small cell lung cancer by Farago *et al*, which is further detailed in section 4.3 Justification for dose.

2.3.3 Overall benefit/risk

The treatment options currently available for CRC patients after 5-fluoruracil, oxaliplatin, and irinotecan remain limited. Current standard therapy of TAS-102 or regorafenib is likely to result in modest improvements in long-term survival, and additional and alternative therapies are required for patients with refractory colorectal cancer. Therefore, there remains an unmet medical need for this patient population. The molecular targeting of olaparib to specific subsets of tumors may provide an opportunity for more effective and potentially less toxic cancer treatment for some patients compared with currently available regimens.

3. OBJECTIVES AND ENDPOINTS

Hypothesis: We hypothesize that MGMT promoter hypermethylation will sensitize tumors to the combination of temozolomide with the PARP inhibitor olaparib.

Primary Objectives:

- To determine the efficacy of TMZ in combination with olaparib as measured by the overall response rate.

Secondary Objectives:

- To determine the safety of TMZ in combination with olaparib.
- To estimate progression free survival (PFS).
- To estimate overall survival (OS).

Exploratory Objectives:

APPROVED BY THE YALE UNIVERSITY IRB 2/12/2020

- Identify patterns of next generation sequencing analysis, DNA methylation, mutational load and gene expression in MGMT promoter hypermethylated colorectal cancer.
- Correlate molecular features with markers of DNA damage including γH2AX phosphorylation levels.
- Establish organoids for MGMT promoter hypermethylated colorectal cancer.

Primary Endpoints:

- Objective response rate by RECIST v1.1 criteria for combined therapy with temozolomide and olaparib.

Secondary Endpoints:

- Progression free survival.
- Overall survival.
- Rate of ≥ grade 3 adverse events.

Exploratory Endpoints:

- Association of NGS profiling, patterns of DNA methylation, gene expression, and develop MGMT expression assays with efficacy and clinical parameters.
- Association of γH2AX with response rate and survival endpoints.
- Form a library of colorectal cancer organoids

4. STUDY DESIGN

4.1 Overall design

This is an open-label single arm study of olaparib + temozolomide for MGMT promoter hypermethylated CRC. Potentially eligible patients will be tested for MGMT promoter hypermethylation by methylation specific PCR after signing a pre-screening consent. If patients screen positive for MGMT promoter hypermethylation they will potentially be eligible for enrollment. The study will begin with a safety evaluation period as outlined in section 4.2 to assess tolerability of olaparib and TMZ in the study population. Throughout the study patients will continue with olaparib and temozolomide until objective disease progression (determined by RECIST v1.1) as long as in the investigator's opinion they are benefiting from treatment and they do not meet any other discontinuation criteria.

Once patients have been discontinued from study treatment, other treatment options will be at the discretion of the investigator.

For an overview of the study design see Figure 1, Section 1.3. For details on treatments given during the study, see Section 6.1 Treatments Administered.

APPROVED BY THE YALE UNIVERSITY IRB 2/12/2020

APPROVED BY THE YALE UNIVERSITY IRB 2/12/2020

For details on what is included in the efficacy and safety endpoints, see Section 3 Objectives and Endpoints.

4.2 Safety Evaluation Period

A safety evaluation period to assess for dose limiting toxicity (DLT) will be employed for at least the first 6 treated patients. Should excessive toxicity be observed at the starting dose, a dose de-escalation schema will be used for TMZ to 60 mg/m^2, while the olaparib will be maintained at the specified dose of 150 mg twice daily. Patients will be enrolled in cohorts of 3 during the safety evaluation period. For the first 3 patients treated on the study with olaparib 150 mg twice daily and TMZ 75 mg/m^2, if ≤1/3 DLTs are observed, 3 additional patients will be enrolled at this dose and if ≤1/6 DLTs are seen enrolment will proceed at this dose. If 2 or more DLTs are observed in either the first 3 or 6 patients treated at the starting doses, the TMZ dose will be decreased to 60 mg/m^2 with olaparib 150 mg twice daily. If 2 or more DLTS are seen in either the first 3 or 6 patients treated at TMZ 60 mg/m^2 with olaparib 150 mg twice daily then a further dose reduction to TMZ 50 mg/m^2 with olaparib 150 mg twice daily will be done. The doses of TMZ 50 mg/m^2 D1-7 with olaparib 150 mg twice daily have previously been tested as outlined in section 4.5. Therefore if the starting doses require reduction to TMZ 50 mg/m^2 D1-7 with olaparib 150 mg twice daily then safety will be assessed per the data safety monitoring plan as outlined in section 9.5.2.

The dose management tables throughout the protocol will be adjusted in an amendment as necessary if an alternate starting dose is identified. If a dose reduction is necessary, at least 9 patients (per early stopping rule) and up to 30 evaluable patients will be treated at the reduced dose as part of the efficacy analysis. Therefore in the case of a dose reduction the enrolment of 30 patients will be maintained at the same dose.

4.3 Dose Limiting Toxicity Definition

Dose limiting toxicities will be determined on events that occur during the first 21-day cycle for patients in the safety evaluation period.

Any adverse event of Grade 3 or higher that is at least "possibly related" to olaparib or TMZ will be considered a DLT, unless there is a clear alternative explanation as to the cause of the AE (e.g., disease related), as well as the following conditions:

- Delays of > 7 consecutive days that occurs as a result of TMZ or olaparib.
- Grade 3 mucositis, nausea, vomiting, or diarrhea, AST/ALT elevation will only be considered a DLT if it lasts more than 72 hours despite optimal supportive care.
- Grade 4 mucositis, nausea, vomiting, and diarrhea will be considered DLTs if they reach grade 4 severity despite optimal supportive care, irrespective of duration.
- Any grade 4 hematologic toxicity with the following exceptions:
    - Grade 4 neutropenia will only be considered a DLT if it lasts ≥ 7 days

APPROVED BY THE YALE UNIVERSITY IRB 2/12/2020

APPROVED BY THE YALE UNIVERSITY IRB 2/12/2020

- Any febrile neutropenia will be considered a DLT.
- Any adverse event that meets the definition of Hy's Law.
- Hypersensitivity/Infusion reactions will not be considered a DLT (hypersensitivity reactions are generally not related to the dose level of the drug since they can occur even

Subjects who do not complete both 75% of the TMZ and 75% of the olaparib doses in the 21-day DLT evaluation period, due to reasons other than occurrence of DLT(s) will be considered non-evaluable for DLT, and may be replaced to ensure the accrual of the minimum number of subjects to adequately assess tolerability as outlined in the safety evaluation period.

4.4 Scientific rationale for study design

4.5 Justification for dose

The approved monotherapy dose of olaparib is 300 mg twice daily for the tablet formulation (Olaparib IB). However, when used in combination with additional systemic therapies the olaparib dose is often reduced (Olaparib IB). Temozolomide has numerous established dosage regimens pursuant to whether or not it is used in combination with additional systemic agents or radiotherapy (Refer to TMZ package insert). The combination of olaparib and TMZ has been studied in cell line and pre-clinical rodent models. Combination studies in rats suggested potential for olaparib to exacerbate the effects of TMZ, or topotecan, although combination for olaparib with these anticancer agents did not induce any additional target organ toxicities to those seen with single agent administration (Olaparib IB). Furthermore, the olaparib and TMZ combination is actively being investigated in humans in combination with TMZ in both small cell lung cancer and GBM. In GBM PARADIGM-2 is investigating various dose levels up to olaparib 150 mg twice daily (Days 1-5) and TMZ 75 mg/m^2 daily (continuous) with concomitant radiotherapy (Fulton, Short et al. 2017). Also in GBM the OPARATIC trial concluded continuous TMZ 75 mg/m2 and olaparib 150 mg days 1-3 weekly was a safe dose for expansion (Halford, Cruickshank et al. 2017). This further supports the safety of continuous dosing. While in small cell lung cancer a 3+3 dose escalation identified olaparib 200 mg twice daily (Days 1-7) and TMZ 75 mg/m^2 (Days 1-7) as the recommend phase 2 dose of the combination (Farago, Drapkin et al. 2018). However, to achieve continuous PARP inhibition and PARP trapping, olaparib is being investigated with continuous dosing is being tested by Farago *et al*. At present doses of olaparib 150 mg twice daily (continuous) with TMZ 50-75 mg/m^2 daily (D1-7) are tolerable with no unanticipated toxicities although published data is unavailable at this time. Therefore, we will proceed with 21 day cycles of olaparib 150 mg twice daily (continuous – days 1-21) + TMZ 75 mg/m^2 (days 1-7) with a dose de-escalation schema if unacceptable toxicity is observed in the first 6 patients.

4.6 End of study definition

The end of study is defined as the last expected visit/contact of the last patient undergoing the study.

APPROVED BY THE YALE UNIVERSITY IRB 2/12/2020

APPROVED BY THE YALE UNIVERSITY IRB 2/12/2020

5. STUDY POPULATION

Prospective approval of protocol deviations to recruitment and enrolment criteria, also known as protocol waivers or exemptions, is not permitted.

Each patient should meet all of the inclusion criteria and none of the exclusion criteria for this study in order to assigned to a study intervention. Under no circumstances can there be exceptions to this rule. Patients who do not meet the entry requirements are screen failures, refer to section 5.4.

5.1 Inclusion criteria

Patients are eligible to be included in the study only if all the following inclusion criteria and none of the exclusion criteria apply:

Informed consent

1. Capable of giving signed informed consent which includes compliance with the requirements and restrictions listed in the informed consent form (ICF) and in this protocol.

2. Provision of signed and dated, written informed consent form prior to any mandatory study specific procedures, sampling, and analyses.

3. For inclusion in i) the optional exploratory genetic research and ii) the optional biomarker research, patients must fulfil the following criteria:

- Provision of informed consent for genetic research prior to collection of sample
- Provision of informed consent for biomarker research prior to collection of sample

If a patient declines to participate in the optional exploratory genetic research or the optional biomarker research, there will be no penalty or loss of benefit to the patient. The patient will not be excluded from other aspects of the study.

Age

4. Subject must be at least 18 years of age inclusive, at the time of signing the informed consent form.

Type of patient and disease characteristics

5. Individuals who are healthy as determined by medical evaluation including medical history, physical examination, laboratory tests, and cardiac monitoring.

6. Individuals with histologically proven relapsed/refractory mismatch repair proficient / microsatellite stable metastatic colorectal adenocarcinoma.

7. MGMT promoter hypermethylation on pre-screening.

APPROVED BY THE YALE UNIVERSITY IRB 2/12/2020

APPROVED BY THE YALE UNIVERSITY IRB 2/12/2020

8. Patients must have had recurrence, progression or intolerance to standard therapy consisting of at least 2 prior standard regimens (containing a fluropyrimidine plus a platinum analogue and/or irinotecan) for metastatic disease. In the case where all chemotherapy agents are used concurrently (I.e. FOLFOXIRI +/- Bevacizumab) 1 prior treatment regimen is acceptable after discussion with the principal investigator. Relapse within 6 months of completing adjuvant chemotherapy is considered one line of therapy in the metastatic setting.

9. Patients must have normal organ and bone marrow function measured within 28 days prior to administration of study treatment as defined below:

- Haemoglobin ≥ 10.0 g/dL with no blood transfusion in the past 28 days
- Absolute neutrophil count (ANC) ≥ 1.5 x 10^9/L
- Platelet count ≥ 100 x 10^9/L
- Total bilirubin ≤ 1.5 x institutional upper limit of normal (ULN)
- Aspartate aminotransferase (AST) (Serum Glutamic Oxaloacetic Transaminase (SGOT)) / Alanine aminotransferase (ALT) (Serum Glutamic Pyruvate Transaminase (SGPT)) ≤ 2.5 x institutional upper limit of normal unless liver metastases are present in which case they must be ≤ 5x ULN
- Patients must have creatinine clearance estimated of ≥51 mL/min using the Cockcroft-Gault equation or based on a 24 hour urine test :

$$\text{Estimated creatinine clearance} = \frac{\text{(140-age [years]) x weight (kg)}}{\text{serum creatinine (mg/dL) x 72}} \quad (\text{x F})^{a}$$

[a] where F=0.85 for females and F=1 for males.

10. Eastern Cooperative Oncology Group (ECOG) performance status 0-1 (see Appendix D).

11. Patients must have a life expectancy ≥ 16 weeks.

12. At least one lesion, not previously irradiated, that can be accurately measured at baseline as ≥ 10 mm in the longest diameter (except lymph nodes which must have short axis ≥ 15 mm) with computed tomography (CT) or magnetic resonance imaging (MRI) and which is suitable for accurate repeated measurements.

Reproduction

13. Postmenopausal or evidence of non-childbearing status for women of childbearing potential: negative urine or serum pregnancy test within 28 days of study treatment and confirmed prior to treatment on day 1.

APPROVED BY THE YALE UNIVERSITY IRB 2/12/2020

APPROVED BY THE YALE UNIVERSITY IRB 2/12/2020

Postmenopausal is defined as:

- Amenorrheic for 1 year or more following cessation of exogenous hormonal treatments
- Luteinizing hormone (LH) and Follicle stimulating hormone (FSH) levels in the post menopausal range for women under 50
- radiation-induced oophorectomy with last menses >1 year ago
- chemotherapy-induced menopause with >1 year interval since last menses
- surgical sterilisation (bilateral oophorectomy or hysterectomy)

14. Male patients must use a condom during treatment and for 3 months after the last dose of olaparib when having sexual intercourse with a pregnant woman or with a woman of childbearing potential. Female partners of male patients should also use a highly effective form of contraception ([see appendix C for acceptable methods]) if they are of childbearing potential

5.2 Exclusion criteria

Medical conditions

1. Other malignancy unless curatively treated with no evidence of disease for ≥5 years except: adequately treated non-melanoma skin cancer, curatively treated in situ cancer of the cervix, ductal carcinoma in situ (DCIS), Stage 1, grade 1 endometrial carcinoma.

2. Resting ECG indicating uncontrolled, potentially reversible cardiac conditions, as judged by the investigator (eg., unstable ischemia, uncontrolled symptomatic arrhythmia, congestive heart failure, QTcF prolongation >500 ms, electrolyte disturbances, etc.), or patients with congenital long QT syndrome.

3. Persistent toxicities (Common Terminology Criteria for Adverse Event (CTCAE) grade 2) caused by previous cancer therapy, excluding alopecia.

4. Patients with myelodysplastic syndrome/acute myeloid leukaemia or with features suggestive of MDS/AML.

5. Patients with symptomatic uncontrolled brain metastases. A scan to confirm the absence of brain metastases is not required. The patient can receive a stable dose of corticosteroids before and during the study as long as these were started at least 4 weeks prior to treatment. Patients with spinal cord compression unless considered to

APPROVED BY THE YALE UNIVERSITY IRB 2/12/2020

have received definitive treatment for this and evidence of clinically stable disease for 28 days.

6. Patients considered a poor medical risk due to a serious, uncontrolled medical disorder, non-malignant systemic disease or active, uncontrolled infection. Examples include, but are not limited to, uncontrolled ventricular arrhythmia, recent (within 3 months) myocardial infarction, uncontrolled major seizure disorder, unstable spinal cord compression, superior vena cava syndrome, extensive interstitial bilateral lung disease on High Resolution Computed Tomography (HRCT) scan or any psychiatric disorder that prohibits obtaining informed consent.

7. Patients unable to swallow orally administered medication and patients with gastrointestinal disorders likely to interfere with absorption of the study medication.

8. Immunocompromised patients, e.g., patients who are known to be serologically positive for human immunodeficiency virus (HIV).

9. Patients with known active hepatitis (i.e. Hepatitis B or C).

- Active hepatitis B virus (HBV) is defined by a known positive HBV surface antigen (HBsAg) result. Patients with a past or resolved HBV infection (defined as the presence of hepatitis B core antibody and absence of HBsAg) are eligible.
- Patients positive for hepatitis C virus (HCV) antibody are eligible only if polymerase chain reaction is negative for HCV RNA.

Prior/concomitant therapy

10. Any previous treatment with PARP inhibitor, including Olaparib.

11. Any previous treatment with temozolomide or other monofunctional alkylating agent.

12. Patients receiving any systemic chemotherapy or radiotherapy (except for palliative reasons) within 3 weeks prior to study treatment

13. Concomitant use of known strong CYP3A inhibitors (eg. itraconazole, telithromycin, clarithromycin, protease inhibitors boosted with ritonavir or cobicistat, indinavir, saquinavir, nelfinavir, boceprevir, telaprevir) or moderate CYP3A inhibitors (eg. ciprofloxacin, erythromycin, diltiazem, fluconazole, verapamil). The required washout period prior to starting olaparib and temozolomide is 2 weeks.

14. Concomitant use of known strong (eg. phenobarbital, enzalutamide, phenytoin, rifampicin, rifabutin, rifapentine, carbamazepine, nevirapine and St John's Wort) or moderate CYP3A inducers (eg. bosentan, efavirenz, modafinil). The required washout period prior to starting olaparib and temozolomide is 5 weeks for enzalutamide or phenobarbital and 3 weeks for other agents.

15. Major surgery within 2 weeks of starting study treatment and patients must have recovered from any effects of any major surgery.

16. Previous allogenic bone marrow transplant or double umbilical cord blood transplantation (dUCBT).

17. Whole blood transfusions in the last 120 days prior to entry to the study (packed red blood cells and platelet transfusions are acceptable, for timing refer to inclusion criteria no.8).

Prior/concurrent clinical study experience

18. Participation in another clinical study with an investigational product administered in the last 28 days.

19. Subjects with a known hypersensitivity to temozolomide, olaparib, any of the excipients of either product, or the combination.

20. Patients with a known hypersensitivity to the combination/comparator agent.

Other exclusions

21. Involvement in the planning and/or conduct of the study

22. Judgment by the investigator that the patient should not participate in the study if the patient is unlikely to comply with study procedures, restrictions and requirements.

23. Previous enrolment in the present study.

24. Breast feeding women.

5.3 Lifestyle restrictions

5.3.1 Meals and dietary restrictions

It is prohibited to consume grapefruit juice while on temozolomide and olaparib therapy.

5.3.2 Activity

Contraception

Women of childbearing potential and their partners, who are sexually active, must agree to the use of TWO highly effective forms of contraception in combination (as described in Appendix C). This should be started from the signing of the informed consent and continue throughout the period of taking study treatment and for at least 6 months after last dose of study drug(s), or they must totally/truly abstain from any form of sexual intercourse (as described in Appendix C).

Male patients must use a condom during treatment and for 3 months after the last dose of olaparib when having sexual intercourse with a pregnant woman or with a woman of

childbearing potential. Female partners of male patients should also use a highly effective form of contraception (as described in Appendix C) if they are of childbearing potential. Male patients should not donate sperm throughout the period of taking olaparib and for 3 months following the last dose of olaparib.

For details of acceptable methods of contraception refer to Appendix C Acceptable Birth Control Methods.

5.4 Screen failures

Screen failures are defined as patients who signed the informed consent form to participate in the clinical study but are not subsequently entered in the study due to not meeting eligibility criteria. Patients who decide they no longer want to participate in the study either in screening or before completing 1 cycle of therapy will be considered a withdrawal. Minimal information includes demography, screen failure details, eligibility criteria, and any serious adverse event (SAE).

Individuals who do not meet the criteria for participation in this study (screen failure) may be rescreened up to 2 times.

These patients should have the reason for study withdrawal recorded in the eCRF.

6. STUDY TREATMENTS

Study treatment is defined as any investigational product(s) (including marketed product comparator and placebo) or medical device(s) intended to be administered to a study participant according to the study protocol. Study treatment in this study refers to olaparib and temozolomide. If excessive DLTs are observed at the starting doses for the investigational products, alternate doses are outlined in section 4.2.

6.1 Treatments administered

6.1.1 Investigational products

Table 2 Study Treatments

	**olaparib**	**Temozolomide**
**Study treatment name:**	Olaparib	Temozolomide
**Dosage formulation:**	150 mg twice daily	75 mg/m^2 days 1-7
**Route of administration**	Oral	Oral

**Dosing instructions:**	Olaparib tablets should be taken at the same time each day, approximately 12 hours apart with one glass of water. The tablets should be swallowed whole and not chewed, crushed, dissolved or divided. Olaparib tablets can be taken with or without food.	Temozolomide tablets should be taken at the same time as the AM olaparib dose.
**Packaging and labelling**	Olaparib will be supplied from commercial stock. Study treatment will be provided. Each container will be labelled in accordance with Good Manufacturing Practice (GMP) Annex 13 and per country regulatory requirement.	

If vomiting occurs shortly after the olaparib tablets are swallowed, the dose should only be replaced if all of the intact tablets can be seen and counted. Should any patient enrolled on the study miss a scheduled dose for whatever reason (e.g., as a result of forgetting to take the tablets or vomiting), the patient will be allowed to take the scheduled dose up to a maximum of 2 hours after that scheduled dose time. If greater than 2 hours after the scheduled dose time, the missed dose is not to be taken and the patient should take their allotted dose at the next scheduled time.

6.2 Preparation/handling/storage/accountability

The investigator or designee must confirm appropriate temperature conditions have been maintained during transit for all study treatment received and any discrepancies are reported and resolved before use of the study treatment.

Only patients enrolled in the study may receive study treatment and only authorised site staff may dispense study treatment. At site, all study treatments must be stored in a secure, environmentally controlled, and monitored (manual or automated) area in accordance with the labelled storage conditions with access limited to the investigator and authorised site staff.

The investigator, institution, or the head of the medical institution (where applicable) is responsible for study treatment accountability, reconciliation, and record maintenance (i.e., receipt, reconciliation, and final disposition records).

APPROVED BY THE YALE UNIVERSITY IRB 2/12/2020

Further guidance and information for the final disposition of unused study treatment are provided in the pharmacy manual.

6.3 Measures to minimise bias: randomisation and blinding

This is an open-label single arm clinical trial and therefore there is no randomisation or blinding. If a patient withdraws from the study, then his/her enrolment code cannot be reused. Withdrawn patients will not be replaced provided they have completed at least 1 cycle of therapy.

6.4 Treatment compliance

Patients should be given clear instructions on how and when to take their study treatment. Patients will self-administer olaparib and temozolomide. Study site staff will count tablets at regular intervals during treatment. After the tablet count has been performed, the remaining tablets will not be returned to the patient but will be retained by the investigative site. All patients must return their bottle(s) of olaparib and temozolomide at the appropriate scheduled visit, when a new bottle will be dispensed. Patients will be instructed to notify study site personnel of missed doses.

Any change from the dosing schedule, does interruptions, dose reductions, dose discontinuations should be recorded in eCRF.

The Investigational Product Storage Manager is responsible for managing the IMP from receipt by the study site until the destruction or return of all unused IMP. The Investigator(s) is responsible for ensuring that the patient has returned all unused IMP.

6.5 Concomitant therapy

The use of any natural/herbal products or other traditional remedies should be discouraged, but use of these products, as well as any medication or vaccine including over-the-counter or prescription medicines, vitamins, and/or herbal supplements that the patient is receiving at the time of enrolment or receives during the study must be recorded along with:

- Reason for use
- Dates of administration including start and end dates
- Dosage information including dose and frequency

Anti-emetics/Anti-diarrheals

Should a patient develop nausea, vomiting and / or diarrhea, then these symptoms should be reported as AEs (see section 8.3) and appropriate treatment of the event given.

APPROVED BY THE YALE UNIVERSITY IRB 2/12/2020

Medications that may NOT be administered

Table 4 Prohibited medications

**Prohibited medication/class of drug:**	
Anticancer therapy:<br>Chemotherapy<br>Immunotherapy<br>Hormonal therapy*<br>Radiotherapy (except palliative)<br>Biological therapy<br>Other novel agents	Not permitted while the patient is receiving study medication
Live virus vaccines<br>Live bacterial vaccines	Not permitted while the patient is receiving study medication and during the 30 day follow up period.<br>An increased risk of infection by the administration of live virus and bacterial vaccines has been observed with conventional chemotherapy drugs and the effects with olaparib are unknown.

*Hormone Replacement Therapy (HRT) is acceptable

Restricted concomitant medications

Table 5 Restricted concomitant medications

**Medication/class of drug:**	**Usage (including limits for duration permitted and special situations in which it's allowed):**

APPROVED BY THE YALE UNIVERSITY IRB 2/12/2020

Table 5 Restricted concomitant medications

**Medication/class of drug:**	**Usage (including limits for duration permitted and special situations in which it's allowed):**
Strong CYP3A inhibitors:<br><br>itraconazole, telithromycin, clarithromycin, boosted protease inhibitors, indinavir, saquinavir, nelfinavir, boceprevir, telaprevir<br><br>Moderate CYP3A inhibitors:<br><br>ciprofloxacin, erythromycin, diltiazem, fluconazole, verapamil	Strong or moderate CYP3A inhibitors should not be taken with olaparib. If there is no suitable alternative concomitant medication then the dose of olaparib should be reduced for the period of concomitant administration. The dose reduction of olaparib should be recorded in the CRF with the reason documented as concomitant CYP3A inhibitor use.<br><br>• Strong CYP3A inhibitors – reduce the dose of olaparib to 100 mg daily for the duration of concomitant therapy with the strong inhibitor and for 5 half lives afterwards.<br>• Moderate CYP3A inhibitors – reduce the dose of olaparib to 100 mg twice daily for the duration of concomitant therapy with the moderate inhibitor and for 3 half lives afterwards.<br>• After the washout of the inhibitor is complete, the olaparib dose can be re-escalated.
Strong inducers:<br><br>phenobarbital, phenytoin, rifampicin, rifabutin, rifapentine, carbamazepine, nevirapine, enzalutamide and St John's Wort<br><br>Moderate CYP3A inducers:<br><br>bosentan, efavirenz and modafinil	Strong or moderate CYP3A inducers should not be taken with olaparib.<br><br>If the use of any strong or moderate CYP3A inducers are considered necessary for the patient's safety and welfare this could diminish the clinical efficacy of olaparib.<br><br>If a patient requires use of a strong or moderate CYP3A inducer then they must be monitored carefully for any change in efficacy of olaparib and TMZ.

APPROVED BY THE YALE UNIVERSITY IRB 2/12/2020

Table 5 Restricted concomitant medications

**Medication/class of drug:**	**Usage (including limits for duration permitted and special situations in which it's allowed):**
• CYP3A4 substrates: hormonal contraceptive, simvastatin, cisapride, cyclosporine, ergot alkaloids, fentanyl, pimozide, sirolimus, tacrolimus and quetiapine<br>• CYP2B6 substrates: bupropion, efavirenz<br>• OATP1B1substrates: bosentan, glibenclamide, repaglinide, statins and valsartan<br>• OCT1, MATE1 and MATE2K substrates: metformin<br>• OCT2 substrates: serum creatinine<br>• OAT3 substrates: furosemide, methotrexate	Effect of olaparib on other drugs<br><br>Based on limited *in vitro* data, olaparib may increase the exposure to substrates of CYP3A4, OATP1B1, OCT1, OCT2, OAT3, MATE1 and MATE2K.<br><br>Based on limited *in vitro* data, olaparib may reduce the exposure to substrates of 2B6.<br><br>Caution should be observed if substrates of these isoenzymes or transporter proteins are co-administered.
Anticoagulant therapy	Patients who are taking warfarin may participate in this trial; however, it is recommended that international normalised ratio (INR) be monitored carefully at least once per week for the first month, then monthly if the INR is stable. Subcutaneous heparin and low molecular weight heparin are permitted.
Palliative radiotherapy	Palliative radiotherapy may be used for the treatment of pain at the site of bony metastases that were present at baseline, provided the investigator does not feel that these are indicative of clinical disease progression during the study period. Study treatment should be discontinued for a minimum of 3 days before a patient undergoes therapeutic palliative radiation treatment. Study treatment should be restarted within 4 weeks as long as any bone marrow toxicity has recovered.

Table 5 Restricted concomitant medications

Medication/class of drug:	Usage (including limits for duration permitted and special situations in which it's allowed):
Administration of other anti-cancer agents	Patients must not receive any other concurrent anti-cancer therapy, including investigational agents, while on study treatment. Patients may continue the use of bisphosphonates or denosumab for bone disease and corticosteroids for the symptomatic control of brain metastases provided the dose is stable before and during the study and they were started at least 4 weeks prior to beginning study treatment.

6.5.1 Background medication

6.5.2 Other concomitant treatment

Other medication other than that described above, which is considered necessary for the patient's safety and wellbeing, may be given at the discretion of the Investigator and recorded in the appropriate sections of the Electronic Case Report Form.

In addition, any unplanned diagnostic, therapeutic or surgical procedure performed during the study period must be recorded in the eCRF.

6.6 Dose Modifications

Dose Reductions

In case a dose reduction is necessary, the Study treatments will be administered as follows:

Table 6 Dose reductions for olaparib to manage adverse events

Initial Dose	Following re-challenge post interruption: Dose reduction 1	Dose reduction 2
150 mg twice daily	100 mg twice daily	100 mg once daily

APPROVED BY THE YALE UNIVERSITY IRB 2/12/2020

Table 7 Dose reductions for TMZ to manage adverse events

Initial Dose	Following re-challenge post interruption: Dose reduction 1	Dose reduction 2
75 mg / m^2 (days 1-7)	60 mg / m^2 (days 1-7)	50 mg / m^2 (days 1-7)
*60 mg / m^2 (days 1-7)	50 mg / m^2 (days 1-7)	40 mg / m^2 (days 1-7)
*50 mg / m^2 (days 1-7)	40 mg / m^2 (days 1-7)	30 mg / m^2 (days 1-7)

* If the starting dose of TMZ is lowered as described in section 4.2.

Table 8 Dose reduction for olaparib and TMZ if patient develops moderate renal impairment

Initial Dose	Moderate renal impairment (calculated creatinine clearance by Cockcroft -Gault equation or based on a 24 hour urine test between 31 and 50 ml/min): Dose reduction
Olaparib: 150 mg twice daily	100 mg twice daily
TMZ: 75 mg / m^2 (days 1-7)	For TMZ no dose adjustments are required for moderate renal impairment.

For TMZ no dose adjustments are required for renal impairment.

Table 9 Dose reductions for olaparib and TMZ if patient has to start taking a strong or moderate CYP3A inhibitor

Initial Dose	Strong CYP3A inhibitor	Moderate CYP3A inhibitor
Olaparib: 150 mg twice daily	100 mg daily	100 mg twice daily
TMZ: 75 mg / m^2 (days 1-7)	For TMZ no dose adjustments are required for concomitant CYP3A4 inhibitors.	For TMZ no dose adjustments are required for concomitant CYP3A4 inhibitors.

For TMZ no adjustments are required for concomitant CYP3A inhibitors.

For guidance on dose reductions for management of AEs (including renal impairment) refer to section 8.4.5.

For guidance on dose reductions when concomitant strong or moderate CYP3A inhibitors cannot be avoided see section 6.5.

When dose reduction for olaparib is necessary patients will take one 100 mg tablet twice (see Section 8.4.5 and Section 6.5).

APPROVED BY THE YALE UNIVERSITY IRB 2/12/2020

APPROVED BY THE YALE UNIVERSITY IRB 2/12/2020

7. DISCONTINUATION OF TREATMENT AND PATIENT WITHDRAWAL

7.1 Discontinuation of study treatment

Patients may be discontinued from investigational product (IP) in the following situations. Note that discontinuation from study treatment is NOT the same thing as a complete withdrawal from the study

- Patient decision. The patient is at any time free to discontinue treatment, without prejudice to further treatment
- Adverse Event
- Severe non-compliance with the Clinical Study Protocol
- Bone marrow findings consistent with myelodysplastic syndrome (MDS)/acute myeloid leukaemia (AML)

7.1.1 Procedures for discontinuation of study treatment

The investigator should instruct the patient to contact the site before or at the time if Study treatment is stopped. A patient that decides to discontinue Study treatment will always be asked about the reason(s) and the presence of any AEs. The date of last intake of Study treatment should be documented in the CRF (electronic or paper). All Study treatment should be returned by the patient at their next on-site study visit or unscheduled visit. Patients permanently discontinuing Study treatment should be given locally available standard of care therapy, at the discretion of the Investigator.

Any patient discontinuing investigational product should be seen post discontinuation for the evaluations and sample collections outlined in the study schedule.

After discontinuation of the study medication at any point in the study, all ongoing AEs or SAEs must be followed until resolution unless, in the Investigator's opinion the condition is unlikely to resolve due to the patients underlying disease, or the patient is lost to follow up (see Section 7.2). All new AEs and SAEs occurring during the 30 calendar days after the last dose of study medication must be reported (if SAEs, they must be reported within 24 hours as described in Section 8.4.1) and followed to resolution as above. Patients should be seen at least 30 days after discontinuing study medication to collect and / or complete AE information. For guidance on reporting adverse events after the 30 day follow up period see Section 8.3.2.1.

Discontinuation of Olaparib and TMZ, for any reason, does not impact on the patient´s participation in the study. The patient should continue attending subsequent study visits and data collection should continue according to the study protocol. If the patient does not agree to continue in-person study visits, a modified follow-up must be arranged to ensure the collection of survival endpoints. This could be a telephone contact with the patient every 3 months a contact with a relative or treating physician, or information from medical records. The approach

APPROVED BY THE YALE UNIVERSITY IRB 2/12/2020

taken should be recorded in the medical records. A patient that agrees to modified follow-up is not considered to have withdrawn consent or to have withdrawn from the study.

7.2 Lost to follow-up

Patients will be considered lost to follow-up only if no contact has been established after the site reaches out >3 times. Patients who refuse to continue participation in the study, including telephone contact, should be documented as "withdrawal of consent" rather than "lost to follow-up." Investigators should document attempts to re-establish contact with missing patients throughout the study period. If contact with a missing patient is re-established, the patient should not be considered lost to follow-up and evaluations should resume according to the protocol.

In order to support key end points of PFS and OS, the survival status of all patients in the efficacy analysis set (ITT population) and safety analysis set should be re-checked; this includes those patients who withdraw consent or are classified as "lost to follow-up."

- Lost to follow up – site personnel should check hospital records, the patient's current physician, and a publicly available death registry (if available) to obtain a current survival status.
- In the event that the patient has actively withdrawn consent to the processing of their personal data, the survival status of the patient can be obtained by site personnel from publicly available death registries (if available) where it is possible to do so under applicable local laws to obtain a current survival status.

7.3 Withdrawal from the study

Patients are free to withdraw from the study at any time, without prejudice for further treatment.

A patient who withdraws consent will always be asked about the reason(s) and the presence of any AEs. The investigator will follow up AEs outside of the clinical study.

If a patient withdraws from participation in the study, then his/her enrolment code cannot be reused. Withdrawn patients will not be replaced.

If a patient withdraws consent, they will be specifically asked if they are withdrawing consent to:

- Further participation in the study including any further follow up (e.g. survival cals)
- Withdrawal of consent to the use of their study generated data
- Withdrawal of the use of any samples

The status of ongoing, withdrawn (from the study) and "lost to follow-up" patients at the time of an OS analysis should be obtained by the site personnel by checking patient notes, hospital records, contacting the patient's general practitioner and checking publicly available death registries. In the event that the patient has actively withdrawn consent to the processing of their personal data, the vital status of the patient can be obtained by site personnel from publicly available resources where it is possible to do so under applicable local laws.

APPROVED BY THE YALE UNIVERSITY IRB 2/12/2020

8. STUDY ASSESSMENTS AND PROCEDURES

Study procedures and their timing are summarized in the SoA.

The investigator will ensure that the data are recorded on the eCRFs. The web based OnCore system will be used for data collection and handling.

The investigator ensures the accuracy, completeness, legibility, and timeliness of the data recorded and of the provision of answers to data queries according to the Clinical Study Agreement. The investigator will sign the complete electronic CRFs. A copy of the completed electronic CRFs will be archived at the study site.

Immediate safety concerns should be discussed with the overall principal investigator immediately upon occurrence or awareness to determine if the patient should continue or discontinue study treatment.

Adherence to the study design requirements, including those specified in the SoA, is essential and required for study conduct.

All screening evaluations must be completed and reviewed to confirm that potential patients meet all eligibility criteria. The investigator will maintain a screening log to record details of all patients screened and to confirm eligibility or record reasons for screening failure, as applicable.

Procedures conducted as part of the patient’s routine clinical management (e.g. blood count) and obtained before signing of the ICF may be utilized for screening or baseline purposes provided the procedures met the protocol-specified criteria and were performed within the time frame defined in the SoA.

8.1 Efficacy assessments

This study will evaluate the primary endpoint of overall response rate by RECIST v1.1. The overall response rate will be calculated by the sponsor (Yale School of Medicine) as the proportion of patients that receive either a partial response or complete response by RECIST v1.1 (*Eur J Ca* 45: 228-247, 2009).

Response Criteria:

Complete Response: Disappearance of all target lesions. Any pathological lymph nodes (whether target or non-target) must have reduction in short axis to <10 mm (<1 cm).

Partial Response: At least a 30% decrease in the sum of the diameters of target lesions, taking as reference the baseline sum diameters.

Progressive Disease: At least a 20% increase in the sum of the diameters of target lesions, taking as reference the smallest sum on study (this includes the baseline sum if that is the smallest on study). In addition to the relative increase of 20%, the sum must also demonstrate an absolute increase of at least 5 mm (0.5 cm). (Note: the appearance of one or more new lesions is also considered progression).

APPROVED BY THE YALE UNIVERSITY IRB 2/12/2020

Stable Disease: Neither sufficient shrinkage to qualify as partial response nor sufficient increase to qualify for progressive disease, taking as reference the smallest sum diameters while on study.

Progression-free survival, overall survival and safety are secondary endpoints

8.1.2 Investigator RECIST 1.1 Imaging Review

Radiological efficacy will be assessed by RECIST 1.1. A baseline scan should be collected during screening (Day -28 to -1) for disease staging and for use as a RECIST 1.1 baseline for the study. Computed tomography is the preferred imaging modality; however other imaging modalities (eg, bone scan, MRI scan) may be required to define progression in equivocal cases. The imaging schedule is outline in the SoA, and if an unscheduled assessment is performed and the patient has not progressed, every attempt should be made to perform the subsequent assessments according to the original imaging schedule. For patients who discontinue treatment due to toxicity or other reasons in the absence of confirmed objective progression, tumor assessments should continue according to the schedule of assessments.

8.1.3 Survival Assessments

Overall survival is defined as the time from enrollment to the date of death from any cause. Progression free survival is defined as the time from enrollment to the date of radiological confirmed progression or date of death from any cause.

Assessments for survival must be made at months 3,6, and 9 (± 1 week); month 12 (± 2 weeks); and then every 6 months thereafter (±2 weeks) following treatment discontinuation as outlined in SoA. Survival information may be obtained via telephone contact with the patient or when appropriate patient's family, or by contact with the patient's current physician. The details of first and subsequent therapies for cancer after discontinuation of treatment will be collected.

In addition, patients on treatment or in survival follow-up will be contacted following the data cutoff for the primary analysis and all subsequent survival analyses to provide complete survival data. These contacts should generally occur within 7 days of the data cutoff. If patients are confirmed to be alive or if the death date is after the data cutoff date, these patients will be censored at the date of data cutoff. Death dates may be found by checking publicly available death registries, where allowed by local regulations.

8.2 Safety assessments

Planned time points for all safety assessments are provided in the SoA.

8.2.1 Clinical safety laboratory assessments

Blood and urine samples for determination of clinical chemistry, hematology, and urinalysis will be taken at the times indicated in the assessment schedules and as clinically indicated (see the SoAs).

Clinical laboratory safety tests, including serum pregnancy tests, will be performed in a licensed clinical laboratory according to local standard procedures. Sample tubes and sample sizes may

vary depending on the laboratory method. Urine pregnancy will be performed using a licensed test (urine or serum pregnancy tests are acceptable). Abnormal clinically significant laboratory results should be repeated as soon as possible.

Additional safety samples may be collected if clinically indicated at the discretion of the investigator. The date, time of collection, and results (values, units, and reference ranges) will be recorded in the appropriate eCRF.

The laboratory variables to be measured are presented in Table 10 (clinical chemistry and hematology).

Table 10 Laboratory safety variables

**Haematology/Haemostasis (whole blood)**	**Clinical Chemistry (serum or plasma)**
B-Haemoglobin (Hb)	S/P-Creatinine
B-Leukocyte count	S/P-Bilirubin, total
B-Absolute neutrophil count	S/P-Alkaline phosphatise (ALP)
B-Absolute lymphocyte count	S/P-Aspartate transaminase (AST)
B-Platelet count	S/P-Alanine transaminase (ALT)
B-Mean cell volume (MCV)	S/P-Albumin
	S/P-Potassium
	S/P-Calcium, total
	S/P-Sodium
**Urinalysis** (macroscopic w/reflex microscopic)	S/P-Urea or Blood Urea Nitrogen (BUN)
U-Hb/Erythrocytes/Blood	S/P-Total Protein
U-Protein/Albumin	
U-Glucose	

NB. In case a patient shows an AST **or** ALT ≥3xULN together with total bilirubin ≥2xULN please refer to Appendix A. Actions required in cases of increases in liver biochemistry and evaluation of Hy's Law', for further instructions.

8.2.1.1 Coagulation

· Activated partial thromboblastin time {APTT} will be performed at screening and if clinically indicated

· International normalised ratio {INR} will be performed at screening and if clinically indicated. Patients taking warfarin may participate in this study; however, it is recommended that INR be monitored carefully at least once per week for the first month, then monthly if the INR is stable.

Each coagulation test result will be recorded in CRF.

8.2.1.2 Bone marrow or blood cytogenetic samples

Bone marrow or blood cytogenetic samples may be collected for patients with prolonged haematological toxicities.

Bone marrow analysis should include an aspirate for cellular morphology, cytogenetic analysis and flow cytometry, and a core biopsy for bone marrow cellularity. If it is not possible to conduct cytogenetic analysis or flow cytometry on the bone marrow aspirate, then attempts should be made to carry out the tests on a blood sample. Full reports must be provided by the investigator for documentation on the Patient Safety database. These data are not required to be entered into CRF.

8.2.2 Physical examinations

Physical examinations will be performed according to the assessment schedule (see the SoAs). Full physical examinations will include assessments of the head, eyes, ears, nose, and throat and the respiratory, cardiovascular, GI, urogenital, musculoskeletal, neurological, dermato;ogical, hematologic/lymphatic, and endocrine systems.

8.2.3 Vital signs

Vital signs (blood pressure [BP], pulse, temperature, and respiratory rate) will be assessed according to the assessment schedule. Body weight is also recorded at each visit when vital signs are evaluated. Height will be measured on day 1 only.

BP and pulse measurements will be assessed with a completely automated device. Manual techniques will be used only if an automated device is not available.

BP and pulse measurements should be preceded by at least 5 minutes of rest for the patient in a quiet setting without distractions (eg, television, cell phones).

Vital signs will be measured in a semi-supine position after 5 minutes of rest and will include temperature, systolic and diastolic BP, pulse, and respiratory rate. Vital signs will be recorded in the eCRF.

8.2.4 Electrocardiograms

Resting 12-lead ECGs will be recorded at screening as clinically indicated throughout the study (see the SoA). ECGs should be obtained after the patient has been in a supine position for 5 minutes and recorded while the patient remains in that position.

In case of clinically significant ECG abnormalities, including a QTcF value ≥ 470 ms, 2 additional 12-lead ECGs should be obtained over a brief period (eg, 30 minutes to confirm the finding.

Situations in which ECG results should be reported as AEs are described in section 8.3.7.

APPROVED BY THE YALE UNIVERSITY IRB 2/12/2020

APPROVED BY THE YALE UNIVERSITY IRB 2/12/2020

8.2.5 Other safety assessments

If new or worsening pulmonary symptoms (eg, dyspnea) or radiological abnormality suggestive of pneumonitis/ILD is observed, toxicity management as described in detail in the Dosing Modification and Toxicity Management Guidelines (see Section 8.4.5) will be applied. The results of the full diagnostic workup (including high-resolution computed tomography [HRCT], blood and sputum culture, haematological parameters, etc) will be captured in the eCRF. It is strongly recommended to perform a full diagnostic workup, to exclude alternative causes such as lymphangitic carcinomatosis, infection, allergy, cardiogenic edema, or pulmonary haemorrhage. In the presence of a confirmatory HRCT scans where other causes of respiratory symptoms have been excluded, a diagnosis of pneumonitis (ILD) should be considered and the Dosing Modification and Toxicity Management Guidelines should be followed.

Pneumonitis (ILD) investigation

The following assessments, and additional assessments if required, will be performed to enhance the investigation and diagnosis of potential causes of pneumonitis. The results of the assessment will be collected.

- Physical examination
- Signs and symptoms (cough, shortness of breath, and pyrexia, ETC) including auscultation for lung field will be assessed.
- Saturation of peripheral oxygen (SpO_2)
- When pneumonitis (ILD) is suspected during study treatment, the following markers should be measured where possible:
    - ILD Markers (KL-6, SP-D) and β–D-GLUCAN
    - Tumor Markers: particular tumor markers that are related to disease progression
    - Additional chemist chemistry: CRP, LDH

8.2.5.1 Serum or urine pregnancy test

Pregnancy tests on blood or urine samples will be performed for women of childbearing potential within 28 days prior to the start of study treatment, on Day 1 of the study prior to commencing treatment, on day 1 of every cycle, and at the 30 day follow up visit. Tests will be performed by the hospital's local laboratory. If results are positive the patient is ineligible/must be discontinued from study treatment immediately. Details of the pregnancy tests must be recorded in the patient's medical records.

8.3 Collection of adverse events

The Principal Investigator is responsible for ensuring that all staff involved in the study are familiar with the content of this section

AE will be reported by the patient (or, when appropriate, by a caregiver, surrogate, or the patient's legally authorized representative).

The investigator and any designees are responsible for detecting, documenting, and recording events that meet the definition of an AE or SAE. For information on how to follow/up AEs see section 8.3.3.

APPROVED BY THE YALE UNIVERSITY IRB 2/12/2020

APPROVED BY THE YALE UNIVERSITY IRB 2/12/2020

8.3.1 Method of detecting AEs and SAEs

Care will be taken not to introduce bias when detecting AEs and/or SAEs. Open-ended and non-leading verbal questioning of the patient is the preferred method to inquire about AE occurrences.

8.3.2 Time period and frequency for collecting AE and SAE information

Adverse Events will be collected from the time of signature of informed consent throughout the treatment period and until the 30-day follow-up period is completed. If an event that starts post the defined safety follow-up period noted above is considered to be due to a late onset toxicity to the study treatments then it should be reported as an AE or SAE as applicable.

All SAEs will be recorded and reported to the sponsor or designee within 24 hours, as indicated in **Error! Reference source not found.**. The investigator will submit any updated SAE data to the sponsor within 24 hours of it being available.

Investigators are not obligated to actively seek AE or SAE in former study patients. However, if the investigator learns of any SAE, including a death, at any time after a patient's last visit and he/she considers the event to be reasonably related to the Study treatment or study participation, the investigator may notify the sponsor.

The method of recording, evaluating, and assessing causality of AE and SAE and the procedures for completing and transmitting SAE reports are provided in **Error! Reference source not found.**.

8.3.2.1 Adverse events after the 30 day follow up period

For Pharmacovigilance purposes and characterisation, any SAE of MDS/AML or new primary malignancy occurring after the 30 day follow up period should be reported to AstraZeneca Patient Safety regardless of investigator's assessment of causality or knowledge of the treatment arm. Investigators will be asked during the regular follow up for overall survival if the patient has developed MDS/AML or a new primary malignancy and prompted to report any such cases.

At any time after a patient has completed the study, if an Investigator learns of any SAE including sudden death of unknown cause, and he/she considers there is a reasonable possibility that the event is causally related to the investigational product, the investigator should notify AstraZeneca, Patient Safety.

If patients who are gaining clinical benefit are allowed to continue study treatment post study completion then all SAEs must continue to be collected and reported to Patient Safety in the usual timeframe.

Otherwise, after study treatment completion (i.e. after any scheduled post treatment follow-up period has ended) there is no obligation to actively report information on new AEs or SAEs occurring in former study patients. This includes new AEs/SAEs in patients still being followed up for survival but who have completed the post treatment follow up period (30 days).

APPROVED BY THE YALE UNIVERSITY IRB 2/12/2020

APPROVED BY THE YALE UNIVERSITY IRB 2/12/2020

8.3.3 Follow-up of AEs and SAEs

After the initial AE/SAE report, the investigator is required to proactively follow each patient at subsequent visits/contacts. All AEs/SAEs, will be followed until resolution, stabilization, the event is otherwise explained, or the patient is lost to follow-up

Any AEs that are unresolved at the patient's last visit in the study are followed up by the investigator for as long as medically indicated (this may be beyond the 30 days after the last dose of study treatment), but without further recording in the CRF. AstraZeneca retains the right to request additional information for any patient with ongoing AE(s)/SAE(s) at the end of the study, if judged necessary.

Any SAE or non-serious adverse event that is ongoing at the time of the 30-day follow up, must be followed up to resolution unless the event is considered by the investigator to be unlikely to resolve, or the patient is lost to follow up. AstraZeneca retains the right to request additional information for any patient with ongoing AE(s)/SAE(s) at the end of the study, if judged necessary

8.3.4 Adverse event data collection

The following variables will be collect for each AE;

- AE (verbatim)
- The date when the AE started and stopped
- CTCAE v.5.0 grade and changes in CTCAE v.5.0grade
- Whether the AE is serious or not
- Investigator causality rating against the Investigational Product(s) (yes or no), comparator/combination drug (yes/no)
- Action taken with regard to Investigational Product(s) / comparator/combination drug
- AE caused subject's withdrawal from study (yes or no)
- Outcome.

In addition, the following variables will be collected for SAEs:

- Date AE met criteria for serious AE
- Date Investigator became aware of serious AE
- AE is serious due to
- Date of hospitalisation

APPROVED BY THE YALE UNIVERSITY IRB 2/12/2020

APPROVED BY THE YALE UNIVERSITY IRB 2/12/2020

- Date of discharge
- Probable cause of death
- Date of death
- Autopsy performed
- Causality assessment in relation to Study procedure(s)
- Causality assessment to other medication'
- Description of AE

The grading scales found in the revised NCI CTCAE version 5.0 will be utilized for all events with an assigned CTCAE grading. For those events without assigned CTCAE grades, the recommendation in the CTCAE criteria that converts mild, moderate, and severe events into CTCAE grades should be used. A copy of the CTCAE version 5.0 can be downloaded from the Cancer Therapy Evaluation Program website (http://ctep.cancer.gov).

8.3.5 Causality collection

The Investigator will assess causal relationship between Investigational Product and each Adverse Event, and answer 'yes' or 'no' to the question 'Do you consider that there is a reasonable possibility that the event may have been caused by the investigational product or combination drug?'

For SAEs, causal relationship will also be assessed for other medication and study procedures. Note that for SAEs that could be associated with any study procedure the causal relationship is implied as 'yes'.

A guide to the interpretation of the causality question is found in **Error! Reference source not found.**.

8.3.6 Adverse events based on signs and symptoms

All AEs spontaneously reported by the patient or care provider, reported in response to the open question from the study site staff: "Have you had any health problems since the previous visit/you were last asked?," or revealed by observation will be collected and recorded in the CRF. When collecting AEs, the recording of diagnoses is preferred (when possible) to recording a list of signs and symptoms. However, if a diagnosis is known and there are other signs or symptoms that are not generally part of the diagnosis, the diagnosis and each sign or symptom will be recorded separately.

8.3.7 Adverse events based on examinations and tests

The results from the protocol mandated laboratory tests and vital signs will be summarised in the clinical study report (CSR). Deterioration as compared to baseline in protocol-mandated

APPROVED BY THE YALE UNIVERSITY IRB 2/12/2020

laboratory values and vital signs should therefore only be reported as AEs if they fulfil any of the SAE criteria or are the reason for discontinuation of treatment with the investigational products.

If deterioration in a laboratory value/vital sign is associated with clinical signs and symptoms, the sign or symptom will be reported as an AE and the associated laboratory result/vital sign will be considered as additional information. Wherever possible the reporting Investigator uses the clinical, rather than the laboratory term (e.g., anaemia versus low haemoglobin value). In the absence of clinical signs or symptoms, clinically relevant deteriorations in non-mandated parameters should be reported as AE(s).

Deterioration of a laboratory value, which is unequivocally due to disease progression, should not be reported as an AE/SAE.

Any new or aggravated clinically relevant abnormal medical finding at a physical examination as compared with the baseline assessment will be reported as an AE unless unequivocally related to the disease under study, see sections 8.3.9 and 8.310.

8.3.8 Hy's law

Cases where a patient shows elevations in liver biochemistry may require further evaluation and occurrences of AST or ALT ≥ 3xULN together with total bilirubin ≥ 2xULN may need to be reported as SAEs. Please refer to Appendix A for further instruction on cases of increases in liver biochemistry and evaluation of Hy's Law

8.3.9 Disease progression

Disease progression can be considered as a worsening of a patient's condition attributable to the disease for which the investigational product is being studied. It may be an increase in the severity of the disease under study and/or increases in the symptoms of the disease. The development of new, or progression of existing metastasis to the primary cancer under study should be considered as disease progression and not an AE. Events, which are unequivocally due to disease progression, should not be reported as an AE during the study.

8.3.10 New Cancers

The development of a new primary cancer should be reported as an AE (see Section 8.3.13 Olaparib Adverse Events of Special Interest) and would in most cases meet seriousness criteria (with the exception of some non-melanoma skin cancers). New primary malignancies are those that are not the primary reason for the administration of the study treatment and have developed after the inclusion of the patient into the study. They do not include metastases of the original cancer. Symptoms of metastasis or the metastasis itself should not be reported as an AE/SAE, as they are considered to be disease progression.

8.3.11 Lack of efficacy

When there is deterioration in the cancer, for which the study treatment(s) is being used, may be uncertainty as to whether this is lack of efficacy or an AE. In such cases, unless the the reporting physician considers that the study treatment contributed to the deterioration of the condition, or

local regulations state to the contrary, the deterioration should be considered to be a lack of efficacy and not an AE.

8.3.12 Deaths

All deaths that occur during the study, or within the protocol-defined 30-day post-study follow-up period after the administration of the last dose of study treatment, must be reported as follows:

- Death clearly the result of disease progression should be reported to principal investigator and should be documented in the eCRF but should not be reported as an SAE.
- Where death is not due (or not clearly due) to progression of the disease under study, the AE causing the death must be reported to the principal investigator and Astra Zeneca as a SAE within **24 hours** (see Section 8.4.1 for further details). The report should contain a comment regarding the co-involvement of progression of disease, if appropriate, and should assign main and contributory causes of death. This information can be captured in the eCRF.

Deaths with an unknown cause should always be reported as a SAE. A post mortem maybe helpful in the assessment of the cause of death, and if performed a copy of the post-mortem results should be forwarded to AstraZeneca within the usual timeframes.

8.3.13 Olaparib adverse events of special interest

Adverse events of special interest [AESI] are events of scientific and medical interest specific to the further understanding of olaparib's safety profile and require close monitoring and rapid communication by the investigators to AstraZeneca. Adverse Events of Special Interest for olaparib are the Important Potential Risks of MDS/AML, new primary malignancy (other than MDS/AML) and pneumonitis.

A questionnaire will be sent to the principal investigator if an AESI is reported, as an aid to provide further detailed information on the event. During the study there may be other events identified as AESIs that require the use of a questionnaire to help characterise the event and gain a better understanding regarding the relationship between the event and study treatment.

8.4 Safety reporting and medical management

8.4.1 Reporting of serious adverse events

All SAEs have to be reported, whether or not considered causally related to the investigational product, or to the study procedure(s). All SAEs will be recorded in the CRF.

If any SAE occurs in the course of the study, then investigators or other site personnel inform the appropriate AstraZeneca representatives within one day i.e., immediately but **no later than 24 hours** of when he or she becomes aware of it.

APPROVED BY THE YALE UNIVERSITY IRB 2/12/2020

APPROVED BY THE YALE UNIVERSITY IRB 2/12/2020

The designated AstraZeneca representative works with the investigator to ensure that all the necessary information is provided to the AstraZeneca Patient Safety data entry site **within 1 calendar day** of initial receipt for fatal and life threatening events **and within 5 calendar days** of initial receipt for all other SAEs.

For fatal or life-threatening adverse events where important or relevant information is missing, active follow-up is undertaken immediately. Investigators or other site personnel inform AstraZeneca representatives of any follow-up information on a previously reported SAE within one calendar day i.e., immediately but **no later than 24 hours** of when he or she becomes aware of it.

For further guidance on the definition of a SAE, see **Error! Reference source not found.**.

8.4.2 Pregnancy

All pregnancies and outcomes of pregnancy should be reported to AstraZeneca except for:

- If the pregnancy is discovered before the study patient has received any study drug Pregnancies in the partner of male patients (see Section 5.3 for lifestyle restrictions for male patients with a female partner of childbearing potential and Section 8.4.2.2. for restrictions on male patients fathering a child)

If a pregnancy is reported, the investigator should inform Astra Zeneca within 24 hours of learning of the pregnancy.

Abnormal pregnancy outcomes (eg, spontaneous abortion, foetal death, stillbirth, congenital anomalies, ectopic pregnancy) are considered SAEs.

8.4.2.1 Maternal exposure

If a patient becomes pregnant during the course of the study, the investigational products should be discontinued immediately.

Pregnancy itself is not regarded as an adverse event unless there is a suspicion that the investigational product under study may have interfered with the effectiveness of a contraceptive medication. Congenital abnormalities/birth defects and spontaneous miscarriages should be reported and handled as SAEs. Elective abortions without complications should not be handled as AEs. The outcome of all pregnancies (spontaneous miscarriage, elective termination, ectopic pregnancy, normal birth or congenital abnormality) occurring from the date of the first dose of study medication until 6 months after the last dose of study medication should be followed up and documented even if the patient was discontinued from the study.

If any pregnancy occurs in the course of the study, then the Investigator or other site personnel informs overall principal investigator who will discuss with the designated AstraZeneca representative within 1 day i.e., immediately but **no later than 24 hours** of when he or she becomes aware of it.

APPROVED BY THE YALE UNIVERSITY IRB 2/12/2020

APPROVED BY THE YALE UNIVERSITY IRB 2/12/2020

The designated AstraZeneca representative works with the Investigator to ensure that all relevant information is provided to the AstraZeneca Patient Safety within 1 or 5 calendar days for SAEs (see Section 9.2.5) and within 30 days for all other pregnancies.

The same timelines apply when outcome information is available.

8.4.2.2 Paternal exposure

Male patients should refrain from fathering a child or donating sperm during the study and for 3 months following the last dose of the investigational products.

Pregnancy of the patient's partners is not considered to be an adverse event. However, the outcome of all pregnancies (spontaneous miscarriage, elective termination, ectopic pregnancy, normal birth or congenital abnormality) occurring from the date of the first dose until 3 months after the last dose should, if possible, be followed up and documented.

8.4.3 Overdose

There is currently no specific treatment in the event of overdose with olaparib or TMZ and possible symptoms of overdose are not established.

Olaparib and TMZ must only be used in accordance with the dosing recommendations in this protocol. Any dose or frequency of dosing that exceeds the dosing regimen specified in this protocol should be reported as an overdose. The Maximum Tolerated Dose is Olaparib 150 mg mg twice daily (tablet) (Days 1-21) with TMZ 75 mg/m^2 (Days 1-7).

Adverse reactions associated with overdose should be treated symptomatically and should be managed appropriately.

- An overdose with associated AEs is recorded as the AE diagnosis/symptoms on the relevant AE modules in the CRF.

If an overdose on an AstraZeneca study drug occurs in the course of the study, then the Investigator or other site personnel inform appropriate AstraZeneca representatives immediately, or **no later than 24 hours** of when he or she becomes aware of it.

The designated AstraZeneca representative works with the Investigator to ensure that all relevant information is provided to AstraZeneca Patient Safety.

For overdoses associated with a SAE, the standard reporting timelines apply, see Section 8.3.2. For other overdoses, reporting must occur within 30 days.

8.4.4 Medication error

If a medication error occurs in the course of the study, then the Investigator or other site personnel informs the appropriate AstraZeneca representatives within 1 day i.e., immediately but no later than 24 hours of when he or she becomes aware of it.

APPROVED BY THE YALE UNIVERSITY IRB 2/12/2020

APPROVED BY THE YALE UNIVERSITY IRB 2/12/2020

The designated AstraZeneca representative works with the Investigator to ensure that all relevant information is completed within 1 (Initial Fatal/Life-Threatening or follow up Fatal/Life-Threatening) or 5 (other serious initial and follow up) calendar days if there is an SAE associated with the medication error (see Section 8.3.2) and within 30 days for all other medication errors.

The definition of a Medication Error can be found in **Error! Reference source not found.**.

8.4.5 Management of adverse events related to Olaparib and TMZ

Any toxicity observed during the course of the study could be managed by interruption of the dose of the attributed study treatment or dose reductions. Repeat dose interruptions are allowed as required, for a maximum of 4 weeks on each occasion. If the interruption is any longer, the study team must be informed. Olaparib treatment can be dose reduced to 100 mg twice daily. If the reduced dose of 100 mg twice daily is not tolerable, olaparib can be further dose reduced to 100 mg daily, and if this is not tolerable no further dose reduction is allowed and study treatment should be discontinued. Temozolomide can be dose reduced to 60 mg/m^2 (Days 1-7), and if this is not tolerable TMZ can be further dose reduced to 50 mg/m^2 (Days 1-7). The following tables should be applied if an AE is deemed to be at least "possibly related" to olaparib, TMZ, or both. If an AE is only attributed to one of the agents the other agent may be continue at an unadjusted dose.

Once dose is reduced, escalation is not permitted (except following concomitant treatment with CYP3A4 inhibitors – see Section 6.5

8.4.5.1 Management of haematological toxicity

Management of anaemia

Table 11 Management of anaemia

Haemoglobin	Action to be taken
**Hb < 10** *but* **≥ 8 g/dl (CTCAE Grade 2)***	**First occurrence:**<br>Give appropriate supportive treatment and investigate causality.<br>Investigator judgement to continue olaparib and TMZ with supportive treatment (eg transfusion) *or* interrupt dose for a maximum of 4 weeks. TMZ and olaparib can be restarted if Hb has recovered to > 9g/dl.<br>**Subsequent occurrences:**<br>If Hb< **10** *but* **≥ 9 g/dl** investigator judgement to continue olaparib and TMZ with supportive treatment (eg transfusion) *or* dose interrupt (for max of 4 weeks) and upon recovery dose reduction may be considered for olaparib (to **100 mg twice daily** as a first step and to **100 mg once daily**) and for TMZ (**60 mg/m^2** as a first step and to **50 mg/m^2** as a second step).<br>If Hb< **9** *but* **≥ 8 g/dl**, dose interrupt (for max of 4 weeks) until Hb ≥ 9 g/dl and upon recovery dose reduction for olaparib may be considered (to **100 mg twice daily** as a first step and to **100 mg once daily** as a second step). For TMZ a dose reduction may be considered (to **TMZ 60 mg/** m^2 as a first step and to **50 mg/ m^2** as a second step).

APPROVED BY THE YALE UNIVERSITY IRB 2/12/2020

Haemoglobin	Action to be taken
**Hb < 8 g/dl (CTCAE Grade 3)***	Give appropriate supportive treatment (e.g. transfusion) and investigate causality.<br>Interrupt olaparib and TMZ for a maximum of 4 weeks until improved to Hb ≥ 9 g/dl.<br>Upon recovery, for olaparib dose reduce to **100 mg twice daily** as a first step and to **100 mg once daily** as a second step in the case of repeat Hb decrease. For TMZ dose reduce to **TMZ 60 mg/m^2** as a first step and to **50 mg/m^2** as a second step.

* If the starting dose of TMZ is lowered as described in section 4.2 the TMZ doses should be adjusted per table 7 based on the starting dose.

Common treatable causes of anaemia (e.g., iron, vitamin B12 or folate deficiencies and hypothyroidism) should be investigated and appropriately managed. In some cases management of anaemia may require blood transfusions. For cases where patients develop prolonged haematological toxicity (≥2 week interruption/delay in study treatment due to CTC grade 3 or worse anaemia and/or development of blood transfusion dependence), refer to guidance later in this section for further management.

Management of neutropenia, leukopenia and thrombocytopenia

Table 12 Management of neutropenia, leukopenia and thrombocytopenia

Toxicity	Study treatment dose adjustment
CTCAE Grade 1-2	Investigator judgement to continue treatment or if dose interruption, this should be for a maximum of 4 weeks; appropriate supportive treatment and causality investigation
CTCAE Grade 3-4*	Dose interruption until recovered to CTCAE gr 1 or better for a maximum of 4 weeks. If repeat CTCAE grade 3-4 occurrence, dose reduce olaparib to **100 mg twice daily** as a first step and **100 mg once daily** as a second step and dose reduce **TMZ to 60 mg/m^2** as a first step and **50 mg/m^2** as a second step.

* If the starting dose of TMZ is lowered as described in section 4.2 the TMZ dose should be lowered per table 7 based on the starting dose.

Adverse event of neutropenia and leukopenia should be managed as deemed appropriate by the investigator with close follow up and interruption of study drug if CTCAE grade 3 or worse neutropenia occurs.

Primary prophylaxis with Granulocyte colony-stimulating factor (G-CSF) is not recommended, however, if a patient develops febrile neutropenia, study treatment should be stopped and appropriate management including G-CSF should be given according to local hospital guidelines. Please note that G-CSF should not be used within at least 24 h (7 days for pegylated G-CSF) of the last dose of TMZ or olaparib unless absolutely necessary.

Platelet transfusions, if indicated, should be done according to local hospital guidelines.

For cases where patients develop prolonged haematological toxicity (≥2 week interruption/delay in study treatment due to CTCAE grade 3 or worse), refer to guidance later in this section for the management of this.

Management of prolonged haematological toxicities while on study treatment

If a patient develops prolonged haematological toxicity such as:

- ≥2 week interruption/delay in study treatment due to CTCAE grade 3 or worse anaemia and/or development of blood transfusion dependence
- ≥2 week interruption/delay in study treatment due to CTCAE grade 3 or worse neutropenia (ANC < 1 x 10^9/L)
- ≥2 week interruption/delay in study treatment due to CTCAE grade 3 or worse thrombocytopenia and/or development of platelet transfusion dependence (Platelets < 50 x 10^9/L)

Check weekly differential blood counts including reticulocytes and peripheral blood smear. If any blood parameters remain clinically abnormal after 4 weeks of dose interruption, the patient should be referred to haematologist for further investigations. Bone marrow analysis and/or blood cytogenetic analysis should be considered at this stage according to standard haematological practice. Study treatment should be discontinued if blood counts do not recover to CTCAE gr 1 or better within 4 weeks of dose interruption.

Development of a confirmed myelodysplastic syndrome or other clonal blood disorder should be reported as an SAE and full reports must be provided by the investigator to AstraZeneca Patient Safety. Study treatment should be discontinued if patient's diagnosis of MDS and/or AML is confirmed.

8.4.5.2 Management of non-haematological toxicity

Repeat dose interruptions are allowed as required, for a maximum of 4 weeks on each occasion. If the interruption is any longer than this the overall principal investigator must be informed and confirm with the treating physician whether study treatments should continue. Where toxicity reoccurs following re-challenge with study treatment, and where further dose interruptions are considered inadequate for management of toxicity, then the patient should be considered for dose reduction or must permanently discontinue study treatment.

Olaparib can be dose reduced to 100 mg twice daily as a first step and to 100 mg twice daily as a second step, while TMZ can be dose reduced to 60 mg/m2 as a first step and 50 mg/m2 as a second step. Treatment must be interrupted if any NCI-CTCAE grade 3 or 4 adverse event occurs which the investigator considers to be related to administration of study treatment. If the investigator considers the adverse event to be related to only one of the study medications the

APPROVED BY THE YALE UNIVERSITY IRB 2/12/2020

APPROVED BY THE YALE UNIVERSITY IRB 2/12/2020

other medication can continue uninterrupted after discussion with the overall principal investigator if felt to be safe in the opinion of the treating investigator.

Management of new or worsening pulmonary symptom

If new or worsening pulmonary symptoms (e.g., dyspnoea) or radiological abnormalities occur in the absence of a clear diagnosis, an interruption in study treatment dosing is recommended and further diagnostic workup (including a high resolution CT scan) should be performed to exclude pneumonitis.

Following investigation, if no evidence of abnormality is observed on CT imaging and symptoms resolve, then study treatment can be restarted, if deemed appropriate by the investigator. If significant pulmonary abnormalities are identified, these need to be discussed with the principal investigator.

Management of nausea and vomiting

Events of nausea and vomiting are known to be associated with olaparib treatment. These events are generally mild to moderate (CTCAE grade 1 or 2) severity, intermittent and manageable on continued treatment. The first onset generally occurs in the first month of treatment for nausea and within the first 6 months of treatment for vomiting. For nausea, the incidence generally plateaus at around 9 months, and for vomiting at around 6 to 7 months.

No routine prophylactic anti-emetic treatment is required at the start of study treatment, however, patients should receive appropriate anti-emetic treatment at the first onset of nausea or vomiting and as required thereafter, in accordance with local treatment practice guidelines. Alternatively, olaparib and TMZ tablets can be taken with a light meal/snack (ie 2 pieces of toast or a couple of biscuits).

As per international guidance on anti-emetic use in cancer patients (ESMO, NCCN), generally a single agent antiemetic should be considered eg dopamine receptor antagonist, antihistamines or dexamethasone.

Interruptions for intercurrent non-toxicity related events

Study treatment dose interruption for conditions other than toxicity resolution should be kept as short as possible. If a patient cannot restart study treatment within 4 weeks for resolution of intercurrent conditions not related to disease progression or toxicity, the case should be discussed with overall principal investigator.

All dose reductions and interruptions (including any missed doses), and the reasons for the reductions/interruptions are to be recorded in the eCRF.

Study treatment should be stopped at least 3 days prior to planned surgery. After surgery study treatment can be restarted when the wound has healed. No stoppage of study treatment is required for any needle biopsy procedure.

APPROVED BY THE YALE UNIVERSITY IRB 2/12/2020

Study treatment should be discontinued for a minimum of 3 days before a patient undergoes radiation treatment. Study treatment should be restarted within 4 weeks as long as any bone marrow toxicity has recovered.

Because the AEs related to olaparib may include asthenia, fatigue and dizziness, patients should be advised to use caution while driving or using machinery if these symptoms occur.

Table 13 Dose reductions for olaparib

Initial Dose	Following re-challenge post interruption: Dose reduction 1	Dose reduction 2
150 mg twice daily	100 mg twice daily	100 mg once daily

Table 14 Dose reductions for TMZ

Initial Dose	Following re-challenge post interruption: Dose reduction 1	Dose reduction 2
75 mg/m^2 (days 1-7)	60 mg/m^2 (days 1-7)	50 mg/m^2 (days 1-7)
*60 mg/m^2 (days 1-7)	50 mg/m^2 (days 1-7)	40 mg/m^2 (days 1-7)
*50 mg/m^2 (days 1-7)	40 mg/m^2 (days 1-7)	30 mg/m^2 (days 1-7)

* If the starting dose of TMZ is lowered as described in section 4.2.

8.4.5.3 Renal impairment

If subsequent to study entry and while still on study therapy, a patient’s estimated CrCl falls below the threshold for study inclusion (≥51 ml/min), retesting should be performed promptly.

A dose reduction is recommended for patients who develop moderate renal impairment (calculated creatinine clearance by Cockcroft-Gault equation or based on a 24 hour urine test of between 31 and 50 ml/min) for any reason during the course of the study: the dose of olaparib should be reduced to 100 mg twice daily.

No dose reductions for TMZ are required for moderate to severe renal impairment, although caution is advised in cases of CrCl < 36 ml/min. Because the CrCl determination is only an estimate of renal function, in instances where the CrCl falls to between 31 and 50 mL/min, the investigator should use his or her discretion in determining whether a dose change or discontinuation of therapy is warranted.

Olaparib has not been studied in patients with severe renal impairment (creatinine clearance ≤ 30 ml/min) or end-stage renal disease; if patients develop severe impairment or end stage disease it is recommended that study treatment be discontinued.

APPROVED BY THE YALE UNIVERSITY IRB 2/12/2020

8.5 Pharmacokinetics

Pharmacokinetic parameters are not evaluated in this study.

8.6 Pharmacodynamics

Pharmacodynamic parameters are not evaluated in this study.

8.7 Genetics

8.7.1 Collection of optional genetic samples

If the patient agrees to participate in the optional genetic research study, a blood sample will be collected. Participation is optional. Patients who do not wish to participate in the genetic research may still participate in the study. In the event of DNA extraction failure, a replacement genetic sample may be requested from the patient. Signed informed consent will be required to obtain a replacement sample unless it was included in the original consent.

The blood and/or buccal swab sample for genetic research will be obtained from the patients at the screening visit. Although DNA variants are stable parameter, early sample collection is preferred to avoid introducing bias through excluding patients who may withdraw due to an adverse event (AE), such patients would be important to include in any genetic analysis. If for any reason the sample is not drawn at the screening visit, it may be taken at any visit until the last study visit.

Samples will be collected, labelled, stored and shipped as detailed in the Laboratory Manual.

8.7.2 Storage and destruction of genetic samples

The processes adopted for the coding and storage of samples for genetic analysis are important to maintain patient confidentiality. Samples may be stored for a maximum of 15 years or as per local regulations from the date of the patient's last visit, after which they will be destroyed. DNA is a finite resource that may be used up during analyses. The results of any further analyses will be reported either in the CSR itself or as an addendum or separately in a scientific report or publication.

No personal details identifying individual will be available to AstraZeneca or designated organizations working with the DNA.

8.8 Biomarkers

<u>MGMT Promoter Hypermethylation Testing (integral biomarker):</u>

Existing archival tumor biopsy samples will be used for pre-screening, and when slides are provided at least 10 unstained slides must be submitted. Methylation status of the human MGMT promoter on chromostome 10 will be performed in formal fixed paraffin embedded tissue, by sodium bisulfite conversion followed by multiplex PCR. The analysis will be performed in the CLIA certified pathology laboratory of Dr. Pei Hui at Yale University School of Medicine. Please refer to the Laboratory Services Manual for additional details regarding testing for MGMT Promoter Hypermethylation.

APPROVED BY THE YALE UNIVERSITY IRB 2/12/2020

Exploratory biomarkers:

By participating in this study, the patient consents to the mandatory collection of tumor tissue (pre-treatment biopsy) and use of donated biological samples as described here. Tissue samples will be obtained from all screened patients.

Mandatory tumor and blood biomarkers to be evaluated to support the exploratory objectives of the study are described below. Alternative biomarkers may be evaluated as determined by additional data associated with disease progression/recurrence or response to the investigational treatment.

Biomarker assessments that may have the potential to identify patients likely to respond to treatment with olaparib and TMZ (determined from other studies with olaparib +/- TMZ) will be investigated to determine a patient's biomarker status and for possible correlation with efficacy endpoints.

- Tumour biopsies will be taken at baseline and at progression for investigation of next generation sequencing profiling, patterns of DNA methylation and gene expression, γH2AX, changes in the immune composition of the tumor microenvironment, resistance mechanisms, and other exploratory endpoints to evaluate their association with the observed clinical responses to olaparib and TMZ.

- Please refer to the Central Laboratory Services Manual for further details regarding sample collection, shipping and storage.

Biomarkers will be tested in blood to evaluate their association with the observed clinical responses to olaparib and TMZ.

In addition, blood will be collected and an analysis may be performed on biomarker variants thought to play a role in DNA repair including, but not limited to, candidate genes/genome-wide analysis for RNA, serum analytes, or tissue biomarkers to evaluate their association with observed clinical responses to olaparib and TMZ.

Other samples may be used for research to develop methods, assays, prognostics and/or companion diagnostics related to MGMT promoter hypermethylation / silencing and DNA repair.

Any sample material remaining after completion of analyses to fulfil the study objectives may be used for optional exploratory research to develop methods, assays, prognostics, and/or companion diagnostics related to colorectal cancer, MGMT promoter hypermethylation / silencing, PARP, and DNA repair.

The biomarker data will have unknown clinical significance and the results will not be provided to patients, their family members, any insurance company or employer.

The exploratory biomarker plan is described by sample type below.

APPROVED BY THE YALE UNIVERSITY IRB 2/12/2020

APPROVED BY THE YALE UNIVERSITY IRB 2/12/2020

Fresh Tumor Tissue

A sample of fresh tumor tissue will be used to create colorectal carcinoma organoids.

Whole blood gene expression

Whole blood samples will be obtained from all patients at the time points described in the SoA. The focus is likely to be given to the expression of DNA repair genes including but not limited to MGMT and immunomodulatory markers. Correlation with outcome data will be completed on select candidates, and predictive markers, with the aim of identifying useful expression thresholds for identifying patients likely to receive benefit.

Serum-based markers

Serum will be obtained to explore the expression of cytokines and chemokine.

Plasma-based markers (ctDNA, circulating soluble factors)

Plasma will be obtained from all patients as described in the SoAs. The concentration of a panel of relevant cytokines, chemokines, and other DNA damage and immune-related markers may be assessed. Plasma may also be used to evaluate mutant circulating tumor DNA. Overall mutational burden and/or somatic mutations/genomic alterations in plasma may be assessed using state-of-the-art methodologies. Such measurements may be correlated with response.

8.8.1 Storage, re-use and destruction of biomarker samples

Samples will be stored for a maximum of 15 years from the date of the last patient's last visit, after which they will be destroyed. The results of this biomarker research will be reported either in the CSR itself or as an addendum, or separately in a scientific report or publication. The results of this biomarker research may be pooled with biomarker data from other studies with the study drug to generate hypotheses to be tested in future research.

9. STATISTICAL CONSIDERATIONS

9.1 Statistical hypotheses

The primary objective of this study is to assess the efficacy of temozolomide in combination with olaparib in subjects with MGMT promoter hypermethylated advanced colorectal cancer who have progressed on at least one line of systemic chemotherapy by overall response rate as assessed per RECIST v.1.1. We hypothesize that the underlying response rate to the combination therapy is 25% or greater (H_1) compared to a null hypothesis of 5% (H_0) based on historical controls.

9.2 Sample size determination

The sample size estimation for the single-arm of this phase II study is based on the primary endpoint of objective response rate (ORR). The protocol plans to enroll a total of 30 evaluable patients at the recommended dose level in a single-arm open label phase 2 study Simon's Two-

APPROVED BY THE YALE UNIVERSITY IRB 2/12/2020

APPROVED BY THE YALE UNIVERSITY IRB 2/12/2020

Stage optimal design. There will be screening for eligibility and we will examine several secondary endpoints.

Screen: To be eligible for this trial, patients will first need to be screened for the MGMT promoter hypermethylation and/or MGMT lack of expression. We anticipate at least 20% of those screened will exhibit an appropriate biomarker as well as meet eligibility criteria. To obtain 30 eligible patients, the mean number of patients to be screened is 150. Using the negative binomial distribution as reference, there is a 6% probability more than 190 subjects will need to be screened to attain the 30 eligible patients.

The **primary endpoint** is the objective response rate to the combined therapy and will be assessed in a Simon's two-stage optimal design. Published, historical records anticipate a response rate of 5% or lower and we will assume a null hypothesis of 5%. We will reject this null hypothesis in favor of a higher rate if we observe 4 or more responses in the evaluable patient sample of 30. The trial will terminate early if we do not observe 1 or more responses in the first 9 evaluable patients. The probability of 4 or more responses in a sample of 30 has probability .049, which is the significance level. If the true, underlying response rate is 25% or greater, then this trial has power 90%. The expected sample size is 17 under the null hypothesis. There is a 63% chance of stopping early under the null hypothesis.

9.3 Populations for analyses

Table 15.

Outcome variable	Populations
Efficacy Data	
ORR	Efficacy analysis set (ITT population)
PFS	Efficacy analysis set (ITT population)
OS	Efficacy analysis set (ITT population)
Safety Data	
AEs	Safety analysis set
ECOG performance status	Safety analysis set

AE Adverse events; ORR Overall response rate; PFS Progression free survival; OS Overall survival; ECOG Eastern Cooperative Oncology Group; ITT Intention to treat

All Subject Analysis Set

APPROVED BY THE YALE UNIVERSITY IRB 2/12/2020

The All Subject Analysis Set will include information from all screened subjects, including those who did not meet the study entry criteria or did not receive a study treatment. This data set will only be used for disposition in which all screened subjects are accounted for.

Efficacy Analysis Set (ITT population)

The Efficacy Analysis Set will include all subjects who receive any amount of study treatment for efficacy analyses. Efficacy analyses will be based on the actual treatment received. The ORR analysis will be based on subjects who are in the Efficacy Analysis Set and who had disease assessment at baseline and at least 1 follow up disease assessment or subjects who died or stopped treatment before the first scheduled disease assessment due to clinical progression or toxicity.

Safety Analysis Set

The Safety Analysis Set will include data from all subjects who receive any amount of study drug. Safety analysis will be based on the actual treatment received. All data will be included, and no subjects excluded because of protocol violations.

Schedule of Analysis

This is an open-label study. Ongoing analyses to help manage the study and support the DSMC reviews for safety and antitumor activity will be undertaken. A final analysis will be conducted after all subjects have completed the study.

Disposition

The number and percentage (n, %) of subjects enrolled, treated, lost to follow-up, and withdrawn from treatment or discontinued from the study (with reason) will be summarized. The sample sizes for the Efficacy Analysis Set, Safety Analysis Set will be clearly identified. All subjects in the All Subject Analysis Set will be included in the disposition analysis.

9.4 Statistical analyses

We will use intention to treat (ITT) analysis for the primary and secondary endpoints.

9.4.1 Efficacy analyses

Primary Endpoint: Overall response rate (ORR) by RECIST v1.1 criteria for combined therapy of olaparib and temozolomide.

Analytic Plan for Primary Endpoint:

The objective response is defined as a complete or partial response, as determined by investigator assessment using RECIST v 1.1 and confirmed by repeated assessment > 4 weeks after initial documentation. Patients with missing or no response assessments are classified as non-responders. The trial will terminate early if we do not observe 1 or more responses in the first 9 evaluable patients. The ORR will be estimated using the 95% confidence interval (CI) based on Wilson's method. A 5% 2-sided alpha will be used.

Secondary Endpoints: PFS, OS, Safety

Analysis Plans for Secondary Endpoints:

Secondary endpoints include overall survival, progression-free survival, and toxicity. Survival endpoints will be plotted using the Kaplan-Meier, product-limit method and compared across clinical measurements using Cox proportional hazards regression. Time to progression begins at the time of initial treatment and ends at the earliest of withdrawal, diagnosed progression, or death. For PFS subjects who are alive and have not progressed will be censored at the date of their last RECIST tumor assessment. For OS subjects who are alive will be censored at the date of their last survival assessment. Toxicity rates will be compared to established rates for these treatments and summarized using means and 95% confidence intervals.

9.4.2 Safety analyses

Adverse events (AEs) will be listed and tabulated for patients on treatment. AEs will be classified based on the likelihood that they are treatment-related. NCI toxicity Grade 3 and Grade 4 laboratory abnormalities will also be listed.

9.4.3 Other analyses

Exploratory endpoints:

Genetic markers will be plotted using heat-maps and statistically compared across responders and non-responders using the Benjamini-Hochberg correction for multiplicity of p-values.

9.4.4 Methods for multiplicity control

This is a single arm study with an integral biomarker therefore no adjustments for multiplicity are planned other than that mentioned in section 9.4.3 for the exploratory endpoints.

9.5 Interim analyses

An interim efficacy analysis will be performed after the first 9 patients are evaluated to determine whether or not to terminate the trial for futility, as noted in section 9.4.

9.5.1 Data monitoring committee (DMC)

The study principal investigator and YCCI are responsible for monitoring the performance of all of the participating investigators. This will be performed by conducting a study site initiation visit as well as regularly scheduled monitoring visits and/or remote monitoring throughout the life of the protocol. At the end of the trial, the monitor will then perform a study site close-out visit.

YCCI will utilize their institution's initiation, monitoring and close-out visit reports. Following each site visit, a visit report will be generated containing information on site activities, and a summary of pertinent points and action items together with a copy of the follow-up letter will be sent to each investigative site.

During these monitoring visits, some of the items that will be reviewed are the following:

APPROVED BY THE YALE UNIVERSITY IRB 2/12/2020

• Training of the sites

• Site personnel qualifications to participate in the trial

• That study related documents are current

• That regulatory compliance is accomplished

• That each subject has signed the informed consent

• That the current and approved protocol is complied with (including reporting and logging of all protocol deviations)

• That all SAEs and AEs have been reported to the local regulatory and Ethics/IRB Committees, YCCI and Astra Zeneca, as appropriate

• That source documentation matches CRFs

• That required procedures for study drug accountability, distribution, and storage are followed.

YCCI will document the required study monitoring activities in a Study Monitoring Plan.

9.5.2 Safety Monitoring

The Yale Cancer Center Data and Safety Monitoring Committee (DSMC) will provide the primary oversight of data and safety monitoring. The Yale DSMC will review and monitor compliance, toxicity and deviations from this study. The committee is composed of clinical specialists with experience in oncology and who have no direct relationship with the study. Information that raises any questions about participant safety will be addressed with the Principal Investigator.

The DSMC will review this protocol at a minimum of once every six months. Information to be provided to the committee includes: a study narrative by the PI, a summary DSMC report produced by OnCore (which includes participant accrual, response, trial status history, SAEs, Adverse Events, Deviations and survival); audit results, and monitoring reports as applicable. Other information (e.g. scans, laboratory values) will be provided upon request.

The Yale DSMC will perform an interim safety analysis after 15 patients have received at least 1 cycle (28 days) of treatment to determine whether there are any safety issues and to assess whether it is safe to continue enrollment. Enrollment will be suspended until approval is granted from the DSMC to proceed completion of enrollment.

If a new unexpected toxicity is identified from the above safety review, or if the periodic review of all the adverse events and laboratory data raises safety concerns, it may result in revision of the consent form, amendment of the protocol and/or suspension of the protocol.

APPROVED BY THE YALE UNIVERSITY IRB 2/12/2020

Clinical Study Protocol 11/11/19 – version 3.1
TMZ + Olaparib for MGMT Hypermethylated Colorectal Cancer

APPROVED BY THE YALE UNIVERSITY IRB 2/12/2020

10. REFERENCES

FDA Guidance for Industry (issued July 2009) 'Drug-induced liver injury: Premarketing clinical evaluation'

Chan et al 2002
Chan KYK, Ozcelik H, Cheung ANY, Ngan HYS, Khoo US. Epigenetic factors controlling the BRCA1 and BRCA2 genes in sporadic ovarian cancer. Cancer Res 2002:62:4151-4156.

Cronin et al 2018
Cronin, K. A., A. J. Lake, S. Scott, R. L. Sherman, A. M. Noone, N. Howlader, S. J. Henley, R. N. Anderson, A. U. Firth, J. Ma, B. A. Kohler and A. Jemal (2018). "Annual Report to the Nation on the Status of Cancer, part I: National cancer statistics." Cancer **124**(13): 2785-2800.

Fong et al 2009
Fong PC, Boss DS, Yap TA, Tutt A, Wu P, Mergui-Roelvink M, et al. Inhibition of poly(ADP-ribose) polymerase in tumors from *BRCA* mutation carriers. N Engl J Med. 2009;361(2):123-34.

Fulton et al 2017
Fulton, B., S. C. Short, A. James, S. Nowicki, C. McBain, S. Jefferies, C. Kelly, J. Stobo, A. Morris, A. Williamson and A. J. Chalmers (2017). "PARADIGM-2: Two parallel phase I studies of olaparib and radiotherapy or olaparib and radiotherapy plus temozolomide in patients with newly diagnosed glioblastoma, with treatment stratified by MGMT status." Clinical and translational radiation oncology **8**: 12-16.

Halford et al 2017
Halford, S. E. R., G. Cruickshank, L. Dunn, S. Erridge, L. Godfrey, C. Herbert, S. Jefferies, J. S. Lopez, C. McBain, M. Pittman, R. Sleigh, C. Watts, M. F. Webster-Smith and A. J. Chalmers (2017). "Results of the OPARATIC trial: A phase I dose escalation study of olaparib in combination with temozolomide (TMZ) in patients with relapsed glioblastoma (GBM)." Journal of Clinical Oncology **35**(15_suppl): 2022-2022.

Hay et al 2009
Hay T, Matthews JR, Pietzka L, Lau A, Cranston A, Nygren AO, et al. Poly(ADP-ribose) polymerase-1 inhibitor treatment regresses autochthonous Brca2/p53-mutant mammary tumors in vivo and delays tumor relapse in combination with carboplatin. Cancer Res. 2009;69(9):3850-5.

Helleday 2011
Helleday T. The underlying mechanism for the PARP and *BRCA* synthetic lethality: Clearing up the misunderstandings. Molecular Oncology 2011; 5: 387-393.

Murai et al 2012
Murai J, Huang SN, Das BB, Renaud A, Zhnag, Y, Doroshow, JH, et al. Trapping of PARP1 and PARP2 by Clinical PARP Inhibitors. AACR; 72(21); 5588–99.

Muratore et al 2007
Muratore, A., D. Zorzi, H. Bouzari, M. Amisano, P. Massucco, E. Sperti and L. Capussotti (2007). "Asymptomatic Colorectal Cancer with Un-Resectable Liver Metastases: Immediate Colorectal Resection or Up-Front Systemic Chemotherapy?" Annals of Surgical Oncology **14**(2): 766-770.

Narod and Foulkes 2004
Narod SA and Foulkes WD. BRCA1 and BRCA2: 1994 and beyond. Nat Rev Cancer 2004;4(9):665-76.

Nguewa et al 2005
Nguewa PA, Fuertes MA, Valladares B, Alonso C, Perez JM. Poly(ADP-ribose) polymerases: homology, structural domains and functions. Novel therapeutic applications. Progr Biophys Mol Biol 2005;88:143-172.

Rottenberg et al 2008
Rottenberg S, Jaspers JE, Kersbergen A, van der Burg E, Nygren AO, Zander SA, et al. High sensitivity of *BRCA1*-deficient mammary tumors to the PARP inhibitor AZD2281 alone and in combination with platinum drugs. Proc Natl Acad Sci. 2008;105(44):17079-84.

Virag and Szabo 2002
Virag L and Szabo C. The therapeutic potential of poly(ADP-Ribose) polymerase inhibitors. Pharmacol Rev 2002;54(3):375-429.

Bae, S. I. (2002). "Inactivation of O6-methylguanine-DNA methyltransferase by promoter CpG island hypermethylation in gastric cancers." British journal of cancer **86**(12): 1888-1892.

Brandes, A. A. (2006). "Correlations Between O6-Methylguanine DNA Methyltransferase Promoter Methylation Status, 1p and 19q Deletions, and Response to Temozolomide in Anaplastic and Recurrent Oligodendroglioma: A Prospective GICNO Study." Journal of clinical oncology **24**(29): 4746-4753.

Chinot, O. L. (2007). "Correlation Between O -Methylguanine-DNA Methyltransferase and Survival in Inoperable Newly Diagnosed Glioblastoma Patients Treated With Neoadjuvant Temozolomide." Journal of clinical oncology **25**(12): 1470-1475.

Clark, O. (2016). "Molecular Pathways: Isocitrate Dehydrogenase Mutations in Cancer." Clinical cancer research **22**(8): 1837-1842.

Esteller, M. (1999). "Inactivation of the DNA repair gene O6-methylguanine-DNA methyltransferase by promoter hypermethylation is a common event in primary human neoplasia." Cancer research (Chicago, Ill.) **59**(4): 793.

Hegi, M. E. (2005). "Gene Silencing and Benefit from Temozolomide in Glioblastoma." The New England journal of medicine **352**(10): 997-1003.

APPROVED BY THE YALE UNIVERSITY IRB 2/12/2020

Konduri, S. D. (2009). "Blockade of MGMT expression by O6 benzyl guanine leads to inhibition of pancreatic cancer growth and induction of apoptosis." Clinical cancer research **15**(19): 6087-6095.

Kulke, M. H. (2009). "O6-methylguanine DNA methyltransferase deficiency and response to temozolomide-based therapy in patients with neuroendocrine tumors." Clinical cancer research **15**(1): 338-345.

Noushmehr, H. (2010). "Identification of a CpG island methylator phenotype that defines a distinct subgroup of glioma." Cancer cell **17**(5): 510-522.

Thomas, A. (2017). "Temozolomide in the Era of Precision Medicine." Cancer research (Chicago, Ill.) **77**(4): 823-826.

Turcan, S. (2012). "IDH1 mutation is sufficient to establish the glioma hypermethylator phenotype." Nature (London) **483**(7390): 479-483.

van den Bent, M. J. (2013). "MGMT-STP27 methylation status as predictive marker for response to PCV in anaplastic Oligodendrogliomas and Oligoastrocytomas. A report from EORTC study 26951." Clinical cancer research **19**(19): 5513-5522.

Walter, T. (2015). "O6-Methylguanine-DNA methyltransferase status in neuroendocrine tumours: prognostic relevance and association with response to alkylating agents." British journal of cancer **112**(3): 523-531.

Amatu, A., L. Barault, C. Moutinho, A. Cassingena, K. Bencardino, S. Ghezzi, L. Palmeri, E. Bonazzina, F. Tosi, R. Ricotta, T. Cipani, P. Crivori, R. Gatto, G. Chirico, G. Marrapese, M. Truini, A. Bardelli, M. Esteller, F. Di Nicolantonio, A. Sartore-Bianchi and S. Siena (2016). "Tumor MGMT promoter hypermethylation changes over time limit temozolomide efficacy in a phase II trial for metastatic colorectal cancer." Ann Oncol **27**(6): 1062-1067.

Amatu, A., A. Sartore-Bianchi, C. Moutinho, A. Belotti, K. Bencardino, G. Chirico, A. Cassingena, F. Rusconi, A. Esposito, M. Nichelatti, M. Esteller and S. Siena (2013). "Promoter CpG island hypermethylation of the DNA repair enzyme MGMT predicts clinical response to dacarbazine in a phase II study for metastatic colorectal cancer." Clin Cancer Res **19**(8): 2265-2272.

Bae, S. I. (2002). "Inactivation of O6-methylguanine-DNA methyltransferase by promoter CpG island hypermethylation in gastric cancers." British journal of cancer **86**(12): 1888-1892.

APPROVED BY THE YALE UNIVERSITY IRB 2/12/2020

APPROVED BY THE YALE UNIVERSITY IRB 2/12/2020

Barault, L. (2015). "Digital PCR quantification of methylation refines prediction of clinical benefit from alkylating agents in glioblastoma and metastatic colorectal cancer." Annals of oncology **26**(9): 1994-1999.

Barvaux, V. A. (2004). "Sensitization of a human ovarian cancer cell line to temozolomide by simultaneous attenuation of the Bcl-2 antiapoptotic protein and DNA repair by O6-alkylguanine-DNA alkyltransferase." Molecular cancer therapeutics **3**(10): 1215.

Borger, D. R., K. K. Tanabe, K. C. Fan, H. U. Lopez, V. R. Fantin, K. S. Straley, D. P. Schenkein, A. F. Hezel, M. Ancukiewicz, H. M. Liebman, E. L. Kwak, J. W. Clark, D. P. Ryan, V. Deshpande, D. Dias-Santagata, L. W. Ellisen, A. X. Zhu and A. J. Iafrate (2012). "Frequent Mutation of Isocitrate Dehydrogenase (IDH)1 and IDH2 in Cholangiocarcinoma Identified Through Broad-Based Tumor Genotyping." The Oncologist **17**(1): 72-79.

Boulton, S., L. C. Pemberton, J. K. Porteous, N. J. Curtin, R. J. Griffin, B. T. Golding and B. W. Durkacz (1995). "Potentiation of temozolomide-induced cytotoxicity: a comparative study of the biological effects of poly(ADP-ribose) polymerase inhibitors." British Journal Of Cancer **72**: 849.

Brandes, A. A. (2006). "Correlations Between O6-Methylguanine DNA Methyltransferase Promoter Methylation Status, 1p and 19q Deletions, and Response to Temozolomide in Anaplastic and Recurrent Oligodendroglioma: A Prospective GICNO Study." Journal of clinical oncology **24**(29): 4746-4753.

Calabrese, C. R., R. Almassy, S. Barton, M. A. Batey, A. H. Calvert, S. Canan-Koch, B. W. Durkacz, Z. Hostomsky, R. A. Kumpf, S. Kyle, J. Li, K. Maegley, D. R. Newell, E. Notarianni, I. J. Stratford, D. Skalitzky, H. D. Thomas, L.-Z. Wang, S. E. Webber, K. J. Williams and N. J. Curtin (2004). "Anticancer Chemosensitization and Radiosensitization by the Novel Poly(ADP-ribose) Polymerase-1 Inhibitor AG14361." JNCI: Journal of the National Cancer Institute **96**(1): 56-67.

Calegari, M. A., A. Inno, S. Monterisi, A. Orlandi, D. Santini, M. Basso, A. Cassano, M. Martini, T. Cenci, I. de Pascalis, F. Camarda, B. Barbaro, L. M. Larocca, S. Gori, G. Tonini and C. Barone (2017). "A phase 2 study of temozolomide in pretreated metastatic colorectal cancer with MGMT promoter methylation." Br J Cancer **116**(10): 1279-1286.

Ceccarelli, M., F. P. Barthel, T. M. Malta, T. S. Sabedot, S. R. Salama, B. A. Murray, O. Morozova, Y. Newton, A. Radenbaugh, S. M. Pagnotta, S. Anjum, J. Wang, G. Manyam, P. Zoppoli, S. Ling, A. A. Rao, M. Grifford, A. D. Cherniack, H. Zhang, L. Poisson, C. G. Carlotti, Jr., D. P. Tirapelli, A. Rao, T. Mikkelsen, C. C. Lau, W. K. Yung, R. Rabadan, J. Huse, D. J. Brat, N. L. Lehman, J. S. Barnholtz-Sloan, S. Zheng, K. Hess, G. Rao, M. Meyerson, R. Beroukhim, L. Cooper, R. Akbani, M. Wrensch, D. Haussler, K. D. Aldape, P. W. Laird, D. H. Gutmann, T. R. Network, H. Noushmehr, A. Iavarone and R. G. Verhaak (2016). "Molecular Profiling Reveals Biologically Discrete Subsets and Pathways of Progression in Diffuse Glioma." Cell **164**(3): 550-563.

APPROVED BY THE YALE UNIVERSITY IRB 2/12/2020

APPROVED BY THE YALE UNIVERSITY IRB 2/12/2020

Chen, F., K. Bian, Q. Tang, B. I. Fedeles, V. Singh, Z. T. Humulock, J. M. Essigmann and D. Li (2017). "Oncometabolites d- and l-2-Hydroxyglutarate Inhibit the AlkB Family DNA Repair Enzymes under Physiological Conditions." Chem Res Toxicol **30**(4): 1102-1110.

Chinot, O. L. (2007). "Correlation Between O -Methylguanine-DNA Methyltransferase and Survival in Inoperable Newly Diagnosed Glioblastoma Patients Treated With Neoadjuvant Temozolomide." Journal of clinical oncology **25**(12): 1470-1475.

Cimprich, K. A. and D. Cortez (2008). "ATR: an essential regulator of genome integrity." Nature Reviews Molecular Cell Biology **9**: 616.

Clark, O. (2016). "Molecular Pathways: Isocitrate Dehydrogenase Mutations in Cancer." Clinical cancer research **22**(8): 1837-1842.

Curtin, K., M. L. Slattery and W. S. Samowitz (2011). "CpG island methylation in colorectal cancer: past, present and future." Pathology research international **2011**: 902674-902674.

Curtin, N. J., L.-Z. Wang, A. Yiakouvaki, S. Kyle, C. A. Arris, S. Canan-Koch, S. E. Webber, B. W. Durkacz, H. A. Calvert, Z. Hostomsky and D. R. Newell (2004). "Novel Poly(ADP-ribose) Polymerase-1 Inhibitor, AG14361, Restores Sensitivity to Temozolomide in Mismatch Repair-Deficient Cells." Clinical Cancer Research **10**(3): 881-889.

Delaney, C. A. (2000). "Potentiation of temozolomide and topotecan growth inhibition and cytotoxicity by novel poly(adenosine diphosphoribose) polymerase inhibitors in a panel of human tumor cell lines." Clinical cancer research **6**(7): 2860.

Esteller, M. (1999). "Inactivation of the DNA repair gene O6-methylguanine-DNA methyltransferase by promoter hypermethylation is a common event in primary human neoplasia." Cancer research (Chicago, Ill.) **59**(4): 793.

Esteller, M. and J. G. Herman (2004). "Generating mutations but providing chemosensitivity: the role of O6-methylguanine DNA methyltransferase in human cancer." Oncogene **23**: 1.

Esteller, M., M. Toyota, M. Sanchez-Cespedes, G. Capella, M. A. Peinado, D. N. Watkins, J.-P. J. Issa, D. Sidransky, S. B. Baylin and J. G. Herman (2000). "Inactivation of the DNA Repair Gene O⁶-Methylguanine-DNA Methyltransferase by Promoter Hypermethylation Is Associated with G to A Mutations in K-ras in Colorectal Tumorigenesis." Cancer Research **60**(9): 2368.

Farago, A. F., B. J. Drapkin, A. Charles, B. Y. Yeap, R. S. Heist, C. G. Azzoli, D. M. Jackman, J. P. Marcoux, D. A. Barbie, D. T. Myers, S. Phat, J. Zhong, J. B. Grinnell, L. V. Sequist, M. Mino-Kenudson, S. Maheswaran, D. A. Haber, A. N. Hata, N. J. Dyson and A. T. Shaw (2018). "Safety and efficacy of combination olaparib (O) and temozolomide (T) in small cell lung cancer (SCLC)." Journal of Clinical Oncology **36**(15_suppl): 8571-8571.

APPROVED BY THE YALE UNIVERSITY IRB 2/12/2020

Figueroa, M. E. (2010). "Leukemic IDH1 and IDH2 mutations result in a hypermethylation phenotype, disrupt TET2 function, and impair hematopoietic differentiation." Cancer cell **18**(6): 553-567.

Fulton, B., S. C. Short, A. James, S. Nowicki, C. McBain, S. Jefferies, C. Kelly, J. Stobo, A. Morris, A. Williamson and A. J. Chalmers (2017). "PARADIGM-2: Two parallel phase I studies of olaparib and radiotherapy or olaparib and radiotherapy plus temozolomide in patients with newly diagnosed glioblastoma, with treatment stratified by MGMT status." Clinical and translational radiation oncology **8**: 12-16.

Gill, S. J., J. Travers, I. Pshenichnaya, F. A. Kogera, S. Barthorpe, T. Mironenko, L. Richardson, C. H. Benes, M. R. Stratton, U. McDermott, S. P. Jackson and M. J. Garnett (2015). "Combinations of PARP Inhibitors with Temozolomide Drive PARP1 Trapping and Apoptosis in Ewing's Sarcoma." PLoS ONE **10**(10): e0140988.

Hegi, M. E. (2005). "Gene Silencing and Benefit from Temozolomide in Glioblastoma." The New England journal of medicine **352**(10): 997-1003.

Hermisson, M. (2006). "O6-methylguanine DNA methyltransferase and p53 status predict temozolomide sensitivity in human malignant glioma cells." Journal of neurochemistry **96**(3): 766-776.

Hochhauser, D., R. Glynne-Jones, V. Potter, C. Gravalos, T. J. Doyle, K. Pathiraja, Q. Zhang, L. Zhang and E. A. Sausville (2013). "A phase II study of temozolomide in patients with advanced aerodigestive tract and colorectal cancers and methylation of the O6-methylguanine-DNA methyltransferase promoter." Mol Cancer Ther **12**(5): 809-818.

Hughes, L. A. E., V. Melotte, J. de Schrijver, M. de Maat, V. T. H. B. M. Smit, J. V. M. G. Bovée, P. J. French, P. A. van den Brandt, L. J. Schouten, T. de Meyer, W. van Criekinge, N. Ahuja, J. G. Herman, M. P. Weijenberg and M. van Engeland (2013). "The CpG Island Methylator Phenotype: What's in a Name?" Cancer Research **73**(19): 5858.

Jagtap, P. and C. Szabó (2005). "Poly(ADP-ribose) polymerase and the therapeutic effects of its inhibitors." Nature Reviews Drug Discovery **4**: 421.

Javle, M., T. Bekaii - Saab, A. Jain, Y. Wang, K. Kelley Robin, K. Wang, C. Kang Hyunseon, D. Catenacci, S. Ali, S. Krishnan, D. Ahn, G. Bocobo Andrea, M. Zuo, A. Kaseb, V. Miller, J. Stephens Philip, F. Meric - Bernstam, R. Shroff and J. Ross (2016). "Biliary cancer: Utility of next - generation sequencing for clinical management." Cancer **122**(24): 3838-3847.

Konduri, S. D. (2009). "Blockade of MGMT expression by O6 benzyl guanine leads to inhibition of pancreatic cancer growth and induction of apoptosis." Clinical cancer research **15**(19): 6087-6095.

APPROVED BY THE YALE UNIVERSITY IRB 2/12/2020

Kulke, M. H. (2009). "O6-methylguanine DNA methyltransferase deficiency and response to temozolomide-based therapy in patients with neuroendocrine tumors." Clinical cancer research **15**(1): 338-345.

Lavin, M. F. (2008). "Ataxia-telangiectasia: from a rare disorder to a paradigm for cell signalling and cancer." Nature Reviews Molecular Cell Biology **9**: 759.

Le, D. T., J. N. Durham, K. N. Smith, H. Wang, B. R. Bartlett, L. K. Aulakh, S. Lu, H. Kemberling, C. Wilt, B. S. Luber, F. Wong, N. S. Azad, A. A. Rucki, D. Laheru, R. Donehower, A. Zaheer, G. A. Fisher, T. S. Crocenzi, J. J. Lee, T. F. Greten, A. G. Duffy, K. K. Ciombor, A. D. Eyring, B. H. Lam, A. Joe, S. P. Kang, M. Holdhoff, L. Danilova, L. Cope, C. Meyer, S. Zhou, R. M. Goldberg, D. K. Armstrong, K. M. Bever, A. N. Fader, J. Taube, F. Housseau, D. Spetzler, N. Xiao, D. M. Pardoll, N. Papadopoulos, K. W. Kinzler, J. R. Eshleman, B. Vogelstein, R. A. Anders and L. A. Diaz (2017). "Mismatch repair deficiency predicts response of solid tumors to PD-1 blockade." Science **357**(6349): 409-413.

Le, D. T., J. N. Uram, H. Wang, B. R. Bartlett, H. Kemberling, A. D. Eyring, A. D. Skora, B. S. Luber, N. S. Azad, D. Laheru, B. Biedrzycki, R. C. Donehower, A. Zaheer, G. A. Fisher, T. S. Crocenzi, J. J. Lee, S. M. Duffy, R. M. Goldberg, A. de la Chapelle, M. Koshiji, F. Bhaijee, T. Huebner, R. H. Hruban, L. D. Wood, N. Cuka, D. M. Pardoll, N. Papadopoulos, K. W. Kinzler, S. Zhou, T. C. Cornish, J. M. Taube, R. A. Anders, J. R. Eshleman, B. Vogelstein and L. A. J. Diaz (2015). "PD-1 Blockade in Tumors with Mismatch-Repair Deficiency." New England Journal of Medicine **372**(26): 2509-2520.

Letouze, E., C. Martinelli, C. Loriot, N. Burnichon, N. Abermil, C. Ottolenghi, M. Janin, M. Menara, A. T. Nguyen, P. Benit, A. Buffet, C. Marcaillou, J. Bertherat, L. Amar, P. Rustin, A. De Reynies, A. P. Gimenez-Roqueplo and J. Favier (2013). "SDH mutations establish a hypermethylator phenotype in paraganglioma." Cancer Cell **23**(6): 739-752.

Liu, L., L. Yan, J. R. Donze and S. L. Gerson (2003). "Blockage of abasic site repair enhances antitumor efficacy of 1,3-bis-(2-chloroethyl)-1-nitrosourea in colon tumor xenografts." Molecular Cancer Therapeutics **2**(10): 1061-1066.

Loupakis et al 2014

Loupakis, F., C. Cremolini, G. Masi, S. Lonardi, V. Zagonel, L. Salvatore, E. Cortesi, G. Tomasello, M. Ronzoni, R. Spadi, A. Zaniboni, G. Tonini, A. Buonadonna, D. Amoroso, S. Chiara, C. Carlomagno, C. Boni, G. Allegrini, L. Boni and A. Falcone (2014). "Initial Therapy with FOLFOXIRI and Bevacizumab for Metastatic Colorectal Cancer." New England Journal of Medicine **371**(17): 1609-1618.

Luo, M. (2004). "Inhibition of the human apurinic/apyrimidinic endonuclease (APE1) repair activity and sensitization of breast cancer cells to DNA alkylating agents with lucanthone." Anticancer research **24**(4): 2127.

APPROVED BY THE YALE UNIVERSITY IRB 2/12/2020

APPROVED BY THE YALE UNIVERSITY IRB 2/12/2020

Morano, F., S. Corallo, M. Niger, L. Barault, M. Milione, R. Berenato, R. Moretto, G. Randon, M. Antista, A. Belfiore, A. Raimondi, F. Nichetti, A. Martinetti, L. Battaglia, F. Perrone, G. Pruneri, A. Falcone, M. Di Bartolomeo, F. de Braud, F. Di Nicolantonio, C. Cremolini and F. Pietrantonio (2018). "Temozolomide and irinotecan (TEMIRI regimen) as salvage treatment of irinotecan-sensitive advanced colorectal cancer patients bearing MGMT methylation." Annals of Oncology: mdy197-mdy197.

Murai, J., S.-y. N. Huang, B. B. Das, A. Renaud, Y. Zhang, J. H. Doroshow, J. Ji, S. Takeda and Y. Pommier (2012). "Trapping of PARP1 and PARP2 by Clinical PARP Inhibitors." Cancer Research **72**(21): 5588-5599.

Murai, J., Y. Zhang, J. Morris, J. Ji, S. Takeda, J. H. Doroshow and Y. Pommier (2014). "Rationale for Poly(ADP-ribose) Polymerase (PARP) Inhibitors in Combination Therapy with Camptothecins or Temozolomide Based on PARP Trapping versus Catalytic Inhibition." Journal of Pharmacology and Experimental Therapeutics **349**(3): 408-416.
Nagel, Z. D., G. J. Kitange, S. K. Gupta, B. A. Joughin, I. A. Chaim, P. Mazzucato, D. A. Lauffenburger, J. N. Sarkaria and L. D. Samson (2017). "DNA Repair Capacity in Multiple Pathways Predicts Chemoresistance in Glioblastoma Multiforme." Cancer Research **77**(1): 198-206.

Noushmehr, H. (2010). "Identification of a CpG island methylator phenotype that defines a distinct subgroup of glioma." Cancer cell **17**(5): 510-522.

Ohmoto, A. and S. Yachida (2017). "Current status of poly(ADP-ribose) polymerase inhibitors and future directions." OncoTargets and therapy **10**: 5195-5208.

Palma, J. P., Y.-C. Wang, L. E. Rodriguez, D. Montgomery, P. A. Ellis, G. Bukofzer, A. Niquette, X. Liu, Y. Shi, L. Lasko, G.-D. Zhu, T. D. Penning, V. L. Giranda, S. H. Rosenberg, D. J. Frost and C. K. Donawho (2009). "ABT-888 Confers Broad In vivo Activity in Combination with Temozolomide in Diverse Tumors." Clinical Cancer Research **15**(23): 7277-7290.

Pietrantonio, F., F. de Braud, M. Milione, C. Maggi, R. Iacovelli, K. F. Dotti, F. Perrone, E. Tamborini, M. Caporale, R. Berenato, G. Leone, A. Pellegrinelli, I. Bossi, F. Festinese, S. Federici and M. Di Bartolomeo (2016). "Dose-Dense Temozolomide in Patients with MGMT-Silenced Chemorefractory Colorectal Cancer." Target Oncol **11**(3): 337-343.

Pietrantonio, F., F. Perrone, F. de Braud, A. Castano, C. Maggi, I. Bossi, A. Gevorgyan, P. Biondani, M. Pacifici, A. Busico, M. Gariboldi, F. Festinese, E. Tamborini and M. Di Bartolomeo (2014). "Activity of temozolomide in patients with advanced chemorefractory colorectal cancer and MGMT promoter methylation." Ann Oncol **25**(2): 404-408.

Pishvaian, M. J., R. S. Slack, W. Jiang, A. R. He, J. J. Hwang, A. Hankin, K. Dorsch - Vogel, D. Kukadiya, L. M. Weiner, J. L. Marshall and J. R. Brody "A phase 2 study of the PARP inhibitor

APPROVED BY THE YALE UNIVERSITY IRB 2/12/2020

APPROVED BY THE YALE UNIVERSITY IRB 2/12/2020

veliparib plus temozolomide in patients with heavily pretreated metastatic colorectal cancer." Cancer **0**(0).
Pommier, Y., M. J. O'Connor and J. de Bono (2016). "Laying a trap to kill cancer cells: PARP inhibitors and their mechanisms of action." Science Translational Medicine **8**(362): 362ps317-362ps317.

Ryan, D. P. (2014). "Pancreatic Adenocarcinoma." The New England journal of medicine **371**(11): 1039-1049.

Sartore-Bianchi, A., F. Pietrantonio, A. Amatu, M. Milione, A. Cassingena, S. Ghezzi, M. Caporale, R. Berenato, C. Falcomata, A. Pellegrinelli, A. Bardelli, M. Nichelatti, F. Tosi, F. De Braud, F. Di Nicolantonio, L. Barault and S. Siena (2017). "Digital PCR assessment of MGMT promoter methylation coupled with reduced protein expression optimises prediction of response to alkylating agents in metastatic colorectal cancer patients." Eur J Cancer **71**: 43-50.

Shacham-Shmueli, E., A. Beny, R. Geva, A. Blachar, A. Figer and D. Aderka (2011). "Response to Temozolomide in Patients With Metastatic Colorectal Cancer With Loss of MGMT Expression: A New Approach in the Era of Personalized Medicine?" Journal of Clinical Oncology **29**(10): e262-e265.

Shen, J., Y. Peng, L. Wei, W. Zhang, L. Yang, L. Lan, P. Kapoor, Z. Ju, Q. Mo, M. Shih Ie, I. P. Uray, X. Wu, P. H. Brown, X. Shen, G. B. Mills and G. Peng (2015). "ARID1A Deficiency Impairs the DNA Damage Checkpoint and Sensitizes Cells to PARP Inhibitors." Cancer Discov **5**(7): 752-767.

Smith, M. A., C. P. Reynolds, M. H. Kang, E. A. Kolb, R. Gorlick, H. Carol, R. B. Lock, S. T. Keir, J. M. Maris, C. A. Billups, D. Lyalin, R. T. Kurmasheva and P. J. Houghton (2015). "Synergistic Activity of PARP Inhibition by Talazoparib (BMN 673) with Temozolomide in Pediatric Cancer Models in the Pediatric Preclinical Testing Program." Clinical Cancer Research **21**(4): 819-832.

Suehiro, Y., C. W. Wong, L. R. Chirieac, Y. Kondo, L. Shen, C. R. Webb, Y. W. Chan, A. S. Y. Chan, T. L. Chan, T.-T. Wu, A. Rashid, Y. Hamanaka, Y. Hinoda, R. L. Shannon, X. Wang, J. Morris, J.-P. J. Issa, S. T. Yuen, S. Y. Leung and S. R. Hamilton (2008). "Epigenetic-Genetic Interactions in the APC/WNT, RAS/RAF, and P53 Pathways in Colorectal Carcinoma." Clinical Cancer Research **14**(9): 2560-2569.

Tentori, L., C. Leonetti, M. Scarsella, G. d'Amati, M. Vergati, I. Portarena, W. Xu, V. Kalish, G. Zupi, J. Zhang and G. Graziani (2003). "Systemic Administration of GPI 15427, a Novel Poly(ADP-Ribose) Polymerase-1 Inhibitor, Increases the Antitumor Activity of Temozolomide against Intracranial Melanoma, Glioma, Lymphoma." Clinical Cancer Research **9**(14): 5370-5379.

Tentori, L., I. Portarena, E. Bonmassar and G. Graziani (2001). "Combined effects of adenovirus-mediated wild-type p53 transduction, temozolomide and poly (ADP-ribose)

APPROVED BY THE YALE UNIVERSITY IRB 2/12/2020

polymerase inhibitor in mismatch repair deficient and non-proliferating tumor cells." Cell Death And Differentiation **8**: 457.

Tentori, L., I. Portarena, P. Vernole, P. De Fabritiis, R. Madaio, A. Balduzzi, R. Roy, E. Bonmassar and G. Graziani (2001). "Effects of single or split exposure of leukemic cells to temozolomide, combined with poly(ADP-ribose) polymerase inhibitors on cell growth, chromosomal aberrations and base excision repair components." Cancer Chemotherapy and Pharmacology **47**(4): 361-369.

Thomas, A. (2017). "Temozolomide in the Era of Precision Medicine." Cancer research (Chicago, Ill.) **77**(4): 823-826.

Thomas, A., M. Tanaka, J. Trepel, W. C. Reinhold, V. N. Rajapakse and Y. Pommier (2017). "Temozolomide in the Era of Precision Medicine." Cancer Research **77**(4): 823-826.

Trivedi, R. N. (2005). "The role of base excision repair in the sensitivity and resistance to temozolomide-mediated cell death." Cancer research (Chicago, Ill.) **65**(14): 6394-6400.
Turcan, S. (2012). "IDH1 mutation is sufficient to establish the glioma hypermethylator phenotype." Nature (London) **483**(7390): 479-483.

van den Bent, M. J. (2013). "MGMT-STP27 methylation status as predictive marker for response to PCV in anaplastic Oligodendrogliomas and Oligoastrocytomas. A report from EORTC study 26951." Clinical cancer research **19**(19): 5513-5522.

Walter, T. (2015). "O6-Methylguanine-DNA methyltransferase status in neuroendocrine tumours: prognostic relevance and association with response to alkylating agents." British journal of cancer **112**(3): 523-531.

Wang, P., J. Wu, S. Ma, L. Zhang, J. Yao, K. A. Hoadley, M. D. Wilkerson, C. M. Perou, K. L. Guan, D. Ye and Y. Xiong (2015). "Oncometabolite D-2-Hydroxyglutarate Inhibits ALKBH DNA Repair Enzymes and Sensitizes IDH Mutant Cells to Alkylating Agents." Cell Rep **13**(11): 2353-2361.

Williamson, C. T., R. Miller, H. N. Pemberton, S. E. Jones, J. Campbell, A. Konde, N. Badham, R. Rafiq, R. Brough, A. Gulati, C. J. Ryan, J. Francis, P. B. Vermulen, A. R. Reynolds, P. M. Reaper, J. R. Pollard, A. Ashworth and C. J. Lord (2016). "ATR inhibitors as a synthetic lethal therapy for tumours deficient in ARID1A." Nat Commun **7**: 13837.

Yan, L., A. Bulgar, Y. Miao, V. Mahajan, J. R. Donze, S. L. Gerson and L. Liu (2007). "Combined Treatment with Temozolomide and Methoxyamine: Blocking Apurininc/Pyrimidinic Site Repair Coupled with Targeting Topoisomerase IIα." Clinical Cancer Research **13**(5): 1532-1539.

Yang, Y.-G., U. Cortes, S. Patnaik, M. Jasin and Z.-Q. Wang (2004). "Ablation of PARP-1 does not interfere with the repair of DNA double-strand breaks, but compromises the reactivation of stalled replication forks." Oncogene **23**: 3872.

APPROVED BY THE YALE UNIVERSITY IRB 2/12/2020

11. SUPPORTING DOCUMENTATION AND OPERATIONAL CONSIDERATIONS

APPROVED BY THE YALE UNIVERSITY IRB 2/12/2020

APPROVED BY THE YALE UNIVERSITY IRB 2/12/2020

Appendix A Actions required in cases of increases in liver biochemistry and evaluation of Hy's Law

A 1 Introduction

This Appendix describes the process to be followed in order to identify and appropriately report cases of Hy's Law. It is not intended to be a comprehensive guide to the management of elevated liver biochemistries.

During the course of the study the Investigator will remain vigilant for increases in liver biochemistry. The Investigator is responsible for determining whether a patient meets potential Hy's Law (PHL) criteria at any point during the study.

The Investigator participates, together with AstraZeneca clinical project representatives, in review and assessment of cases meeting PHL criteria to agree whether Hy's Law (HL) criteria are met. HL criteria are met if there is no alternative explanation for the elevations in liver biochemistry other than drug induced liver injury (DILI) caused by the investigational medicinal products (IP).

The Investigator is responsible for recording data pertaining to PHL/HL cases and for reporting Adverse Events (AE) and Serious Adverse Events (SAE) according to the outcome of the review and assessment in line with standard safety reporting processes.

A 2 Definitions

Potential Hy's Law (PHL)

Aspartate aminotransferase (AST) or alanine aminotransferase (ALT) ≥ 3× upper limit of normal (ULN) **together with** total bilirubin (TBL) ≥ 2×ULN at any point during the study following the start of study medication irrespective of an increase in alkaline phosphatase (ALP).

Hy's Law (HL)

AST or ALT ≥ 3 × ULN **together with** TBL ≥ 2 ×ULN, where no other reason, other than the IMP, can be found to explain the combination of increases, eg, elevated ALP indicating cholestasis, viral hepatitis, another drug.

For PHL and HL the elevation in transaminases must precede or be coincident with (ie, on the same day) the elevation in TBL, but there is no specified time frame within which the elevations in transaminases and TBL must occur.

A 3 Identification of potential Hy's Law cases

In order to identify cases of PHL it is important to perform a comprehensive review of laboratory data for any patient who meets any of the following identification criteria in isolation or in combination:

- ALT ≥ 3 × ULN

APPROVED BY THE YALE UNIVERSITY IRB 2/12/2020

APPROVED BY THE YALE UNIVERSITY IRB 2/12/2020

- AST ≥ 3 × ULN
- TBL ≥ 2 × ULN

When a patient meets any of the identification criteria, in isolation or in combination, the central laboratory will immediately send an alert to the Principal Investigator.

The Investigator will also remain vigilant for any local laboratory reports where the identification criteria are met without an alternative explanation, where this is the case the Investigator will:

- Notify the AstraZeneca representative
- Request a repeat of the test (new blood draw) by the central laboratory
- Complete the appropriate unscheduled laboratory CRF module(s) with the original local laboratory test result

When the identification criteria are met from central or local laboratory results the Investigator will without delay:

- Determine whether the patient meets PHL criteria (see Appendix A 2 for definition) by reviewing laboratory reports from all previous visits (including both central and local laboratory results)

The Investigator will without delay review each new laboratory report and if the identification criteria are met and there is no alterative explanation the investigator will:

- Notify the AstraZeneca representative
- Determine whether the patient meets PHL criteria (see Appendix A 2 for definition) by reviewing laboratory reports from all previous visits
- Promptly enter the laboratory data into the laboratory CRF

A 4 Follow-up

A 4.1 Potential Hy's Law criteria not met

If the patient does not meet PHL criteria the Investigator will:

- Inform the AstraZeneca representative that the patient has not met PHL criteria.
- Perform follow-up on subsequent laboratory results according to the guidance provided in the Clinical Study Protocol.

A 4.2 Potential Hy's Law criteria met

If the patient does meet PHL criteria the Investigator will:

APPROVED BY THE YALE UNIVERSITY IRB 2/12/2020

Determine whether PHL criteria were met at any study visit prior to starting Study treatment (See Section 8.4 Safety Reporting)

- Notify the AstraZeneca representative who will then inform the central Study Team

The Study Physician contacts the Investigator, to provide guidance, discuss and agree an approach for the study patients' follow-up and the continuous review of data. Subsequent to this contact the Investigator will:

- Monitor the patient until liver biochemistry parameters and appropriate clinical symptoms and signs return to normal or baseline levels, or as long as medically indicated
- Investigate the aetiology of the event and perform diagnostic investigations as discussed with the Study Physician.
- Complete the three Liver CRF Modules as information becomes available
- If at any time (in consultation with the Study Physician the PHL case meets serious criteria, report it as an SAE using standard reporting procedures.

A 5 Review and assessment of potential Hy's Law cases

The instructions in this section should be followed for all cases where PHL criteria are met.

No later than 3 weeks after the biochemistry abnormality was initially detected, the Study Physician contacts the Investigator in order to review available data and agree on whether there is an alternative explanation for meeting PHL criteria other than DILI caused by the IMP. The AstraZeneca Global Clinical Lead or equivalent and Global Safety Physician will also be involved in this review together with other patient matter experts as appropriate.

According to the outcome of the review and assessment, the Investigator will follow the instructions below.

If there is an agreed alternative explanation for the ALT or AST and TBL elevations, a determination of whether the alternative explanation is an AE will be made and subsequently whether the AE meets the criteria for a SAE:

- If the alternative explanation is **not** an AE, record the alternative explanation on the appropriate CRF
- If the alternative explanation is an AE/SAE, record the AE /SAE in the CRF accordingly and follow the AZ standard processes

If it is agreed that there is **no** explanation that would explain the ALT or AST and TBL elevations other than the IMP:

- Report an SAE (report term 'Hy's Law') according to AstraZeneca standard processes.

APPROVED BY THE YALE UNIVERSITY IRB 2/12/2020

- The 'Medically Important' serious criterion should be used if no other serious criteria apply
- As there is no alternative explanation for the HL case, a causality assessment of 'related' should be assigned.

If there is an unavoidable delay of over 3 weeks in obtaining the information necessary to assess whether or not the case meets the criteria for HL, then it is assumed that there is no alternative explanation until such time as an informed decision can be made:

- Report an SAE (report term 'Potential Hy's Law') applying serious criteria and causality assessment as per above
- Continue follow-up and review according to agreed plan. Once the necessary supplementary information is obtained, repeat the review and assessment to determine whether HL criteria are met. Update the SAE report according to the outcome of the review amending the reported term if an alternative explanation for the liver biochemistry elevations is determined.

A 6 Actions required when potential Hy's Law criteria are met before and after starting study treatment

This section is applicable to patients liver metastases who meet PHL criteria on Study treatment having previously met PHL criteria at a study visit prior to starting Study treatment.

At the first on-study treatment occurrence of PHL criteria being met, the Investigator will determine if there has been a significant change in the patients' condition compared with the last visit where PHL criteria were met.

- If there is no significant change, no action is required
- If there is a significant change, notify the AstraZeneca representative, who will inform the central Study Team, then follow the subsequent process described in Appendix **Error! Reference source not found.**.
- A 'significant' change in the patient's condition refers to a clinically relevant change in any of the individual liver biochemistry parameters (ALT, AST or total bilirubin) in isolation or in combination, or a clinically relevant change in associated symptoms. The determination of whether there has been a significant change will be at the discretion of the Investigator, this may be in consultation with the Study Physician if there is any uncertainty.

A 7 Actions required for repeat episodes of potential Hy's Law

This section is applicable when a patient meets PHL criteria on study treatment, and has already met PHL criteria at a previous on study treatment visit.

APPROVED BY THE YALE UNIVERSITY IRB 2/12/2020

APPROVED BY THE YALE UNIVERSITY IRB 2/12/2020

The requirement to conduct follow-up, review, and assessment of a repeat occurrence(s) of PHL is based on the nature of the alternative cause identified for the previous occurrence.

The investigator should determine the cause for the previous occurrence of PHL criteria being met and answer the following question:

Was the alternative cause for the previous occurrence of PHL criteria being met found to be the disease under study (eg, chronic or progressing malignant disease, severe infection or liver disease), or did the subject meet PHL criteria prior to starting study treatment and at first on-study treatment visit, as described in Appendix A 6.

If **No**: Follow the process described in Appendix A 4.1.

If **Yes**: Determine if there has been a significant change in the patient's condition compared with when PHL criteria were previously met.

If there is no significant change, no action is required.

If there is a significant change, follow the process described in Appendix A 4.

A 'significant' change in the patient's condition refers to a clinically relevant change in any of the individual liver biochemistry parameters (ALT, AST or total bilirubin) in isolation or in combination, or a clinically relevant change in associated symptoms. The determination of whether there has been a significant change will be at the discretion of the Investigator; this may be in consultation with the AstraZeneca Physician if there is any uncertainty.

APPROVED BY THE YALE UNIVERSITY IRB 2/12/2020

Appendix B Adverse event definitions and additional safety information

B1 Definition of adverse events

An adverse event is the development of any untoward medical occurrence in a patient or clinical study patient administered a medicinal product and which does not necessarily have a causal relationship with this treatment. An AE can therefore be any unfavourable and unintended sign (e.g. an abnormal laboratory finding), symptom (for example nausea, chest pain), or disease temporally associated with the use of a medicinal product, whether or not considered related to the medicinal product.

The term AE is used to include both serious and non-serious AEs and can include a deterioration of a pre-existing medical occurrence. An AE may occur at any time, including run-in or washout periods, even if no Study treatment has been administered.

B2 Definitions of serious adverse event

A serious adverse event is an AE occurring during any study phase (i.e., run-in, treatment, washout, follow-up), that fulfils one or more of the following criteria:

12. Results in death
13. Is immediately life-threatening
14. Requires in-patient hospitalisation or prolongation of existing hospitalisation
15. Results in persistent or significant disability or incapacity.
16. Is a congenital abnormality or birth defect
17. Is an important medical event that may jeopardise the patient or may require medical treatment to prevent one of the outcomes listed above.

B3 Life threatening

‘Life-threatening’ means that the patient was at immediate risk of death from the AE as it occurred or it is suspected that use or continued use of the product would result in the patient’s death. ‘Life-threatening’ does not mean that had an AE occurred in a more severe form it might have caused death (e.g., hepatitis that resolved without hepatic failure).

B4 Hospitalisation

Outpatient treatment in an emergency room is not in itself a serious AE, although the reasons for it may be (e.g., bronchospasm, laryngeal oedema). Hospital admissions and/or surgical operations planned before or during a study are not considered AEs if the illness or disease existed before the patient was enrolled in the study, provided that it did not deteriorate in an unexpected way during the study.

B5 Important medical event or medical treatment

APPROVED BY THE YALE UNIVERSITY IRB 2/12/2020

APPROVED BY THE YALE UNIVERSITY IRB 2/12/2020

Medical and scientific judgement should be exercised in deciding whether a case is serious in situations where important medical events may not be immediately life threatening or result in death, hospitalisation, disability or incapacity but may jeopardize the patient or may require medical treatment to prevent one or more outcomes listed in the definition of serious. These should usually be considered as serious.

Simply stopping the suspect drug does not mean that it is an important medical event; medical judgement must be used.

18. Angioedema not severe enough to require intubation but requiring iv hydrocortisone treatment

19. Hepatotoxicity caused by paracetamol (acetaminophen) overdose requiring treatment with N-acetylcysteine

20. Intensive treatment in an emergency room or at home for allergic bronchospasm

21. Blood dyscrasias (e.g., neutropenia or anaemia requiring blood transfusion, etc.) or convulsions that do not result in hospitalisation

22. Development of drug dependency or drug abuse

B6 Intensity rating scale:

The grading scales found in the revised National Cancer Institute CTCAE latest version (5.0) will be utilised for all events with an assigned CTCAE grading. For those events without assigned CTCAE grades, the recommendation in the CTCAE criteria that converts mild, moderate and severe events into CTCAE grades should be used. A copy of the CTCAE can be downloaded from the Cancer Therapy Evaluation Program website (http://ctep.cancer.gov). The applicable version of CTCAE should be described clearly.

For each episode of an adverse event, all changes to the CTCAE grade attained as well as the highest attained CTC grade should be reported.

It is important to distinguish between serious and severe AEs. Severity is a measure of intensity whereas seriousness is defined by the criteria in Appendix B2. An AE of severe intensity need not necessarily be considered serious. For example, nausea that persists for several hours may be considered severe nausea, but not a SAE unless it meets the criteria shown in Appendix B2. On the other hand, a stroke that results in only a limited degree of disability may be considered a mild stroke but would be a SAE when it satisfies the criteria shown in Appendix B2.

B7 A Guide to Interpreting the Causality Question

When making an assessment of causality consider the following factors when deciding if there is a ‘reasonable possibility’ that an AE may have been caused by the drug.

APPROVED BY THE YALE UNIVERSITY IRB 2/12/2020

23. Time Course. Exposure to suspect drug. Has the patient actually received the suspect drug? Did the AE occur in a reasonable temporal relationship to the administration of the suspect drug?

24. Consistency with known drug profile. Was the AE consistent with the previous knowledge of the suspect drug (pharmacology and toxicology) or drugs of the same pharmacological class? Or could the AE be anticipated from its pharmacological properties?

25. De-challenge experience. Did the AE resolve or improve on stopping or reducing the dose of the suspect drug?

26. No alternative cause. The AE cannot be reasonably explained by another aetiology such as the underlying disease, other drugs, other host or environmental factors.

27. Re-challenge experience. Did the AE reoccur if the suspected drug was reintroduced after having been stopped? AstraZeneca would not normally recommend or support a re-challenge.

28. Laboratory tests. A specific laboratory investigation (if performed) has confirmed the relationship.

In difficult cases, other factors could be considered such as:

29. Is this a recognized feature of overdose of the drug?

30. Is there a known mechanism?

Causality of ‘related’ is made if following a review of the relevant data, there is evidence for a ‘reasonable possibility’ of a causal relationship for the individual case. The expression ‘reasonable possibility’ of a causal relationship is meant to convey, in general, that there are facts (evidence) or arguments to suggest a causal relationship.

The causality assessment is performed based on the available data including enough information to make an informed judgment. With limited or insufficient information in the case, it is likely that the event(s) will be assessed as ‘not related’.

Causal relationship in cases where the disease under study has deteriorated due to lack of effect should be classified as no reasonable possibility.

B8 Medication Error

For the purposes of this clinical study a medication error is an unintended failure or mistake in the treatment process for an AstraZeneca study drug that either causes harm to the participant or has the potential to cause harm to the participant.

A medication error is not lack of efficacy of the drug, but rather a human or process related failure while the drug is in control of the study site staff or participant.

APPROVED BY THE YALE UNIVERSITY IRB 2/12/2020

Medication error includes situations where an error.

31. occurred

32. was identified and intercepted before the participant received the drug

33. did not occur, but circumstances were recognize that could have led to an error

Examples of events to be reported in clinical studies as medication errors:

34. Drug name confusion

35. Dispensing error e.g. medication prepared incorrectly, even if it was not actually given to the participant

36. Drug not administered as indicated, for example, wrong route or wrong site of administration

37. Drug not taken as indicated e.g. tablet dissolved in water when it should be taken as a solid tablet

38. Drug not stored as instructed e.g. kept in the fridge when it should be at room temperature

39. Wrong participant received the medication (excluding IVRS/IWRS errors)

40. Wrong drug administered to participant (excluding IVRS/IWRS errors)

Examples of events that **do not** require reporting as medication errors in clinical studies:

41. Errors related to or resulting from IVRS/IWRS - including those which lead to one of the above listed events that would otherwise have been a medication error

42. Participant accidentally missed drug dose(s) e.g. forgot to take medication

43. Accidental overdose (will be captured as an overdose)

44. Participant failed to return unused medication or empty packaging

45. Errors related to background and rescue medication, or standard of care medication in open label studies, even if an AZ product

Medication errors are not regarded as AEs but AEs may occur as a consequence of the medication error.

APPROVED BY THE YALE UNIVERSITY IRB 2/12/2020

Appendix C Acceptable Birth Control Methods

Olaparib is regarded as a compound with medium/high foetal risk.

Women of childbearing potential and their partners, who are sexually active, must agree to the use of TWO highly effective forms of contraception in combination [as listed below]. This should be started from the signing of the informed consent and continue throughout the period of taking study treatment and for at least 6 months after last dose of study drug(s), or they must totally/truly abstain from any form of sexual intercourse (see below).

Male patients must use a condom during treatment and for 3 months after the last dose of olaparib when having sexual intercourse with a pregnant woman or with a woman of childbearing potential. Female partners of male patients should also use a highly effective form of contraception if they are of childbearing potential (as listed below). Male patients should not donate sperm throughout the period of taking olaparib and for 3 months following the last dose of olaparib.

Acceptable Non-hormonal birth control methods include:

- Total/True abstinence: When the patient refrains from any form of sexual intercourse and this is in line with their usual and/or preferred lifestyle; this must continue for the total duration of the trial and for at least 6 months after the last dose of study drug and for 3 months after last dose for male patients. [Periodic abstinence (e.g., calendar, ovulation, symptothermal, post-ovulation methods, or declaration of abstinence solely for the duration of a trial) and withdrawal are not acceptable methods of contraception]
- Vasectomised sexual partner PLUS male condom. With participant assurance that partner received post-vasectomy confirmation of azoospermia.
- Tubal occlusion PLUS male condom
- IUD PLUS male condom. Provided coils are copper-banded

APPROVED BY THE YALE UNIVERSITY IRB 2/12/2020

Acceptable hormonal methods:

- Normal and low dose combined oral pills PLUS male condom
- Cerazette (desogestrel) PLUS male condom. Cerazette is currently the only highly efficacious progesterone based pill.
- Hormonal shot or injection (eg., Depo-Provera) PLUS male condom
- Etonogestrel implants (e.g., Implanon, Norplant) PLUS male condom
- Norelgestromin / EE transdermal system PLUS male condom
- Intrauterine system [IUS] device (eg., levonorgestrel releasing IUS -Mirena®) PLUS male condom
- Intravaginal device (e.g., EE and etonogestrel) PLUS male condom

APPROVED BY THE YALE UNIVERSITY IRB 2/12/2020

Appendix D ECOG Performance Status

**ECOG Performance Status Scale**		**Karnofsky Performance Scale**	
Grade	Descriptions	Percent	Description
0	Normal activity. Fully active, able to carry on all pre-disease performance without restriction.	100	Normal, no complaints, no evidence of disease.
		90	Able to carry on normal activity; minor signs or symptoms of disease.
1	Symptoms, but ambulatory. Restricted in physically strenuous activity, but ambulatory and able to carry out work of a light or sedentary nature (*e.g.*, light housework, office work).	80	Normal activity with effort; some signs or symptoms of disease.
		70	Cares for self, unable to carry on normal activity or to do active work.
2	In bed <50% of the time. Ambulatory and capable of all self-care, but unable to carry out any work activities. Up and about more than 50% of waking hours.	60	Requires occasional assistance, but is able to care for most of his/her needs.
		50	Requires considerable assistance and frequent medical care.
3	In bed >50% of the time. Capable of only limited self-care, confined to bed or chair more than 50% of waking hours.	40	Disabled, requires special care and assistance.
		30	Severely disabled, hospitalization indicated. Death not imminent.
4	100% bedridden. Completely disabled. Cannot carry on any self-care. Totally confined to bed or chair.	20	Very sick, hospitalization indicated. Death not imminent.
		10	Moribund, fatal processes progressing rapidly.
5	Dead.	0	Dead.

APPROVED BY THE YALE UNIVERSITY IRB 2/12/2020

APPROVED BY THE YALE UNIVERSITY IRB 2/12/2020

Clinical Study Protocol 11/11/19 – version 3.1
TMZ + Olaparib for MGMT Hypermethylated Colorectal Cancer

APPROVED BY THE YALE UNIVERSITY IRB 2/12/2020
